# Therapy-induced senescence of glioblastoma cells is determined by the p21^CIP1^-CDK1/2 axis and does not require activation of DREAM

**DOI:** 10.1038/s41419-025-07651-8

**Published:** 2025-05-03

**Authors:** Christian Schwarzenbach, Justus Rinke, Juliana B. Vilar, Jason Sallbach, Larissa Tatsch, Ariane Schmidt, Anna Schöneis, Birgit Rasenberger, Bernd Kaina, Maja T. Tomicic, Markus Christmann

**Affiliations:** Department of Toxicology, University Medical Center of the Johannes Gutenberg University, Mainz, Germany

**Keywords:** Senescence, Chemotherapy, DNA damage response

## Abstract

Therapy-induced senescence (TIS) is a major challenge in cancer therapy as senescent cancer cells provoke local and systemic inflammation and might be the cause of recurrences. Elucidation of pathways leading to TIS is of utmost importance for establishing strategies to counteract this. Previously we have shown that temozolomide (TMZ), an alkylating drug used forefront in glioma therapy, causes majorly cellular senescence, which is triggered by the primary damage O^6^-methylguanine, activating the mismatch repair dependent ATR/ATM-CHK1/CHK2-p53 damage response pathway. The downstream pathways leading to TIS remained to be explored. Here, we show that TMZ-induced TIS in glioma cells does not require activation of the DREAM complex, but is bound on a G2-specific response. We show that the CDK inhibitor p21^CIP1^ does not interact with CDK4, but with CDK1 and CDK2 causing abrogation of the B-Myb and FOXM1-signaling pathway and subsequently arrest of cells in the G2-phase. The induced G2-arrest is incomplete as DNA synthesis can be resumed leading to endoreduplications. This process, which is inhibited by the CDK4-blocking drug palbociclib, is preceded by reactivation of the G1/S-specific E2F1-signaling pathway due to lack of functional DREAM activation. These findings provide an explanation for the polyploidization and giant cell phenotype of anticancer drug-induced senescent cells. Incomplete DREAM activation may also explain the observation that downregulation of DNA repair is a transient phenomenon, which goes along with the entrance of cells into the senescent state.

## Introduction

Cellular senescence is defined as an irreversible cell cycle arrest and inability of cells to respond to proliferative conditions (for review see [1–4]). During normal aging, replicative senescence occurs in cells that reach the Hayflick limit [5] and is caused by telomere shortening during cell divisions. Cellular senescence is also induced during tumorigenesis through proliferation-associated signals (oncogenic senescence) and by genomic stress induced by external and internal DNA-damaging agents. A specific type of DNA damage-induced senescence is therapy-induced senescence (TIS), which is triggered by genotoxic anticancer drugs and ionizing radiation during cancer therapy [6].

Hallmarks of cellular senescence are sustained activation of the DNA damage response (DDR) resulting in a permanent cell cycle arrest *via* inhibition of cyclin-dependent kinases (CDKs) through the CDK inhibitors *CDKN1a*/p21^CIP1^, *CDKN2a*/p14^ARF^ and *CDKN2a*/p16^INK4a^. An important downstream target of p21^CIP1^ is the DREAM complex, which arrests cells in the G0-phase and controls p53-mediated gene repression. In this case, p53 does not bind directly to promoter regions of the repressed genes, but causes through transcriptional induction of p21^CIP1^ an activation of the dimerization partner, RB-like, E2F, and multi-vulval class B (DREAM) complex [7–11]. The core DREAM complex is formed by the MuvB complex. During cell cycle progression, B-Myb (encoded by *MYBL2*) and/or FOXM1 bind to the MuvB complex and thus activate genes involved in proliferation. Upon p21^CIP1^ induction, CDK4 becomes inactivated, leading to dephosphorylation of p105 and p130 that bind together with E2F4 and E2F5 to the MuvB complex, forming the DREAM complex. In contrast to E2F1, E2F4 and E2F5 are transcriptional repressors, which silence the expression of proliferation-associated factors. Besides CDK4, p21^CIP1^ can also inhibit CDK1 and CDK2 [12] and is thereby involved in cell cycle arrest in the G1- and G2-phase, independent of DREAM.

We have shown that the anticancer drug temozolomide (TMZ), via induction of O^6^-methylguanine (O^6^MeG), triggers apoptosis [13], autophagy, and senescence in glioma cells [14, 15], with senescence being the main trait [16, 17]. TMZ is administered in the first-line therapy of high-grade gliomas [18–20], with glioblastomas (GBM) being incurable due to acquired resistance and formation of recurrences [21]. To study the mechanism of TMZ-induced senescence in GBM cells, we analyzed the transcriptional response, interaction of p21^CIP1^ with CDKs, and post-translational modification of cell cycle control mechanisms. Our data revealed that TMZ does not activate the DREAM complex, but rather induces an incomplete G2-arrest, which enables cells to re-enter the S-phase, causing endoreduplications and the formation of polyploid giant cells. Additional inhibition of CDK4 by palbociclib caused a stable G2-arrest and prevented polyploidy. These findings bear therapeutic implications as CDK4/CDK6 inhibitors may foster TMZ-induced senescence and thereby reduce senescence evasion.

## Materials and methods

### Cell culture, drug treatment, pharmacological inhibition and p21^CIP1^ knockdown

The glioma cell line LN229 (RRID:CVCL_0393) was obtained from LGC Standards (Wesel am Rhein, Germany). MCF7 (RRID:CVCL_0031), A172 (RRID:CVCL_0131), and U87MG (RRID:CVCL_0022; since misidentified, it refers to as “glioblastoma of unknown origin”) were purchased from Cell Line Service (Eppelheim, Germany). RPE1 cells (RRID:CVCL_4388) were obtained from ATCC. LN308 cells were a generous gift of Prof. Michael Weller (University of Zürich, Switzerland). All cell lines were cultivated in Dulbecco’s minimal essential medium (DMEM) supplemented with 10% fetal bovine serum at 37 °C in a humidified atmosphere containing 5% CO_2_ and were regularly checked for mycoplasma contamination (Venor GeM Cassic kit, Minerva Biolabs). All cell lines besides MCF7 are deficient for MGMT and besides LN308 are proficient for p53 [22, 23]. TMZ was kindly provided by Prof. Geoff Margison, Centre for Occupational and Environmental Health, University of Manchester, United Kingdom, and the CDK4 inhibitor palbociclib (S4482) was purchased from Selleckchem. Oxaliplatin and irinotecan (CPT-11) were prepared by the pharmacy of the University Medical Center Mainz, Germany. For gene silencing, predesigned siRNAs specific for p21^CIP1^ (sc-29427, Santa Cruz Biotechnology) were used; control human non-silencing siRNA (Silencer Select Predesigned siRNA Negative Control #1 siRNA; Ambion) was used as negative control. Transfections were performed using Lipofectamine RNAiMAX Reagent (Invitrogen).

### Cell cycle progression and senescence

To quantify TMZ-induced cell cycle distribution, cells were stained with propidium iodide (PI). The DNA content was determined by flow cytometry using BD FACSCanto™ II. Senescence was measured microscopically by SA(senescence-associated)-β-Gal staining or using C_12_FDG stainig and flow cytometry as described before [14]. Experiments were performed as biological triplicates.

### Cell proliferation and DNA synthesis

To measure cell proliferation, LN229 cells were seeded at a density of 50.000 cells in 6-cm dishes. Six hours later, the attached cells were harvested and counted to obtain the starting number for cell counts. In parallel, cells were treated with 50 µM TMZ or left untreated. After additional 24–144 h, cells were harvested and counted. To measure DNA synthesis, LN229 cells were seeded into a 96-well microtiter plate. Six hours later, cells were treated with 50 µM TMZ or left untreated. After additional 24–144 h, DNA synthesis was measured by the colorimetric BrdU ELISA assay (Roche). Experiments were performed as biological triplicates.

### Preparation of RNA, real-time qPCR, methylation specific PCR and RNA-Seq

Total RNA was isolated using the NucleoSpin^®^ RNA Kit from Macherey-Nagel (Düren, Germany). Utilizing the Verso cDNA Kit (Thermo Scientific, Dreieich, Germany), 1 µg total RNA was transcribed into cDNA and qPCR was performed in technical triplicates using the GoTaq^®^ qPCR Master Mix Protocol (Promega, Madison, USA) and the CFX96 Real-Time PCR Detection System (Biorad, München, Germany). Analysis was performed using the CFX Manager^TM^ software, and the expression was normalized to *gapdh* and *β-actin*. Gene espression of the untreated control was set to one. Experiments were performed as technical triplicates. Primers sequences are listed in Table S1. For methylation specific PCR (MSP), DNA was extracted using phenol-chloroform extraction. Modification of the DNA was performed using the EZ DNA Methylation Kit (Zymo Research) as described [23]. p14^ARF^ (Esteller et al. [24]) and p16^INK4A^ (Herman et al. [25]) specific primers have been published before. As positive control for the reaction, the β-actin promoter was amplified. All primers sequences are listed in Table S1. For RNA-Seq, NGS library prep was performed with Illumina’s TruSeq stranded mRNA LT Sample Prep Kit following TruSeqStrandedmRNAReferenceGuide (Oct. 2017) (Document # 1000000040498v00). Libraries were prepared with a starting amount of 1000 ng and amplified in 10 PCR cycles. Libraries were profiled in a High Sensitivity DNA on a 2100 Bioanalyzer (Agilent technologies) and quantified using the Qubit dsDNA HS Assay Kit, in a Qubit 2.0 Fluorometer (Life technologies). All 12 samples were pooled in equimolar ratio and sequenced on NextSeq 500 Highoutput FC, PE for 2 × 150 cycles plus 7 cycles for the index read.

### Gene sets used for evaluation of NGS data

The following gene sets were obtained from the Molecular Signatures Database (MSigDB database v7.5.1) [26]: FISCHER_DREAM_TARGETS, FISCHER_DIRECT_P53_TARGETS_META_ANALYSIS, FISCHER_G1_S_CELL_CYCLE, FISCHER_G2_M_CELL_CYCLE [27]; E2F4DP1_01; E2F5_TARGET_GENES [28]; SHEPARD_BMYB_TARGETS [29], GOBP_MITOTIC_NUCLEAR_DIVISION, GOBP_MICROTUBULE_CYTOSKELETON_ORGANIZATION. FOXM1 and E2F-RB target genes were directly obtained from Fischer et al. [27]. Venn diagrams were drawn using the program from (http://bioinformatics.psb.ugent.be/webtools/Venn).

### Preparation of protein extracts, western blot analysis, co-immunoprecipitation and interactomics

Whole-cell extracts were prepared as described [30]. The specific antibodies are listed in Table S2. Co-immunoprecipitation was performed as already described [30, 31].

### Xenograft experiments

The experiments were performed in accordance with relevant institutional and national guidelines and regulations, approved by the Landesuntersuchungsamt Rheinland-Pfalz, Germany (23 177-07/041-15V2). The experimental details were published previously [14] and summarized in the Legend to Fig. S11.

### Quantification and statistical analysis

Statistical analyses were performed using GraphPad Prism version 6.01 for Windows, GraphPad Software, La Jolla, California USA (www.graphpad.com). Data were evaluated using Student’s *t*-test and were expressed as a mean ± SD; *p ≤ 0.05 was considered statistically significant, **p ≤ 0.01 very significant, and ***p ≤ 0.001 highly significant.

## Results

### Phenotype of TMZ-induced senescence

**Fig. 1:**
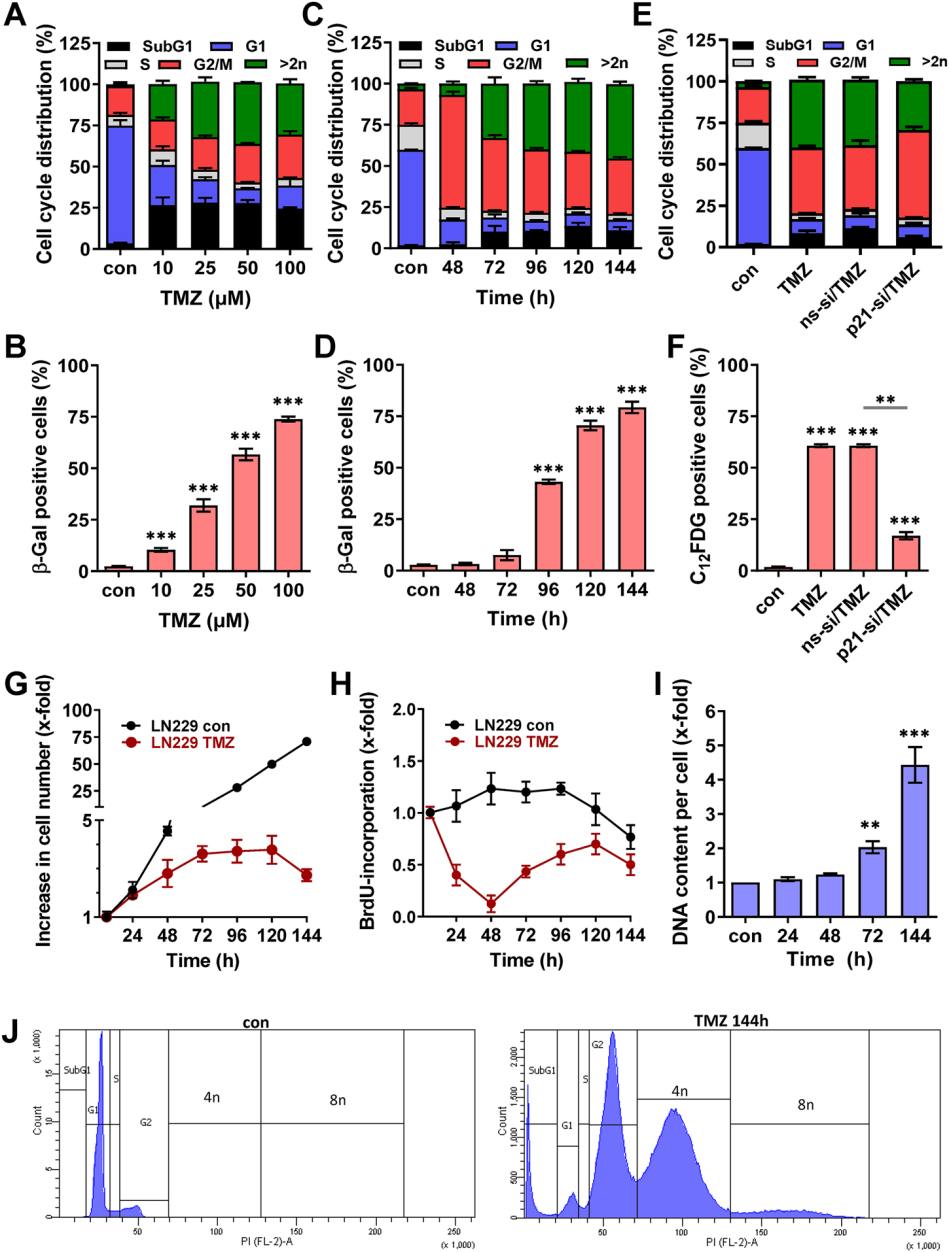
TMZ-induced cell death and senescence. **A**–**D** LN229 cells were exposed to different concentrations of TMZ for 144 h (**A**, **B**) or to 50 µM TMZ for different time periods (**C**, **D**). Experiments were performed in triplicates. **A**, **C** Cell death was measured by flow cytometry using PI staining. **B**, **D** Frequency of senescent cells was detected microscopically by SA-β-Gal staining. **E**, **F** LN229 cells were transfected with p21^CIP1^ specific siRNA or nonspecific siRNA and 24 h later exposed to 50 µM TMZ for 144 h. Cell death was measured by flow cytometry using PI staining (**E**) and frequency of senescent cells was detected by flow cytometry using C_12_FDG staining (**F**). **G**–**I** LN229 cells were exposed to 50 µM TMZ for different time periods. **G** Proliferation was measured by cell counting. **H** DNA synthesis was measured by BrdU assay. **I** DNA content per cell was measured using the NanoDrop 1000 Spectrophotometer. **J** LN229 cells were exposed to 50 µM TMZ for 144 h, and cell cycle distribution was measured by flow cytometry using PI staining. A representative experiment is shown. **B**, **D**, **I** Differences between treatment and control were statistically analyzed using Student’s *t* test (*p* < 0.001). **F** Differences between treatment and control, as well as between ns-si/TMZ and p21-si/TMZ were statistically analyzed using Student’s *t* test (**p < 0.01***p < 0.001).

The alkylating drug TMZ induced cell death (Fig. 1A, C) and, predominantly, cellular senescence (Fig. 1B, D). Senescence was associated with an arrest of cells in the G2-phase and an increase in the DNA content (Fig. 1A). The G2-arrest was observed already 48 h after TMZ treatment (Fig. 1C), whereas the number of cells with increased DNA content (>2n) went up later with time. They were first observed after 72 h and reached a level of 45% 144 h after treatment with 50 µM (Fig. 1C). This went along with the appearance of the senescence phenotype (Fig. 1D). Whereas the initial G2-arrest following TMZ treatment was not affected by knockdown of p21^CIP1^ and, therefore, is independent of p21^CIP1^ (Fig. 1E), the senescence phenotype was strongly affected and thus is p21^CIP1^ dependent (Fig. 1F). In line with this, knockdown of p21^CIP1^ had no impact on proliferation, but slightly increased the cell death level after late times (2 weeks), indicating killing of senescent cells (Fig. S1A–C). Proliferation assays indicated that cells could still perform two cycles of proliferation before they were blocked 48-72 h after TMZ exposure (Fig. 1G). However, despite the proliferation arrest, cells were able to restart DNA-synthesis 72 h after TMZ (Fig. 1H) and, as a consequence, showed an increased DNA content (Fig. 1I). There are two major pathways that can explain the observed over-replication, namely endoreduplication and re-replication [32]. During re-replication, origins can fire more than once within a single S-phase while during endoreduplication, cells circumvent mitosis entering a G1-like state and re-enter the S-phase, leading to polyploid mononucleated cells. Endoreduplication gives rise to discrete peaks in DNA content corresponding to 4n and 8n, which is different from re-replication. In the case of TMZ-induced senescence, a clear 4n and even an 8n peak (Fig. 1J) as well as polyploid mononucleated cells (Fig. S1D) were observed, clearly pointing to endoreduplication, and not re-replication to occur.

### TMZ does not cause activation of DREAM

**Fig. 2:**
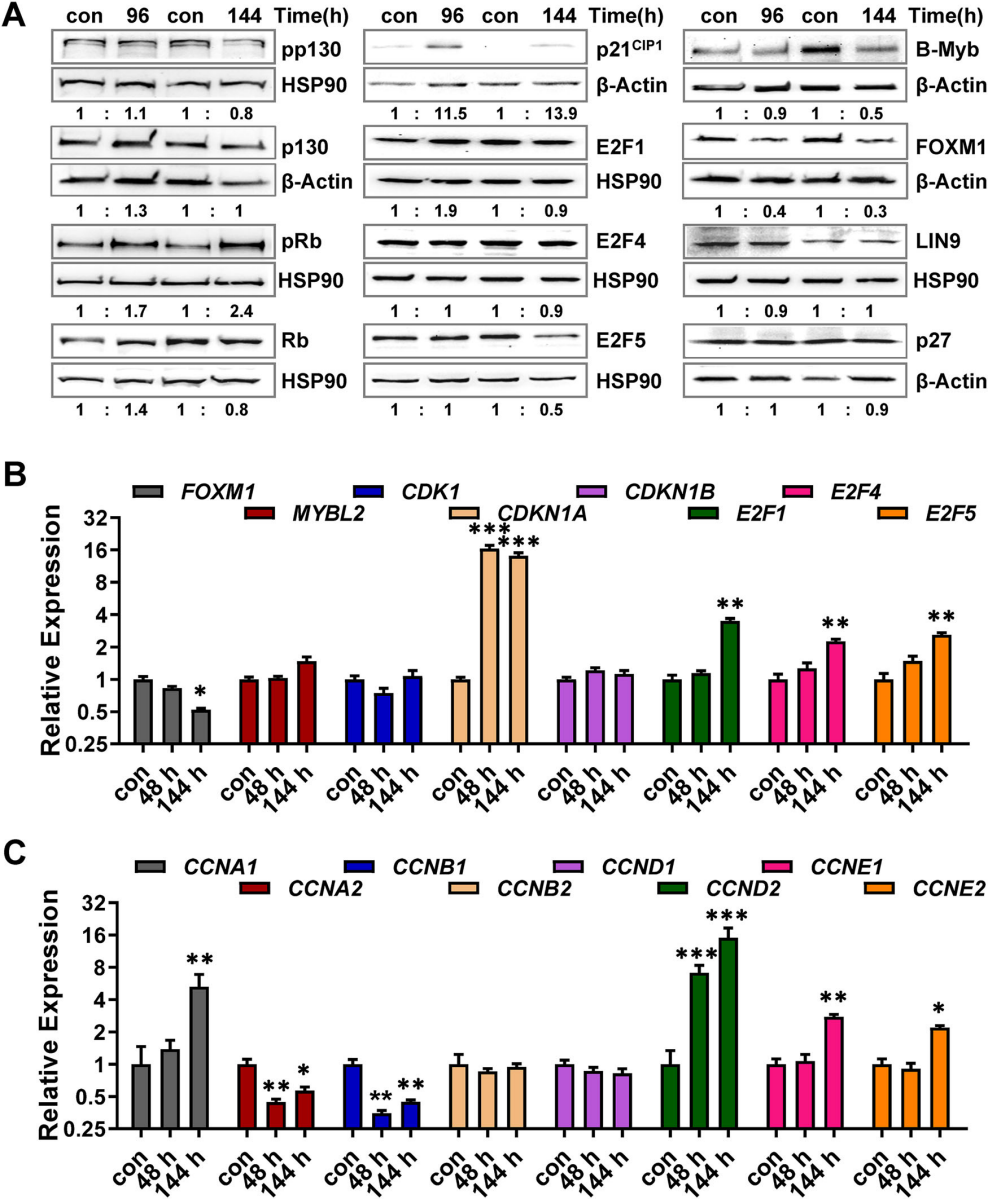
Analysis of DREAM activation. **A** LN229 cells were exposed to 50 µM TMZ for 96 or 144 h. Expression and phosphorylation of p130 (pp130), Rb (pRb), as well as expression of p21^CIP1^, E2F1, E2F4, E2F5, B-Myb, FOXM1, LIN9, and p27 were measured by immunoblotting. β-Actin or HSP90 was used as internal loading control. Quantification of the immunoblot indicates x-fold induction in TMZ-exposed cells compared to untreated cells. **B**, **C** LN229 cells were exposed to 50 µM TMZ for 48 or 144 h. **B** Expression of *FOXM1*, *MYBL2*, *CDK1*, *CDKN1A*, *CDKN1B*, *E2F1*, *E2F4* and *E2F5* was measured by qPCR. **C** Expression of *CCNA1*, *CCNA2*, *CCNB1*, *CCNB2*, *CCND1*, *CCND2*, *CCNE1* and *CCNE2* was measured by qPCR. **B**, **C** Experiments were performed in triplicates, *ACTB* and *GAPDH* were used as internal loading control. Differences between the control and TMZ treatment were statistically analyzed using Student’s *t* test (non-labeled = not significant; *p < 0.1; **p < 0.01; ***p < 0.001).

To analyze whether the DREAM complex is activated following TMZ treatment, the expression and phosphorylation of p130 and RB as well as the expression of p21^CIP1^, E2F1, E2F4, E2F5, B-Myb, FOXM1, LIN9 and p27 were analyzed 96 and 144 h after TMZ exposure, when cells reached the senescent stage (Fig. 2A). The cells in the population showed no significant reduction in the phosphorylated form of RB and p130, indicating the DREAM complex was not activated. This result was unexpected, giving the fact that p21^CIP1^ was strongly induced after genotoxic exposure. For E2F1, E2F4, E2F5, LIN9, and p27 no changes in the expression were observed and, in case of FOXM1 and B-Myb, decreased expression was observed 144 h after TMZ treatment.

Besides protein expression, we analyzed the expression of *FOXM1*, *MYBL2*, *CDK1*, *CDKN1A*, *CDKN1B*, *E2F1*, *E2F4* and *E2F5* 48 and 144 h after TMZ on transcriptional level (Fig. 2B), revealing a strong induction of *CDNK1A* at all time points and a weak induction of *E2F1, E2F4,* and *E2F5*. The expression of *MYBL2* and *CDKN1B* was unaltered, and *FOXM1* was repressed 144 h after treatment. To gain further insight into the cell cycle regulation following TMZ, we also analyzed the expression of different cyclins (Fig. 2C). The data showed a strong repression of *CCNA2* and *CCNB1*, which is in line with the arrest of cells in G2. They also showed induction in *CCNA1, CCND2*, *CCNE1,* and *CCNE2*, indicating an increased G1/S activity, which could explain the endoreduplications observed in the cell population.

### Transcriptomics reveals a G2/M-specific response

**Fig. 3:**
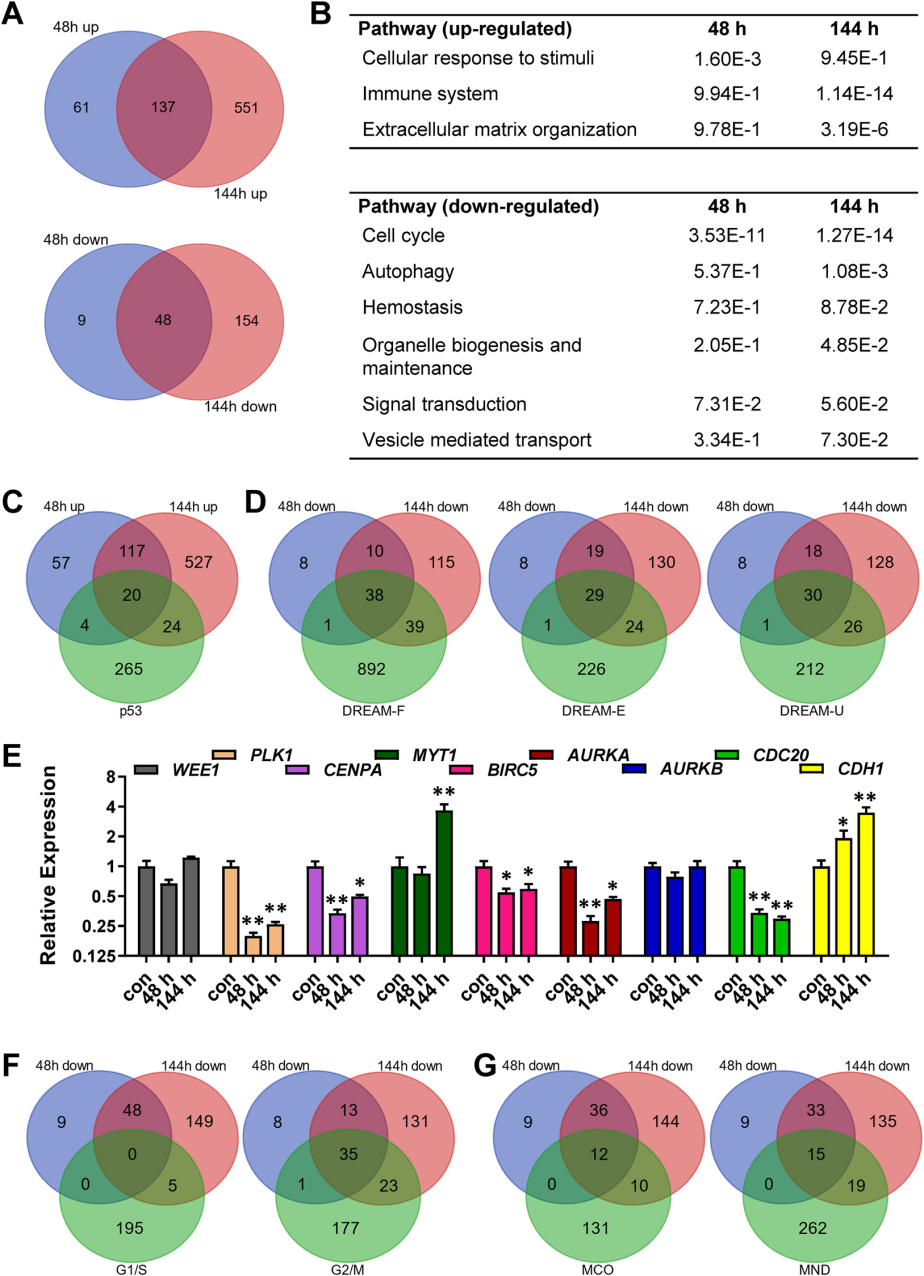
Transcriptional changes upon TMZ exposure. LN229 cells were exposed to 50 µM TMZ for 48 or 144 h. RNA was isolated and subjected to RNA-Seq. **A** The genes commonly up-regulated (upper panel) or down-regulated (lower panel) after 48 and 144 h exposure are displayed as Venn diagrams. **B** Significantly regulated pathways were identified by the Reactome pathway browser (https://reactome.org/PathwayBrowser/). **C** The up-regulated genes and overlap with p53 targets are displayed as Venn diagrams. **D** The down-regulated genes and overlap with DREAM targets are displayed as Venn diagrams. **E** LN229 cells were exposed to 50 µM TMZ for 48 or 144 h. Expression of WEE1, PLK1, CENPA, MYT1, BIRC5, AURKA, AURKB, CDC20 and CDH1 was measured by qPCR. Differences between the control and TMZ treatment were statistically analyzed using Student's *t* test (non labelled = not significant; *p < 0.001 and log2 fold change > 1.5 are included.

To gain more insight into the transcriptional response upon TMZ, we performed NGS-based transcriptomics 48 and 144 h after TMZ treatment of LN229 cells. The results indicate 198 genes to be induced after 48 h and 688 genes after 144 h, including 137 genes to be upregulated at both time points (Fig. 3A). Pathway analysis identified *cellular response to stimuli* as sole induced pathway after 48 h. After 144 h a strong induction of the *immune system* and of *extracellular matrix organization* was observed (Fig. 3B, upper table). Concerning down-regulated genes, 57 were repressed after 48 h and 277 after 144 h, including 48 genes repressed at both time points (Fig. 3A). Concomitantly, a drastic repression of genes belonging to the *cell cycle* pathway was observed (Fig. 3B, lower table). Additional pathway analysis using the WEB-based GEne SeT AnaLysis Toolkit (http://www.webgestalt.org) and the Reactome and Kegg database are shown in Fig. S2.

Among the upregulated genes, several p53-regulated genes were identified (Fig. 3C), among them *CDKN1A*, *MDM2,* and *TP53INP1* (Fig. S3A). Concerning DREAM targets, several gene sets exist. Using the DREAM-set derived from Fischer [27] (DREAM-F), 39 genes were repressed after 48 h and 77 after 144 h, including 38 at both time points (Figs. 3D and S3B). Using the DREAM-set derived from Engeland [33] (DREAM-E), 30 genes were repressed after 48 h and 53 after 144 h, including 29 at both time points and using the DREAM-set derived from Uxa [11] (DREAM-U), 31 genes were repressed after 48 h and 56 after 144 h, including 30 at both time points. Among the DREAM targets repressed at both time points, we identified important cell-cycle regulating factors like *AURKA, BIRC5, BUB1*, CCNA1, *CCNB1*, *CDC20*, *CENPA,* and *PLK1*. After 144 h, also *AURKB*, *BORA*, *CCNB2,* and *FOXM1* were repressed (Fig. S3B).

The observed repression of important regulators of the G2/M-phase was further analyzed by qPCR (Fig. 3E). Similar to the results of the transcriptomic approach, a strong repression of *AURKA, CDC2*0, *CENPA, PLK1,* and a weaker repression of *BIRC5* was observed. Opposite, an increased expression of *CDH1* was observed after 48 and 144 h and of *MYT1* after 144 h; the expression of *AUKB* and *WEE1* changed only marginal.

Similar to the expression data of the cyclins, the transcriptomic data suggested specific alterations in the G2/M-phase. Thus, among the repressed genes, only 5 were G1/S-specific whereas 59 were G2/M-specific (Fig. 3F). Importantly, most of the repressed G2/M, but not G1/S genes, were DREAM targets (Fig. S4A), which contradicts the normal distribution among G1/S and G2/M-specific genes (Fig. S4B). Upon TMZ exposure, using all DREAM-sets, only one gene was G1/S-specific, whereas 44 (DREAM-E/F) and 45 (DREAM-U) genes were G2/M-specific (Figs. S4C and S5). At the first glance, the high number of repressed DREAM targets hints at a DREAM response, however, this response is not characterized by the typical repression of cardinal G1/S-specific DREAM targets.

These non-repressed G1/S-specific DREAM targets included important cell cycle regulators like *CDK1* and *E2F1*. Moreover, B-Myb was also described as G1/S-specific gene [34], however is not part of the used G1/S-specific gene set. Interestingly, a high frequency of repressed genes was associated with mitosis. Thus 22 genes were associated with *microtubule cytoskeleton organization* and 34 with *mitotic nuclear division*, which include *AURKA*, *AURKB*, *BUB1*, *BORA*, *CCNB1*, *CDC20,* and *PLK1* (Figs. 3G and S6).

**Fig. 4:**
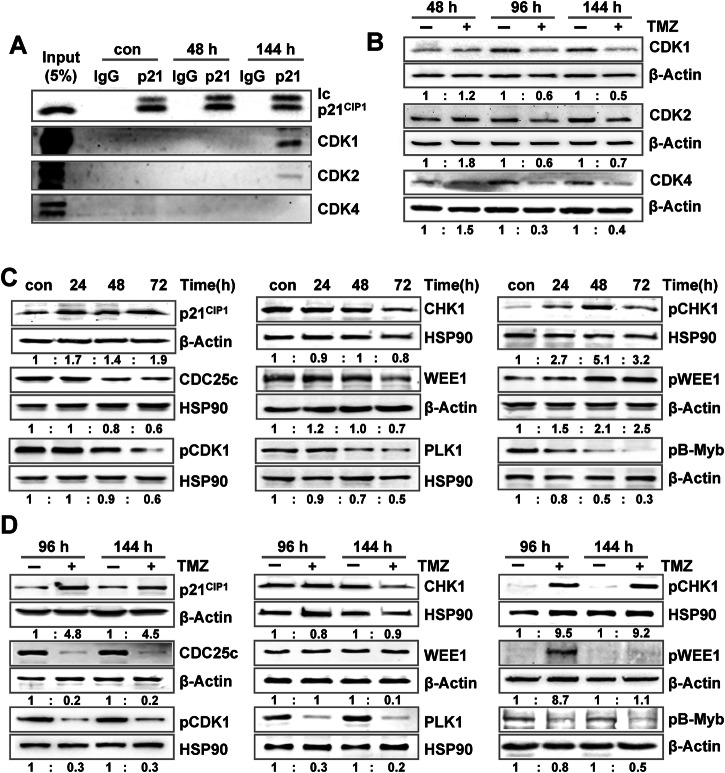
Binding of p21^CIP1^ to CDKs and activation of the DDR. **A** LN229 cells were exposed to 50 µM TMZ for 48 or 144 h. Interaction between p21^CIP1^ and CDK1, CDK2, and CDK4 was measured by co-immunoprecipitation. **B** LN229 cells were exposed to 50 µM TMZ for 48, 96, or 144 h. Expression of CDK1, CDK2, and CDK4 was measured by immunoblotting. β-Actin was used as internal loading control. **C** LN229 cells were exposed to 50 µM TMZ for 24, 48, or 72 h. **D** LN229 cells were exposed to 50 µM TMZ for 96 or 144 h. **C**, **D** Expression of p21^CIP1^, CDC25c and PLK1, as well as expression and phosphorylation of CDK1 (pCDK1), CHK1 (pCHK1), WEE1 (pWEE1) and B-Myb (pB-Myb) was measured by immunoblotting. β-Actin or HSP90 were used as internal loading control. **B**, **C** Quantification of the immunoblot indicates x-fold induction in TMZ exposed cells compared to untreated cells.

The missing activation of the DREAM complex as well as the missing repression of G1/S-specific genes in TMZ-treated LN229 cells was quite unexpected, giving the strong activation of p21^CIP1^ and the observed repression of multiple G2/M-specific DREAM targets. Therefore, we analyzed the interaction between p21^CIP1^ and the main CDKs by co-immunoprecipitation (Fig. 4A). The results showed binding of p21^CIP1^ to CDK1 and less pronounced to CDK2, but not to CDK4. Of note, this binding was only observed at late time points (144 h), but not early (48 h) after TMZ, indicating that the initial cell cycle arrest does not depend on p21^CIP1^ mediated CDK inhibition. Concomitantly, we also analyzed the expression of the CDKs and observed a slightly decreased expression of all CDKs at late times (96 h, 144 h) after exposure (Fig. 4B).

### TMZ-induced senescence is regulated by silencing of B-Myb and FOXM1 signaling

The missing involvement of the DREAM complex raised the question of how the cell cycle arrest was provoked and maintained after TMZ treatment. Therefore, protein expression and phosphorylation of important cell cycle regulators were analyzed 24–72 h after TMZ exposure (Fig. 4C). In the undisturbed cell cycle, CDK2 together with Cyclin E forms a complex at the end of the G1-phase that allows entry into the S-phase (for review see [35]). On one hand, it supports the function of Cyclin D/CDK4/CDK6 by additional phosphorylation of RB, and on the other hand, activates proteins involved in DNA replication *via* activation of the transcription factor B-Myb. If CDK2 is defective, CDK1 can take over the function. In this case, progression through the G2-phase and onset of mitosis is regulated *via* CDK1, which depends on its dephosphorylation mediated by the phosphatase CDC25c, as well as on interaction with Cyclin B1. Activation of CDC25c is mediated *via* PLK1-mediated phosphorylation. Both, Cyclin B1 and PLK1 are transcriptionally activated by B-Myb. In response to DNA damage, CDC25c undergoes proteasomal degradation upon phosphorylation by CHK1. Activated CHK1 can also phosphorylate/activate WEE1, which acts as CDK1 inhibitor.

In accordance to this background information, we observed phosphorylation/activation of CHK1 and WEE1 and degradation of CDC25c (Fig. 4C). Moreover, reduced phosphorylation of CDK1 and B-Myb was observed. This was in contrast to p21^CIP1^, which only showed a weak induction at early times (<72 h) after TMZ exposure. The results clearly indicate that the early cell cycle arrest is mediated by posttranslational modifications of CHK1 and WEE1, which is provoked by the DDR pathway independent of p21^CIP^. Dephosphorylation of CDK1 and B-Myb as well as degradation of CDC25c and repression of PLK1 was still observed at late times after damage induction (96 and 144 h), whereas the activated form of pWEE1 was missing (Fig. 4D). As indicated by co-immunoprecipitation experiments, at this time point p21^CIP1^ takes over the role from WEE1 in inhibiting CDK1 and CDK2.

Overall, the data indicate that upon TMZ-induced DNA damage, dephosphorylation and thereby inactivation of B-Myb as well as repression of FOXM1 is of outmost importance for arresting cells in the cell cycle and for transcriptional repression. During an undisturbed cell cycle progression, B-Myb is phosphorylated by CDK2 and acts together with FOXM1 as transcription factor for G2/M-specific genes (for review see [35]). At later times, B-Myb is removed from the MuvB complex and FOXM1 acts as the sole transcription factor regulating mitosis. This is also reflected by our transcriptomics data. It is well known that the DREAM complex silences genes normally induced by E2F1, B-Myb, and FOXM1, explaining the overlapping target-gene sets (Fig. S7A). Therefore, the transcriptional response in TMZ-treated cells can be explained by DREAM-independent silencing of the B-Myb and FOXM1 response. Indeed, the majority of targets of the observed transcriptional response are also targets of B-Myb and FOXM1 (Fig. S7B).

**Fig. 5:**
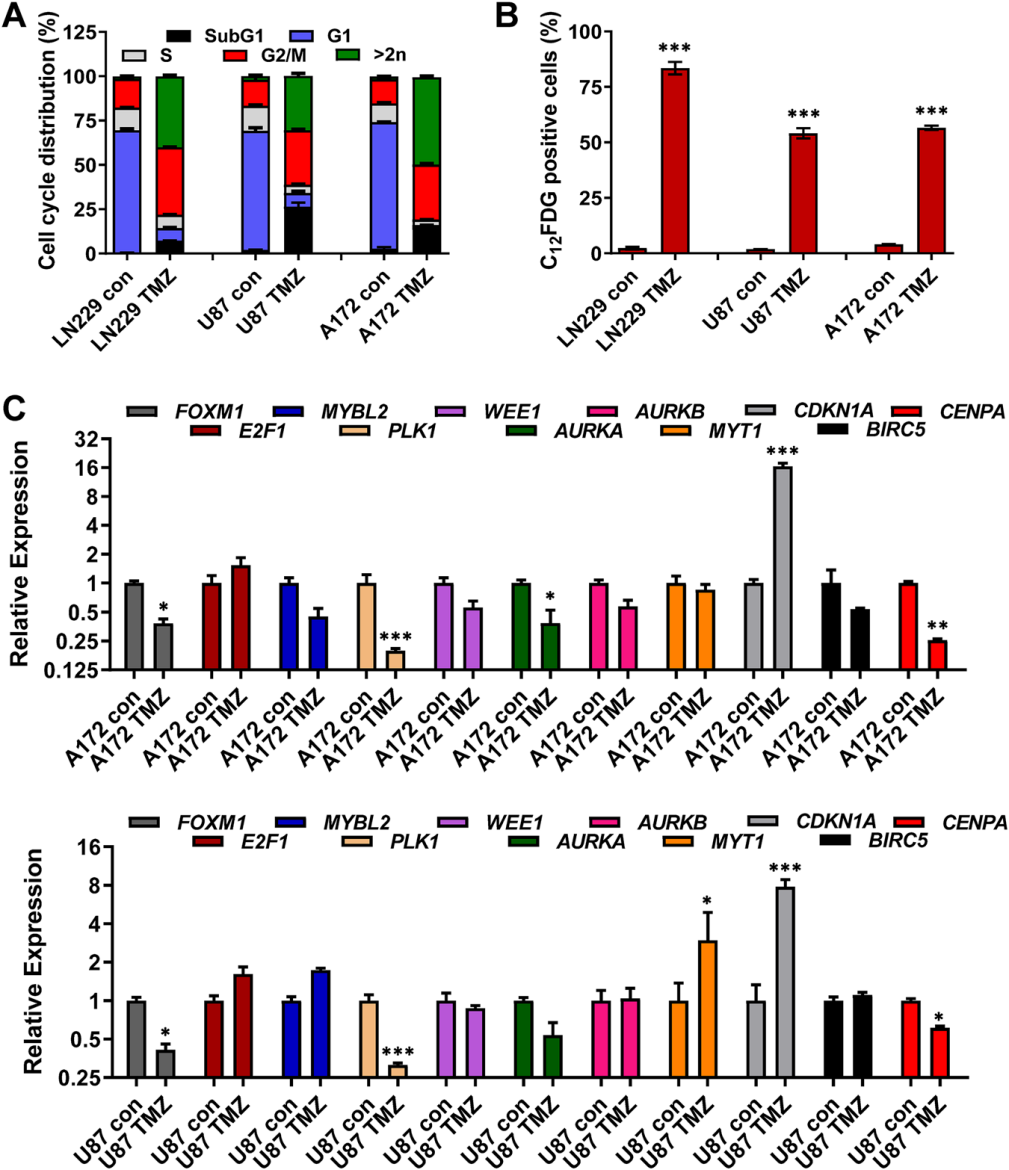
TMZ-induced cell death, senescence and transcriptional regulation of cell cycle factorsin A172 and U87MG cells. **A**, **B** LN229, U87MG, and A172 cells were exposed to 50 µM TMZ for 144 h. **A** Cell death and cell cycle distribution were measured by flow cytometry using PI staining. **B** Frequency of senescent cells was measured by flow cytometry using C_12_FDG staining. **C** A172 and U87MG cells were exposed to 50 µM TMZ for 144 h. Expression of *FOXM1*, *E2F1*, *MYBL2*, *PLK1*, *WEE1*, *AURKA*, *AURKB, MYT1*, *CDKN1A*, *BIRC5* and *CENPA* was measured by qPCR. **A**–**C** Experiments were performed in triplicates. **B**–**C** Differences between treatment and control were statistically analyzed using Student’s *t* test (not-labeled = not significant; *p < 0.1; **p < 0.01; ***p < 0.001).

To test whether the missing activation of the DREAM response is a common event in glioblastoma, we extended the study to two other glioma cell lines (A172 and U87MG). Similar to LN229 cells, A172 and U87MG cells express functional p53 and show a robust induction of p21^CIP1^ upon TMZ (Fig. S8A–D) [14, 36]. Furthermore, all cell lines are deficient for CDKN2A (p14^ARF^ and p16^INK4a^) due to deletion mutations (Fig. S8E). In these cells, TMZ strongly induced cellular senescence while only a low amount of apoptosis was detected (Fig. 5A, B). Similar to LN229, a high level of cells harboring a polyploid DNA content (>2n) was observed. We also analyzed the expression of factors involved in the senescence-associated secretory phenotype (SASP). In all cell lines, a strong upregulation of *CCL2, CCL8, CCXL1, IL1α, IL1β, IL6,* and *IL8* was observed in the senescent population (Fig. S9). The analysis of cell cycle regulating genes revealed an expression pattern similar to LN229 cells. Thus, a repression of *AURKA*, *CCNA2*, *CCNB1*, *CENPA, FOXM1,* and *PLK1* and an induction of *E2F1* were observed in A172 and U87MG cells (Fig. 5C).

**Fig. 6:**
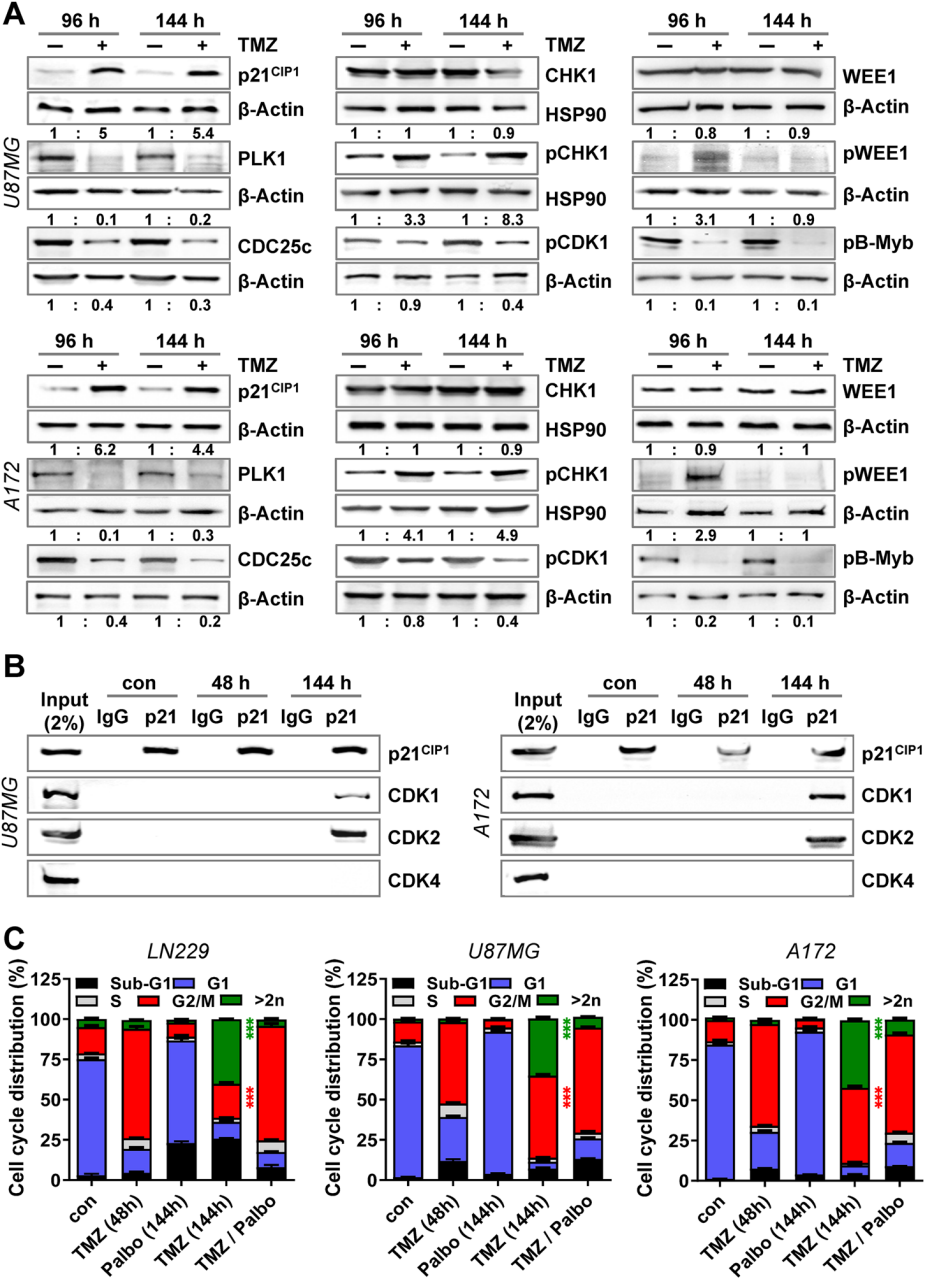
Binding of p21^CIP1^ to CDKs and activation of the DDR in A172 and U87MG cells. **A** U87MG and A172 cells were exposed to 50 µM TMZ for 96 or 144 h. Expression of p21^CIP1^, CDC25c, and PLK1, as well as expression and phosphorylation of CDK1 (pCDK1), CHK1 (pCHK1), WEE1 (pWEE1) and B-Myb (pB-Myb) were measured by immunoblotting. β-Actin or HSP90 was used as an internal loading control. Quantification of the immunoblot indicates x-fold induction in TMZ-exposed cells compared to untreated cells. **B** U87MG and A172 cells were exposed to 50 µM TMZ for 48 or 144 h. Interaction between p21^CIP1^ and CDK1, CDK2, and CDK4 was measured by co-immunoprecipitation. **C** LN229, U87MG, and A172 cells were exposed to 50 µM TMZ for 48 or 144 h. In addition, the cells were exposed to 1 µM Palbociclib for 144 h or the cells were exposed to 50 µM TMZ, and 48 h later 1 µM Palbociclib was added for additional 96 h (TMZ/Palbo). Cell death and cell cycle distribution were measured by flow cytometry using PI staining; experiments were performed in triplicates. Differences between the frequency of cells in the G2-phase or showing a DNA content >2*n* after TMZ and TMZ/Palbo treatment were statistically analyzed using Student’s *t* test (***p < 0.001).

Similar to LN229, the initial cell cycle arrest in U87MG and A172 cells was mediated by post-translational modifications (Fig. 6A), whereas the final senescence-associated cell cycle arrest was dependent on p21^CIP1^ as indicated by co-immunoprecipitation with CDK1 and CDK2, but not with CDK4 during the senescence maintenance phase (Fig. 6B). Also, in A172 and U87MG cells, the inhibition of CDK1 and CDK2 was accompanied by repression of *CCNA1/2* and *CCNB1/2*, whereas induction of *CCND1, CCND2* and, in U87MG, also of *CCNE1* and *CCNE2* was found (Fig. S10).

To substantiate the data, the activation of the DREAM complex and regulation of DREAM targets were analyzed in mouse U87MG xenograft experiments 96 h after TMZ exposure (Fig. S11). Immunodetection revealed the induction of p21^CIP1^, but no reduced phosphorylation of p130 and RB1. Furthermore, repression of G2/M-specific DREAM targets (e.g., *AURKA*, *CCNB1*, *PLK1*), but not G1/S-specific DREAM targets (e.g., *E2F1*, *MSH2*, *MSH6*) was observed. Overall, the data indicate that also in vivo, in human glioma xenografts, the DREAM complex is not fully activated.

### Lack of DREAM activation leads to endoreduplications

To test whether missing inhibition of CDK4 and abrogated DREAM activation might explain the formation of endoreduplications and polyploid cells, we inhibited CDK4/6 by palbociclib, 48 h after drug treatment. At this time point the cells were arrested in G2, but did not show endoreduplication yet (Fig. 6C). Indeed, pharmacological CDK4/6 inhibition completely abrogated the expression of the cell cycle regulators *E2F1*, *FOXM1* and *MYBL2* in all cell lines (Fig. S12) and strongly reduced the number of cells performing endoreduplications (Fig. 6C).

An important question is whether missing activation of DREAM and accompanied endoreduplication is cell type- or agent-specific. Therefore, we compared the response of LN229 and the breast cancer cell line MCF7, as well as non-tumorigenic RPE1 (hTERT-immortalized retinal pigment epithelial) cells to TMZ, irinotecan, and oxaliplatin. With all agents examined, LN229 but not MCF7 or RPE1 cells showed endoreduplications that indicated missing DREAM activation (Fig. S13A). Most importantly, senescence-inducing drug concentrations (Fig. S13B) led to repression of DREAM targets only in MCF7 and RPE1, but not in LN229 cells (Fig. S14). The data suggest that the missing activation of DREAM and the subsequent endoreduplications are specific for glioblastoma cells and independent of the agent.

### Transcriptional silencing of DNA repair is not a hallmark of TMZ-induced senescence

**Fig. 7:**
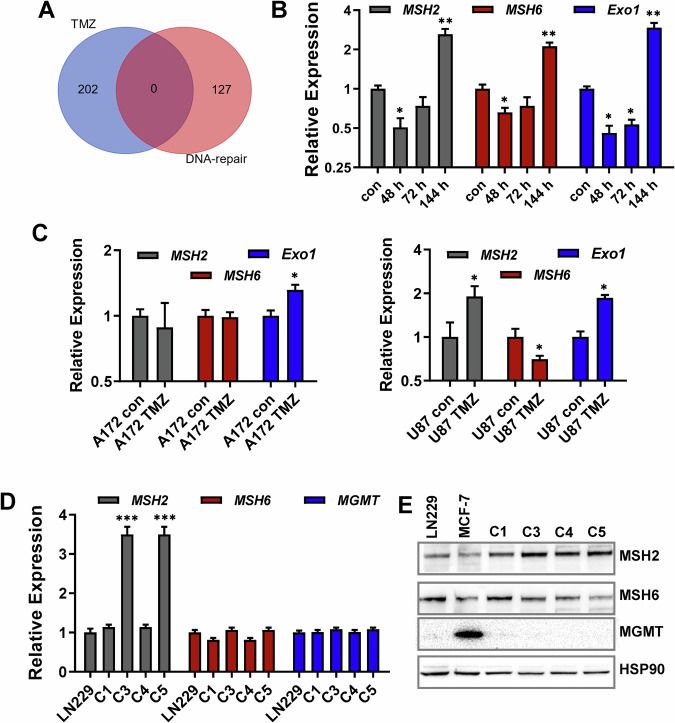
Expression of DNA repair factors upon TMZ exposure. **A** LN229 cells were exposed to 50 µM TMZ for 144 h. RNA was isolated and subjected to RNA-Seq. Number of downregulated DNA repair genes is displayed as Venn diagram. **B** LN229 cells were exposed to 50 µM TMZ for different time periods, and the expression of *MSH2*, *MSH6,* and *EXO1* was analyzed by qPCR. **C** A172 and U87MG cells were exposed to 50 µM TMZ for 144 h, and the expression of *MSH2*, *MSH6,* and *EXO1* was analyzed by qPCR. **B**–**D** Differences between the control and TMZ treatment were statistically analyzed using Student’s *t* test (not-labeled = not significant; *p < 0.1; **p < 0.01; ***p < 0.001. **D** Expression of *MSH2*, *MSH6,* and *MGMT* was analyzed in LN229 cells and four LN229-derived clones escaping/evading TMZ-induced senescence by qPCR. **B**–**D** Experiments were performed in triplicates, *ACTB* and *GAPDH* were used as internal loading control, and the control was set to 1. **E** Expression of MSH2, MSH6, and MGMT was analyzed in LN229 cells and four LN229-derived clones escaping/evading TMZ-induced senescence by immunodetection. HSP90 was used as an internal loading control.

An important hallmark of senescent cells is downregulation of DNA repair genes [37]. However, analyzing the transcriptional response to TMZ in senescent glioblastoma cells revealed that DNA repair genes were not transcriptionally repressed (Fig. 7A). The missing repression was quite surprising, since we previously observed a transcriptional repression of the MMR genes *EXO1, MSH2,* and *MSH6* at early time points upon TMZ treatment [14]. Therefore, we re-analyzed the transcriptional expression of *MSH2*, *MSH6,* and *EXO1* following TMZ. As observed before, the genes were transcriptionally repressed at early times after treatment (48–72 h), however they recovered later when cells reached the senescent state (Fig. 7B). This missing repression at late time points was confirmed in A172 and U87MG cells (Fig. 7C). Of note, nearly all DNA repair genes that are DREAM targets are G1/S-specific (Fig. S15).

To prove whether lack of DREAM is responsible for the re-expression of *MSH2*, *MSH6,* and *EXO1* in the senescence maintenance phase, we inhibited CDK4/6 by palbociclib 48 h after drug treatment. Under these conditions, stable repression of *MSH2, MSH6,* and *EXO1* was achieved (Fig. S12). Obviously, the initial transcriptional repression of *MSH2, MSH6,* and *EXO1* is lost during senescence maintenance. Decreased expression of MMR factors was described in glioblastoma relapses [38, 39]. Therefore, the question arose as to whether re-expression of these genes occurs in cells that were regrown from the senescent population. To address this, we generated a large number of cell clones that grew up as survivors from a TMZ-exposed cell population. The frequency of these clones was 0.02% of the senescent cell population. In none of them transcriptional repression of *MSH2* or *MSH6* was observed (Figs. 7D and S16), indicating that alterations in MMR activity in recurrences are not directly associated with senescence evasion. Same results were obtained on protein level (Fig. 7E). Since MGMT is a most important determinant of TMZ resistance, we also analyzed a potential change in MGMT expression, however no reactivation of MGMT was observed (Figs. 7D, E and S16).

## Discussion

Here we show that the methylating anticancer drug TMZ strongly activates p21^CIP1^-dependent cellular senescence in glioblastoma cells, which is independent of DREAM activation. Our data revealed that p21^CIP1^ does not interact with CDK4, and the DREAM complex is not formed. As a consequence, only a subset of potential DREAM targets was repressed, namely G2/M-specific genes. In contrast, G1/S-specific DREAM targets (notably *E2F1* and *MYBL2*) were not repressed. The findings are opposed to the transcriptional response we observed following B[a]P and IR in breast cancer cells, which led to DREAM formation and repression of G1/S and G2/M-specific DREAM-regulated genes [40].

In the TMZ-triggered O^6^-methylguanine response, futile mismatch repair on O^6^MeG/T mismatches causes gaps and subsequent replication fork collapse leading to the formation of DSBs [41, 42]. As shown here, this results in inactivation of CDK1 by two mechanisms: (i) Activated CHK1 phosphorylates and thus activates WEE1, which acts as a direct CDK1 inhibitor, and (ii) CHK1 phosphorylates CDC25c resulting in its proteasomal degradation. Inactivation of CDK1 leads to missing phosphorylation/activation of B-Myb and thereby repression of B-Myb targets, including *CCNB2*, *PLK1,* and *FOXM1*. At later times, p21^CIP1^ inhibits CDK1 and CDK2, enhancing the transcriptional repression of B-Myb and FOXM1 targets and thus arresting the cells in the G2-phase. However, the suppression of B-Myb and FOXM1 activity seems not to be sufficient and, therefore, the G2/M-arrest is leaky, allowing E2F1-mediated restart of DNA synthesis, resulting in endoreduplications and polyploidy. Of note, the CDK4/6 inhibitor palbociclib, which is clinically used in adjuvant cancer therapy, reduced the expression of *E2F1, MYBL2,* and *FOXM1* and of endoreduplications. The DREAM response was also not observed in GBM cells after irinotecan and oxaliplatin treatment. On opposite, MCF7 and RPE1 cells responded with a full DREAM response, indicating that the missing DREAM response is specific for glioma cells treated with genotoxic drugs.

Our data show that p21^Cip1^ is not essential for the activation of the G2/M-arrest, since the G2/M-arrest occurred in both p53-proficient and p53-deficient cells (not upregulating p21^CIP1^) following TMZ treatment and was abrogated upon inhibition of CHK1 [14, 43, 44]. Overall, our data suggest that p21^Cip1^ is involved in turning the G2/M-arrest irreversible, which seems to be a bona fide prerequisite for the onset and maintenance of senescence. This and the question of why p21^CIP1^ does not interact with CDK4 in GBM cells are important topics to be addressed in future research.

Among the DREAM targets, DNA repair genes play an important role. Thus, downregulation of DNA repair triggered by DREAM is thought to be a hallmark of senescent cells [37, 45]. Actually, in GBM cells, early after TMZ exposure, we observed repression of DNA repair genes which, however, recovered in the senescent population. This is explained by the finding that *MSH2*, *MSH6,* and *EXO1* as well as many other DNA repair genes are G1/S-specific targets of DREAM, which was not fully activated. It seems that TMZ induces a specific senescence phenotype in GBM cells that is in sharp contrast to mammary cancer cells and non-transformed cells following genotoxic stress [40]. Overall, our data support the notion that downregulation of DNA repair genes is a hallmark of DREAM-mediated senescence, which appears to be tumor-specific.

What are the clinical implications? We would like to propose that the unstable G2/M-arrest and endoreduplications observed following TMZ exposure, causing the formation of giant cells might contribute to therapy resistance. Genotoxin-induced polyploid giant cells have been observed in other studies. Thus, using a set of four human cancer cell lines, a direct relation between invasiveness and polyploidy was observed [46]. Importantly, it has been suggested that polyploidy may confer cells the ability to escape from senescence. Therefore, killing of polyploid cells is anticipated to improve anticancer treatment (for review see [47]). In line with this, our data suggest that CDK4/6 inhibitors, which are already in the clinic [48, 49], might foster TMZ-induced senescence *via* additional inhibition of the E2F1 pathway and thereby prevent endoreduplications and the formation of therapy-refractory polyploid cells.

## Supplementary information

Supplementary figures

Supplementary figure legends

Uncropped western blots

## Data Availability

Raw transcriptomics data are available at GEO (Gene expression omnibus) under GSE276693.
