# Supplementary material for: Therapy-induced senescence of glioblastoma cells is determined by the p21CIP1-CDK1/2 axis and does not require activation of DREAM
Source: Cell Death Dis. 2025 May 3;16(1):357. doi: 10.1038/s41419-025-07651-8 (PMC12049523; doi:10.1038/s41419-025-07651-8)
Supplement: Supplementary file 1 — Supplementary figures [file 41419_2025_7651_MOESM1_ESM.pdf]

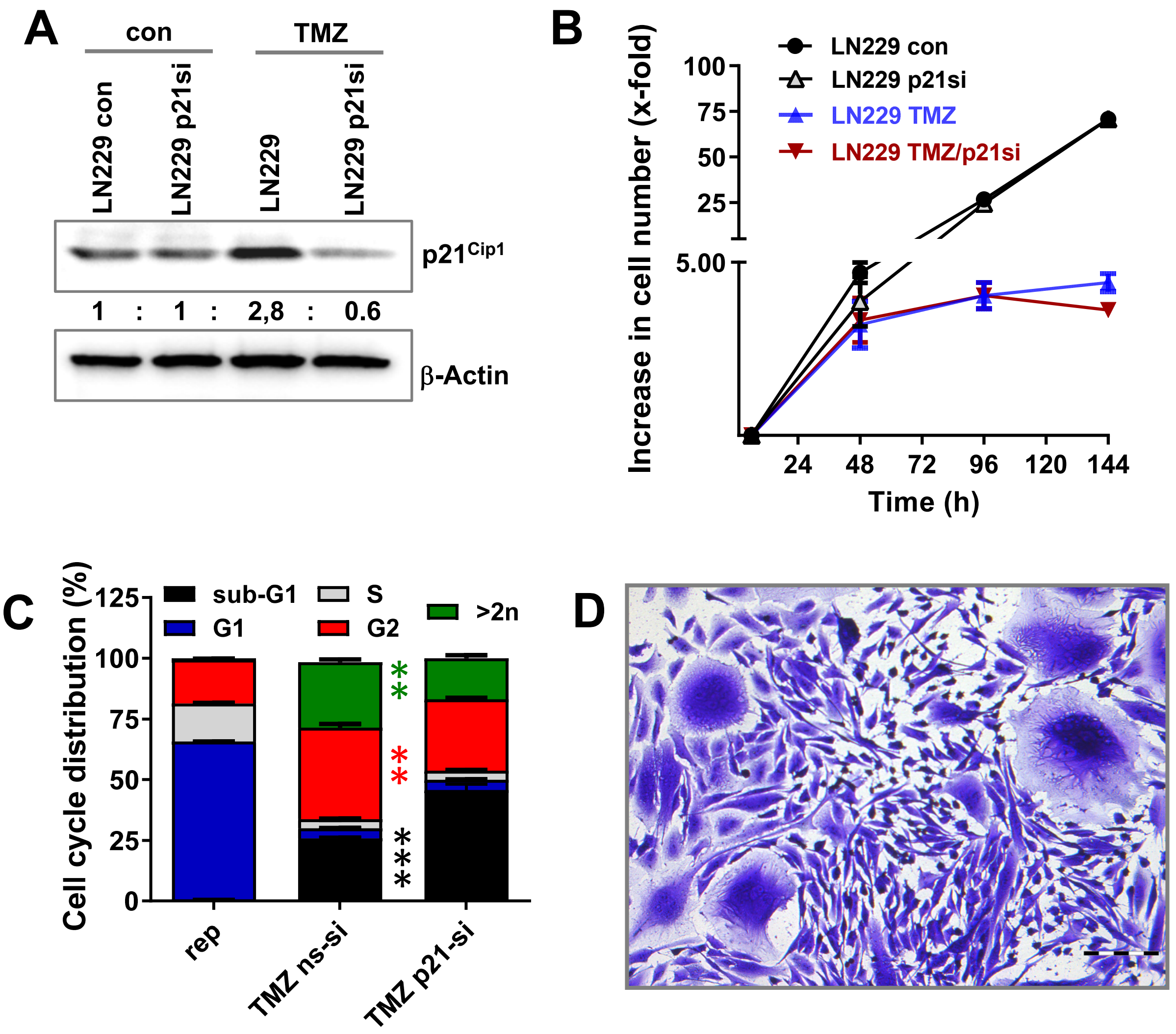
A
con
TMZ
LN229 con
LN229 p21si
LN229
LN229 p21si
p21Cip1
1 : 1 : 2,8 : 0.6
β-Actin
B
LN229 con
LN229 p21si
LN229 TMZ
LN229 TMZ/p21si
Increase in cell number (x-fold)
100
75
50
25
5.00
24
48
72
96
120
144
Time (h)
C
sub-G1
S
>2n
G1
G2
Cell cycle distribution (%)
125
100
75
50
25
0
rep
TMZ ns-si
TMZ p21-si
D

Figure S1

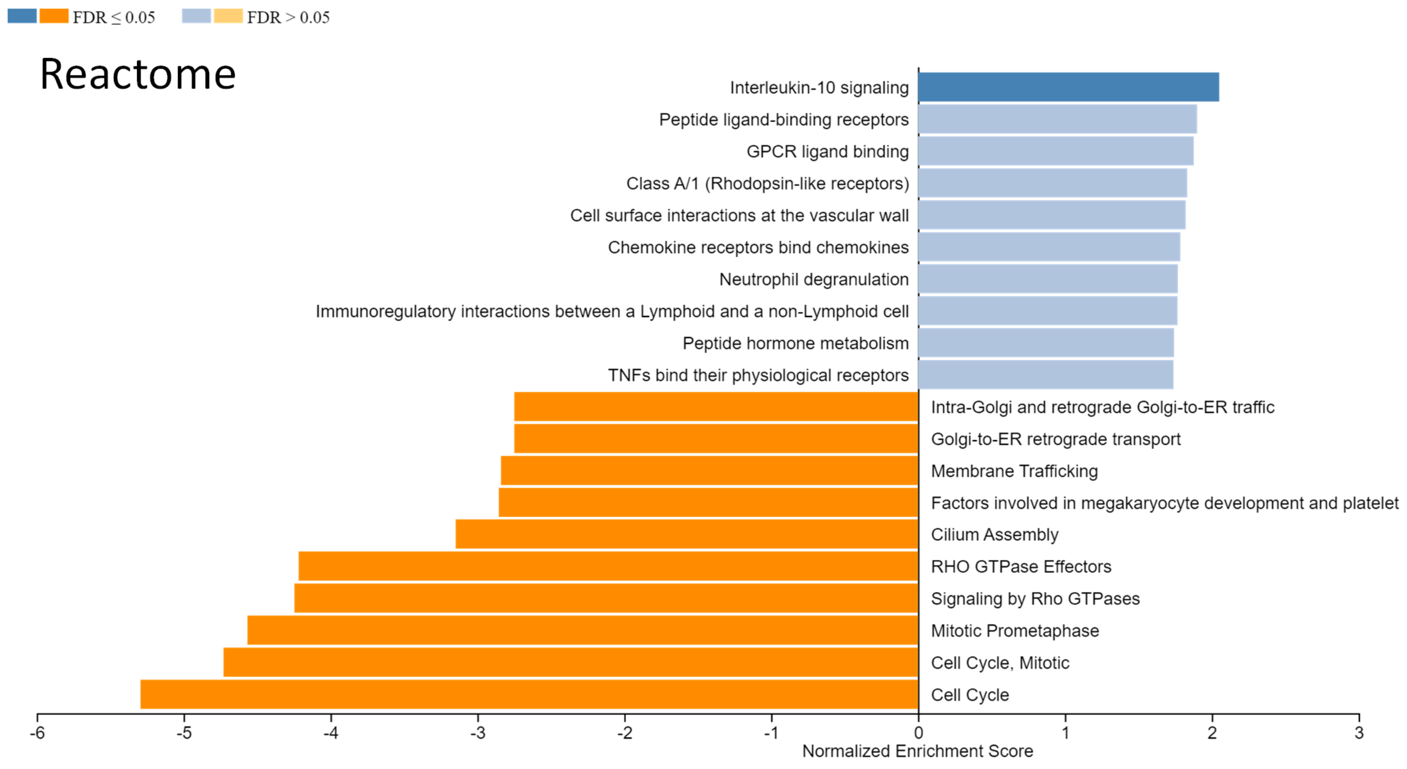
FDR ≤ 0.05
FDR > 0.05
Reactome
Interleukin-10 signaling
Peptide ligand-binding receptors
GPCR ligand binding
Class A/1 (Rhodopsin-like receptors)
Cell surface interactions at the vascular wall
Chemokine receptors bind chemokines
Neutrophil degranulation
Immunoregulatory interactions between a Lymphoid and a non-Lymphoid cell
Peptide hormone metabolism
TNFs bind their physiological receptors
Intra-Golgi and retrograde Golgi-to-ER traffic
Golgi-to-ER retrograde transport
Membrane Trafficking
Factors involved in megakaryocyte development and platelet
Cilium Assembly
RHO GTPase Effectors
Signaling by Rho GTPases
Mitotic Prometaphase
Cell Cycle, Mitotic
Cell Cycle
-6
-5
-4
-3
-2
-1
0
1
2
3
Normalized Enrichment Score

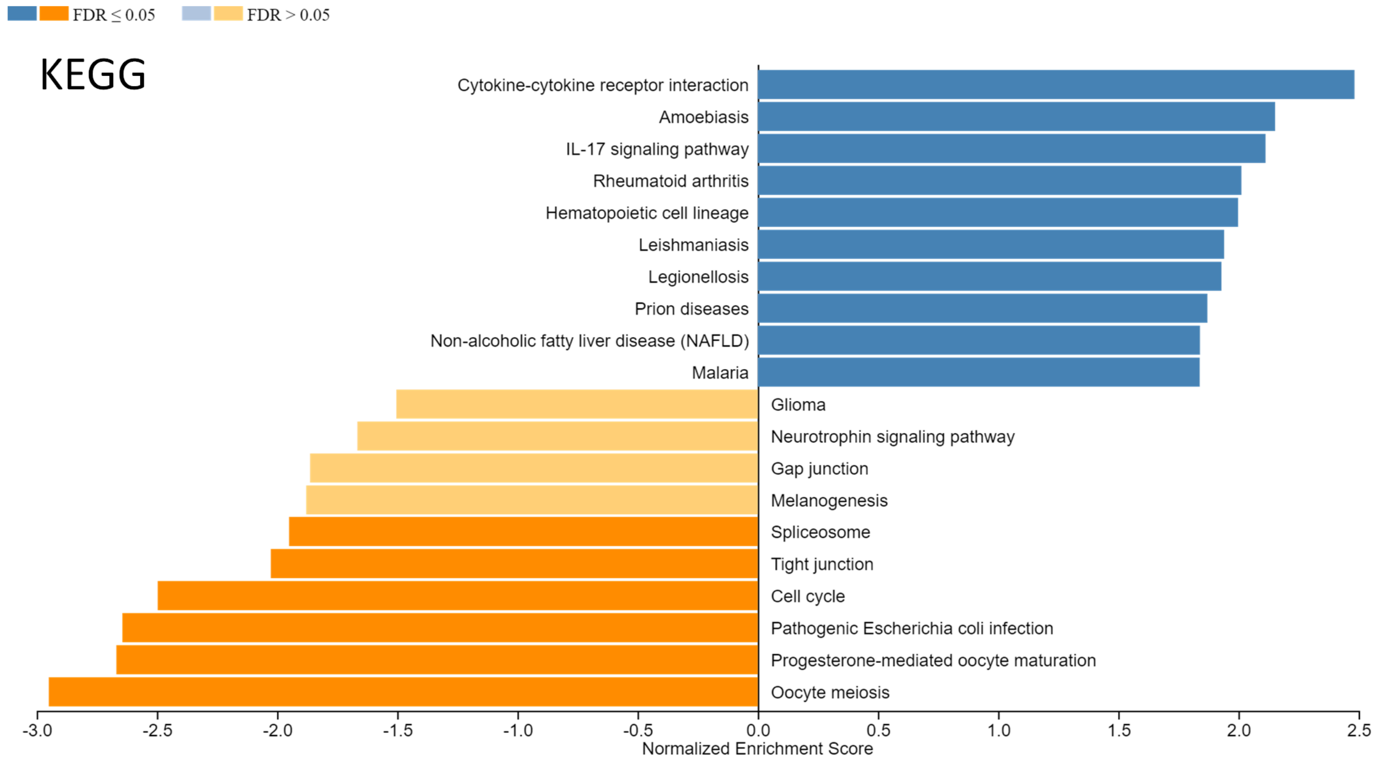
FDR ≤ 0.05
FDR > 0.05
KEGG
Cytokine-cytokine receptor interaction
Amoebiasis
IL-17 signaling pathway
Rheumatoid arthritis
Hematopoietic cell lineage
Leishmaniasis
Legionellosis
Prion diseases
Non-alcoholic fatty liver disease (NAFLD)
Malaria
Glioma
Neurotrophin signaling pathway
Gap junction
Melanogenesis
Spliceosome
Tight junction
Cell cycle
Pathogenic Escherichia coli infection
Progesterone-mediated oocyte maturation
Oocyte meiosis
-3.0
-2.5
-2.0
-1.5
-1.0
-0.5
0.0
0.5
1.0
1.5
2.0
2.5
Normalized Enrichment Score

**Figure S2**

**A**

| TMZ-48h-p53-up | TMZ-48h/144h-p53-up | TMZ-144h-p53-up |
|---|---|---|
| BHLHE40 GADD45A PSTPIP2 SERPINB5 | ABCA12 ACTA2 ATF3 BBC3 BTG2 CDKN1A CYFIP2T DGKA EDA2R GDF15 GREB1 IGDCC4 MDM2 NUPR1 RGS16 RRM2B SESN2 TP53INP1 TRIM22 TSPAN11 | APOBEC3H APOD BTG1 CD82 CLCA2 COQ8A DRAM1 EPS8L2 ETV7 F5 IGFBP7 LRP1 MFGE8 MUC2 ORAI3 PML POU2F2 SCN2A SERPINE1 TLR3 TNFRSF10B TXNIP VWCE ZMAT3 |

**B**

| TMZ-48h-DREAM-F -down | TMZ-48h/144h-DREAM-F-down | TMZ-144h-DREAM-F-down |
|---|---|---|
| ARHGEF39 | ARHGAP11A ARL6IP1 AURKA BIRC5 BUB1 CCNA2 CCNB1 CCNF CDC20 CDCA2 CDCA3 CDCA8 CENPA DEPDC1 DEPDC1B DLGAP5 FAM83D GTSE1 HJURP HMGB3 HMMR HSP90AA1 KIF20A KIF23 KIFC1 KNSTRN KPNA2 LMNB1 NEK2 PHF19 PIF1 PIMREG PLK1 PSRC1 SAPCD2 SPAG5 TPX2 TROAP | ANLN ANP32E AURKB BORA CCDC34 CCNB2 CDKN3 CEP55 CKAP2 CKS2 DBF4 DDX39A FAM110A FOXM1 GINS2 HMGB1 HMGB2 HMGN2 HNRNPA1 HNRNPA2B1 HYLS1 JPT1 KIF11 KIF18B KIF2C NCAPH NUF2 PBK PRC1 RACGAP1 RCC1 SKA3 SPC25 SPDL1 TUBA1C TUBB UBE2C UBE2S ZWINT |

| TMZ-48h-DREAM-E -down | TMZ-48h/144h-DREAM-E-down | TMZ-144h-DREAM-E-down |
|---|---|---|
| ARHGEF39 | ARHGAP11A ARL6IP1 AURKA BIRC5 BUB1 CCNB1 CDC20 CDCA2 CDCA3 CDCA8 CENPA DEPDC1 DEPDC1B FAM83D GTSE1 HJURP HMMR KIF23 KIFC1 KNSTRN KPNA2 LMNB1 NEK2 PHF19 PIF1 PLK1 PSRC1 SPAG5 TPX2 | AURKB ANLN ANP32E BORA CCDC34 CCNB2 CDKN3 CEP55 CKAP2 CKS2 FOXM1 GINS2 HMGB2 HNRNPA2B1 KIF11 KIF2C NCAPH NUF2 PRC1 RACGAP1 SPC25 SPDL1 UBE2C UBE2S |

| TMZ-48h-DREAM-U -down | TMZ-48h/144h-DREAM-U-down | TMZ-144h-DREAM-U-down |
|---|---|---|
| ARHGEF39 | ARHGAP11A AURKA BIRC5 BUB1 CCNA2 CCNB1 CDC20 CDCA2 CDCA3 CDCA8 CENPA DEPDC1 DEPDC1B DLGAP5 FAM83D GTSE1 HJURP HMMR KIF20A KIF23 KIFC1 KNSTRN NEK2 PIF1 PIMREG PLK1 PSRC1 SPAG5 TPX2 TROAP | AURKB ANLN BORA CCNB2 CEP55 CKS2 FAM72B FOXM1 GINS2 HMGB1 HMGB2 KIF11 KIF18B KIF2C NCAPH NUF2 PBK PRC1 RACGAP1 SKA3 SLC25A10 SPC25 SPDL1 UBE2C ZWINT |

**Figure S3**

**A**

| TMZ-48h-G1/S down | TMZ-48h/144h-G1/S down | TMZ-144h-G1/S down |
|---|---|---|
| | - | CCN1 CCN2 GINS2[1] MAP2K6 RGS7 |
| **TMZ-48h-G2/M down** | **TMZ-48h/144h-G2/M down** | **TMZ-144h-G2/M down** |
| ARHGEF39[1] | ARHGAP11A[1] ARL6IP1[1] AURKA[1] BIRC5[1] BUB1[1] CCNA2[1] CCNB1[1] CCNF[1] CDC20[1] CDCA2[1] CDCA3[1] CDCA8[1] CENPA[1] DEPDC1[1] DEPDC1B[1] FAM83D[1] GTSE1[1] HJURP[1] HMGB3[1] HMMR[1] KIF20A[1] KIF23[1] KIFC1[1] KNSTRN[1] KPNA2[1] NEK2[1] PIF1[1] PIMREG[1] PLK1[1] PSRC1[1] SAPCD2[1] SPAG5[1] TNFAIP8L1 TPX2[1] TROAP[1] | ANLN[1] ANP32E[1] AURKB[1] CCNB2[1] CEP55[1] CKAP2[1] CKS2[1] DBF4[1] FAM110A[1] FOXM1[1] HMGB2[1] JPT1[1] KIF11[1] KIF18B[1] KIF2C[1] NCAPH[1] NUF2[1] PBK[1] PRC1[1] RACGAP1[1] TUBB[1] UBE2C[1] UBE2S[1] |

[1] ***also DREAM-F targets***

**B**

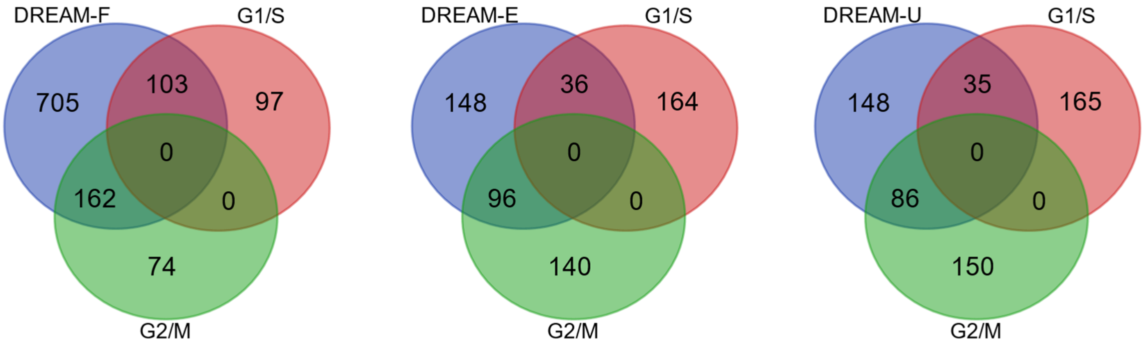


**C**

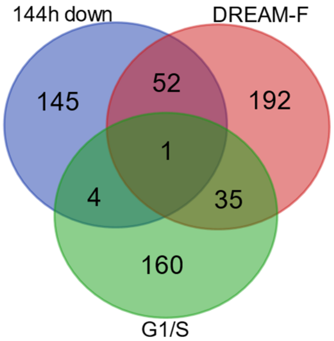


| TMZ-144h-G1/S down DREAM-F | TMZ-144h-G1/S down |
|---|---|
| GINS2 | CCN1 CCN2 MAP2K6 RGS7 |

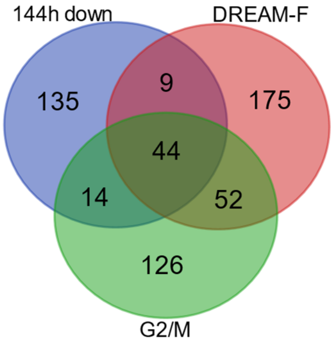


| TMZ-144h-G2/M down DREAM-F | TMZ-144h-G2/M down |
|---|---|
| ANLN ANP32E ARHGAP11A ARL6IP1 AURKA AURKB BIRC5 BUB1 CCNB1 CCNB2 CDC20 CDCA2 CDCA3 CDCA8 CENPA CEP55 CKAP2 CKS2 DEPDC1 DEPDC1B FAM83D FOXM1 GTSE1 HJURP HMGB2 HMMR KIF11 KIF23 KIF2C KIFC1 KNSTRN KPNA2 NCAPH NEK2 NUF2 PIF1 PLK1 PRC1 PSRC1 RACGAP1 SPAG5 TPX2 UBE2C UBE2S | CCNA2 CCNF DBF4 FAM110A HMGB3 JPT1 KIF18B KIF20A PBK PIMREG SAPCD2 TNFAIP8L1 TROAP TUBB |

**Figure S4**

**A**

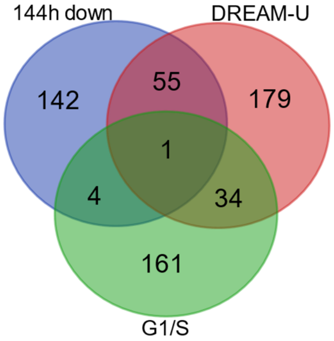


| TMZ-144h-G1/S down DREAM-U | TMZ-144h-G1/S down |
| --- | --- |
| GINS2 | CCN1 CCN2 MAP2K6 RGS7 |

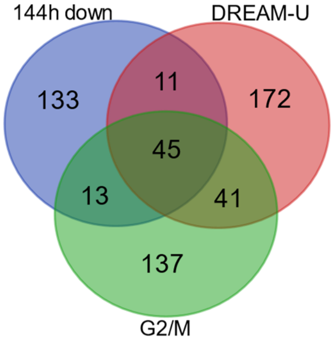


| TMZ-144h-G2/M down DREAM-U | TMZ-144h-G2/M down |
| --- | --- |
| ANLN ARHGAP11A AURKA AURKB BIRC5 BUB1 CCNA2 CCNB1 CCNB2 CDC20 CDCA2 CDCA3 CDCA8 CENPA CEP55 CKS2 DEPDC1 DEPDC1B FAM83D FOXM1 GTSE1 HJURP HMGB2 HMMR KIF11 KIF18B KIF20A KIF23 KIF2C KIFC1 KNSTRN NCAPH NEK2 NUF2 PBK PIF1 PIMREG PLK1 PRC1 PSRC1 RACGAP1 SPAG5 TPX2 TROAP UBE2C | ANP32E ARL6IP1 CCNF CKAP2 DBF4 FAM110A HMGB3 JPT1 KPNA2 SAPCD2 TNFAIP8L1 TUBB UBE2S |

**B**

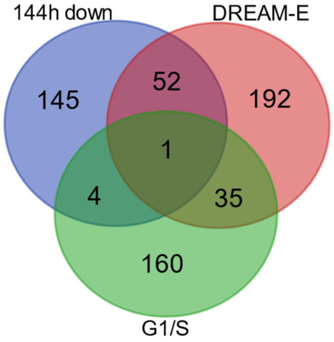


| TMZ-144h-G1/S down DREAM-E | TMZ-144h-G1/S down |
| --- | --- |
| GINS2 | CCN1 CCN2 MAP2K6 RGS7 |

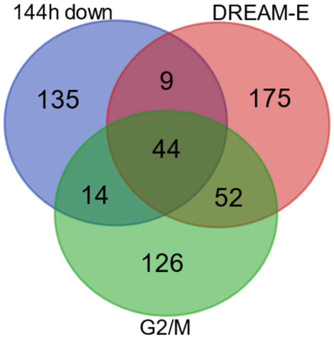


| TMZ-144h-G2/M down DREAM-E | TMZ-144h-G2/M down |
| --- | --- |
| ANLN ANP32E ARHGAP11A ARL6IP1 AURKA AURKB BIRC5 BUB1 CCNB1 CCNB2 CDC20 CDCA2 CDCA3 CDCA8 CENPA CEP55 CKAP2 CKS2 DEPDC1 DEPDC1B FAM83D FOXM1 GTSE1 HJURP HMGB2 HMMR KIF11 KIF23 KIF2C KIFC1 KNSTRN KPNA2 NCAPH NEK2 NUF2 PIF1 PLK1 PRC1 PSRC1 RACGAP1 SPAG5 TPX2 UBE2C UBE2S | CCNA2 CCNF DBF4 FAM110A HMGB3 JPT1 KIF18B KIF20A PBK PIMREG SAPCD2 TNFAIP8L1 TROAP TUBB |

**Figure S5**

| TMZ-48h-MCO down | TMZ-48h/144h-MCO down | TMZ-144h-MCO down |
|---|---|---|
| - | AURKA CCNB1 CDC20 CENPA DLGAP5 KIF23 KIFC1 NEK2 PLK1 PSRC1 SAPCD2 TPX2 | BORA KIF11 NUF2 PRC1 RACGAP1 RAN RCC1 SPC25 SPDL1 URKB |
| **TMZ-48h-MND down** | **TMZ-48h/144h-MND down** | **TMZ-144h-MND down** |
| - | AURKA BUB1 CCNB1 CDC20 CDCA2 CDCA8 DLGAP5 KIF23 KIFC1 KNSTRN NEK2 PLK1 PSRC1 SPAG5 TPX2 | ANAPC15 ANLN AURKB BORA CAV2 CEP55 KIF11 KIF18B KIF2C NCAPH NUF2 PRC1 RACGAP1 RAN RCC1 SPDL1 UBE2C UBE2S ZWINT |

# Figure S6

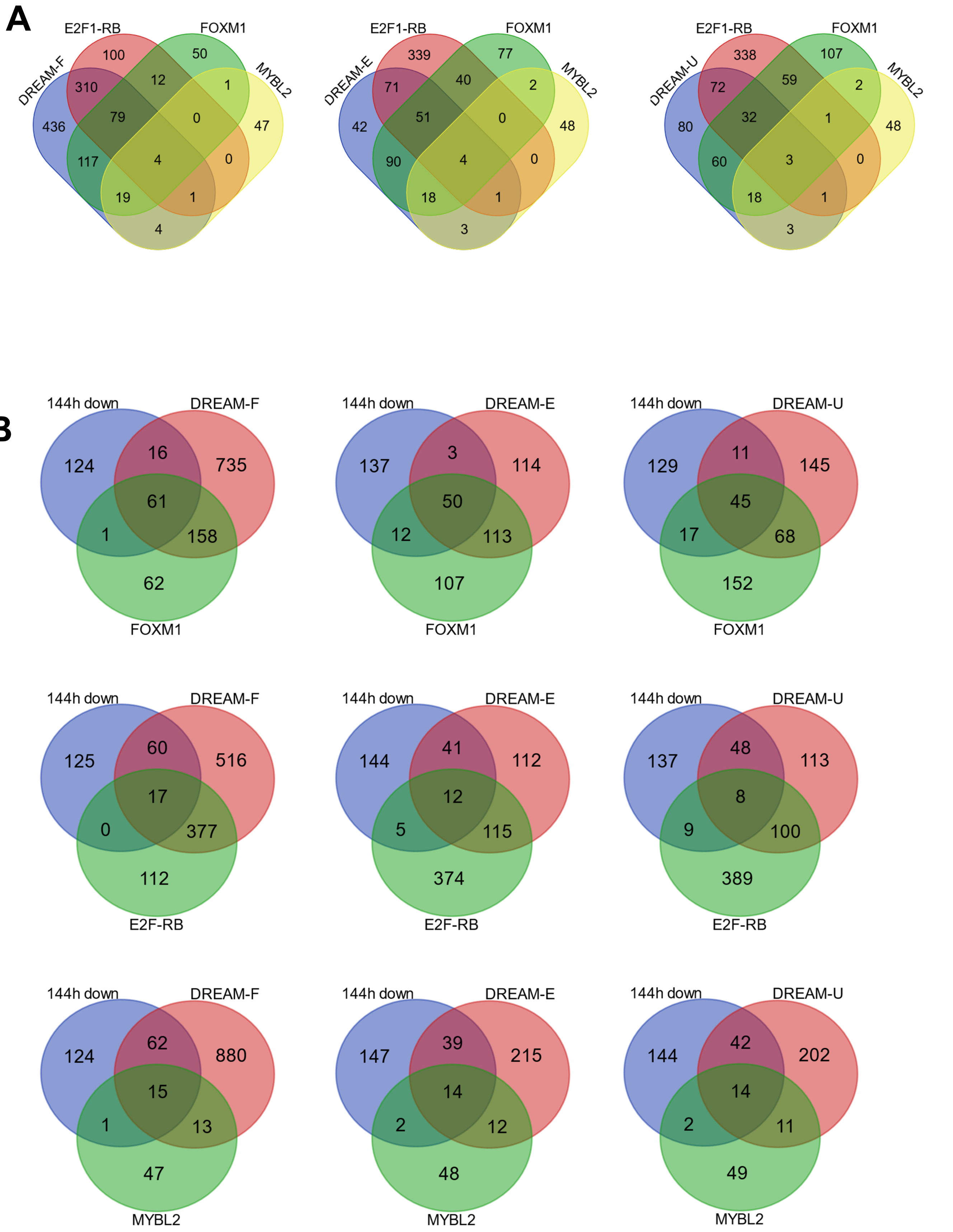
A
E2F1-RB
FOXM1
DREAM-F
MYBL2
100
50
310
12
1
436
79
0
47
117
4
0
19
1
4
E2F1-RB
FOXM1
DREAM-E
MYBL2
339
77
71
40
2
42
51
0
48
90
4
0
18
1
3
E2F1-RB
FOXM1
DREAM-U
MYBL2
338
107
72
59
2
80
32
1
48
60
3
0
18
1
3
B
144h down
DREAM-F
124
16
735
61
1
158
62
FOXM1
144h down
DREAM-E
137
3
114
50
12
113
107
FOXM1
144h down
DREAM-U
129
11
145
45
17
68
152
FOXM1
144h down
DREAM-F
125
60
516
17
0
377
112
E2F-RB
144h down
DREAM-E
144
41
112
12
5
115
374
E2F-RB
144h down
DREAM-U
137
48
113
8
9
100
389
E2F-RB
144h down
DREAM-F
124
62
880
15
1
13
47
MYBL2
144h down
DREAM-E
147
39
215
14
2
12
48
MYBL2
144h down
DREAM-U
144
42
202
14
2
11
49
MYBL2

**Figure S7**

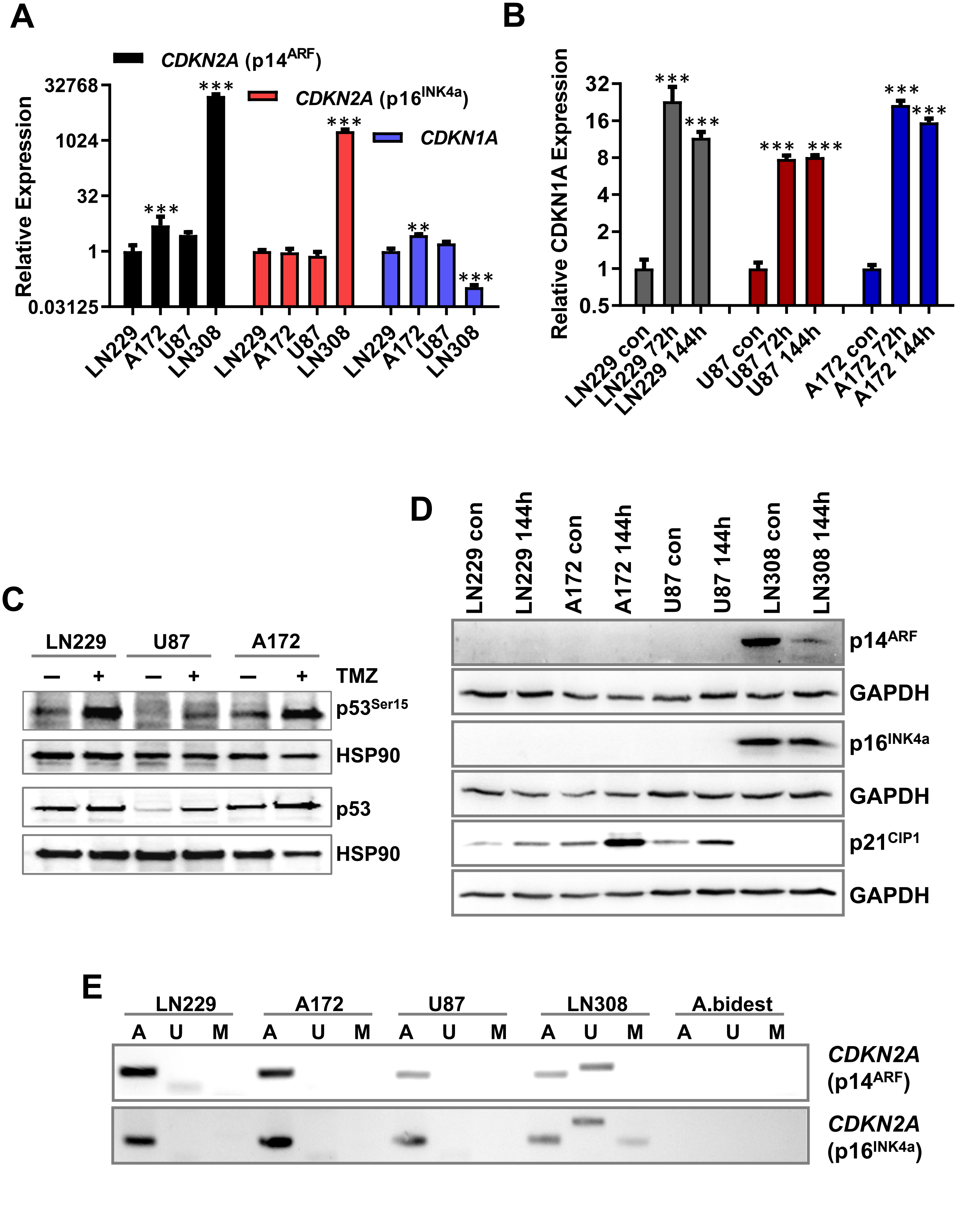
A
CDKN2A (p14ARF)
CDKN2A (p16INK4a)
CDKN1A
Relative Expression
32768
1024
32
1
0.03125
LN229
A172
U87
LN308
B
Relative CDKN1A Expression
LN229 con
LN229 72h
LN229 144h
U87 con
U87 72h
U87 144h
A172 con
A172 72h
A172 144h
C
LN229
U87
A172
TMZ
p53Ser15
HSP90
p53
D
LN229 con
LN229 144h
A172 con
A172 144h
U87 con
U87 144h
LN308 con
LN308 144h
p14ARF
GAPDH
p16INK4a
p21CIP1
E
A.bidest
A U M
CDKN2A (p14ARF)
CDKN2A (p16INK4a)

Figure S8

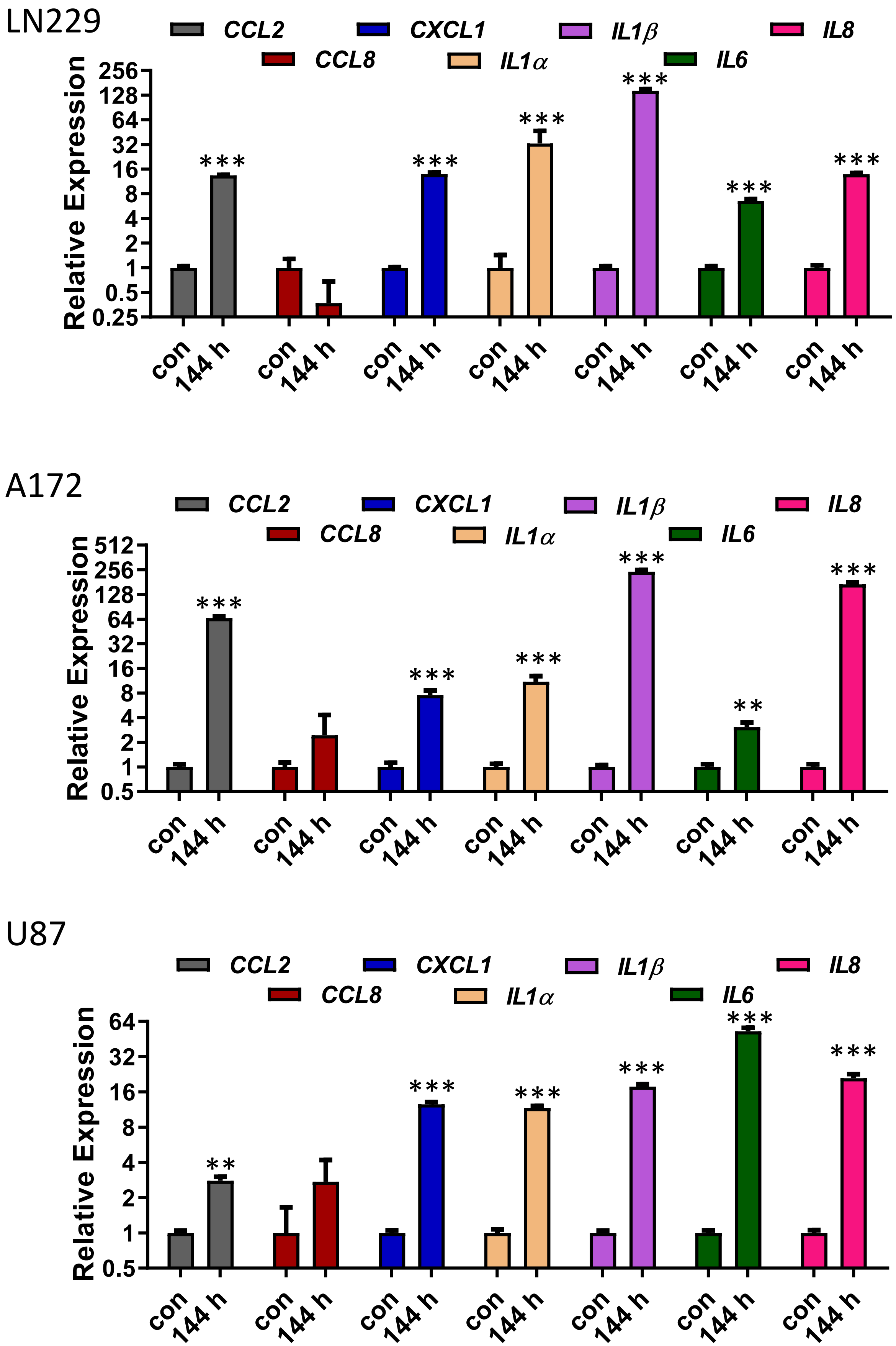
LN229
CCL2
CXCL1
IL1β
IL8
CCL8
IL1α
IL6
Relative Expression
256
128
64
32
16
8
4
2
1
0.5
0.25
***
con
144 h
A172
512
Relative Expression
**
U87
64
32
16
8
4
2
1
0.5

**Figure S9**

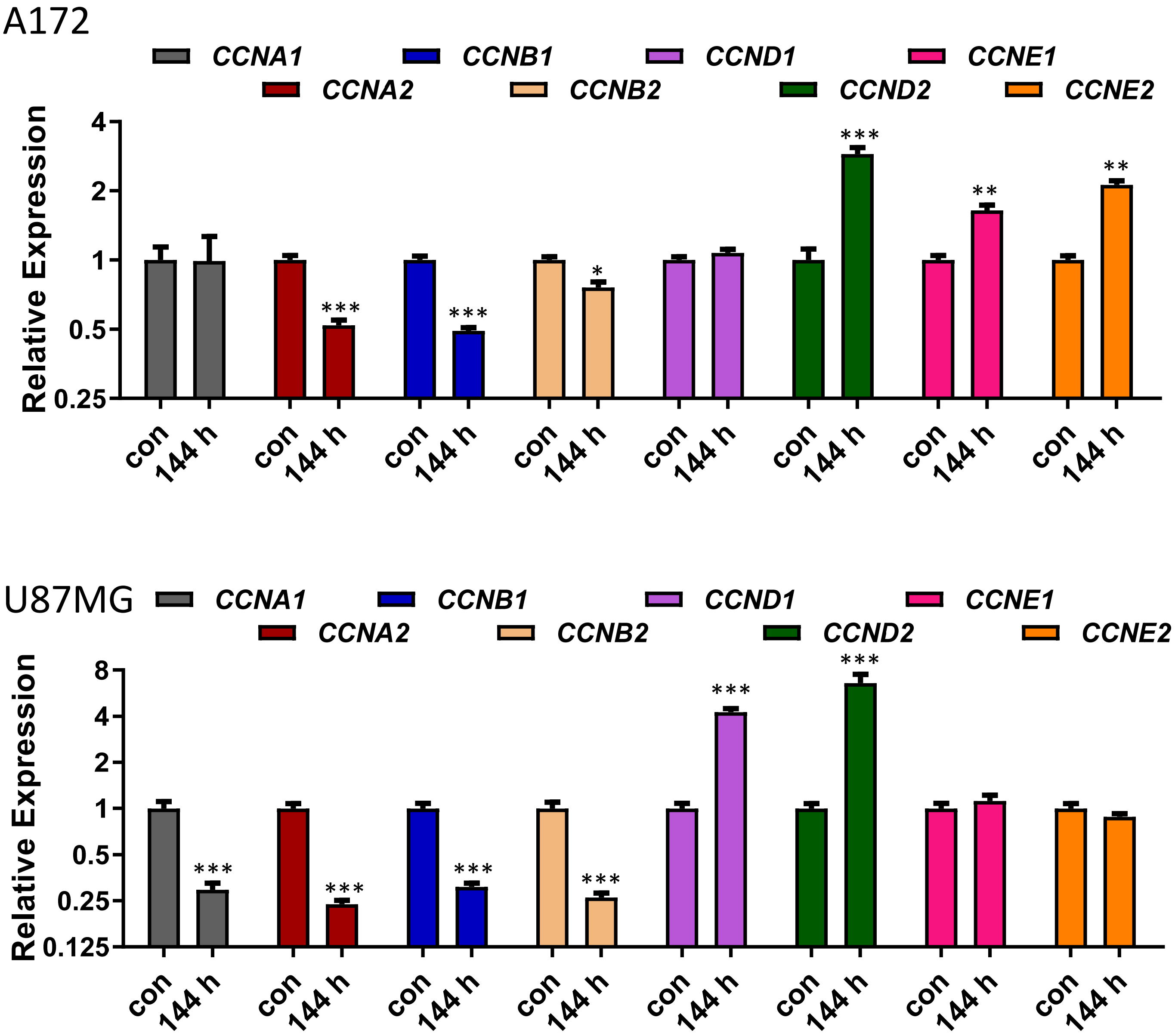

A172
CCNA1
CCNB1
CCND1
CCNE1
CCNA2
CCNB2
CCND2
CCNE2
Relative Expression
4
2
1
0.5
0.25
***
**
**
***
***
*
con
144 h
U87MG
CCNA1
CCNB1
CCND1
CCNE1
CCNA2
CCNB2
CCND2
CCNE2
Relative Expression
8
4
2
1
0.5
0.25
0.125
***
***
***
***
***
***
***
con
144 h


**Figure S10**

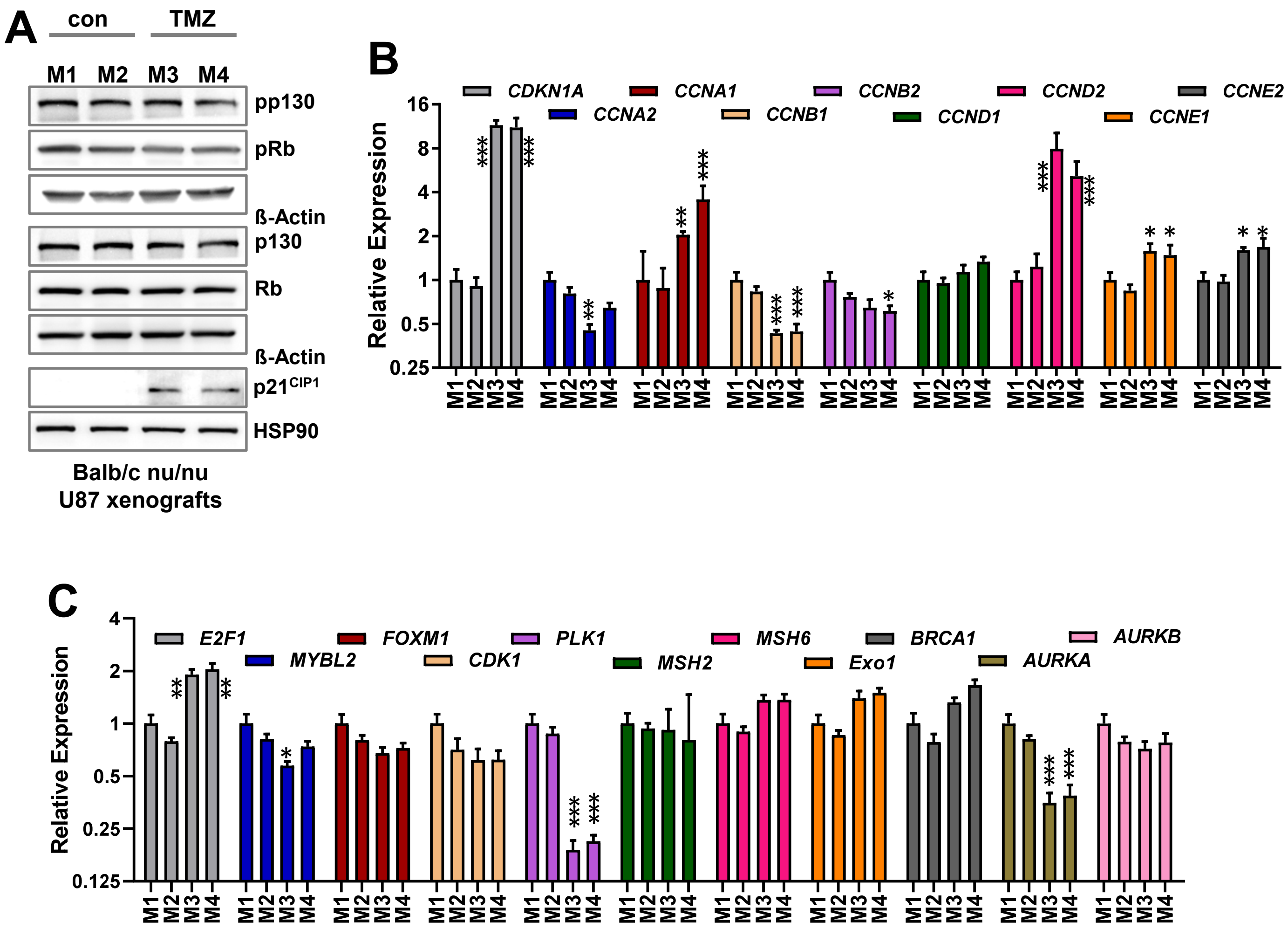
A
con
TMZ
M1 M2 M3 M4
pp130
pRb
ß-Actin
p130
Rb
ß-Actin
p21CIP1
HSP90
Balb/c nu/nu
U87 xenografts
B
CDKN1A
CCNA2
CCNA1
CCNB1
CCNB2
CCND1
CCND2
CCNE1
CCNE2
Relative Expression
16
8
4
2
1
0.5
0.25
M1 M2 M3 M4
C
E2F1
MYBL2
FOXM1
CDK1
PLK1
MSH2
MSH6
Exo1
BRCA1
AURKA
AURKB
Relative Expression
4
2
1
0.5
0.25
0.125
M1 M2 M3 M4

Figure S11

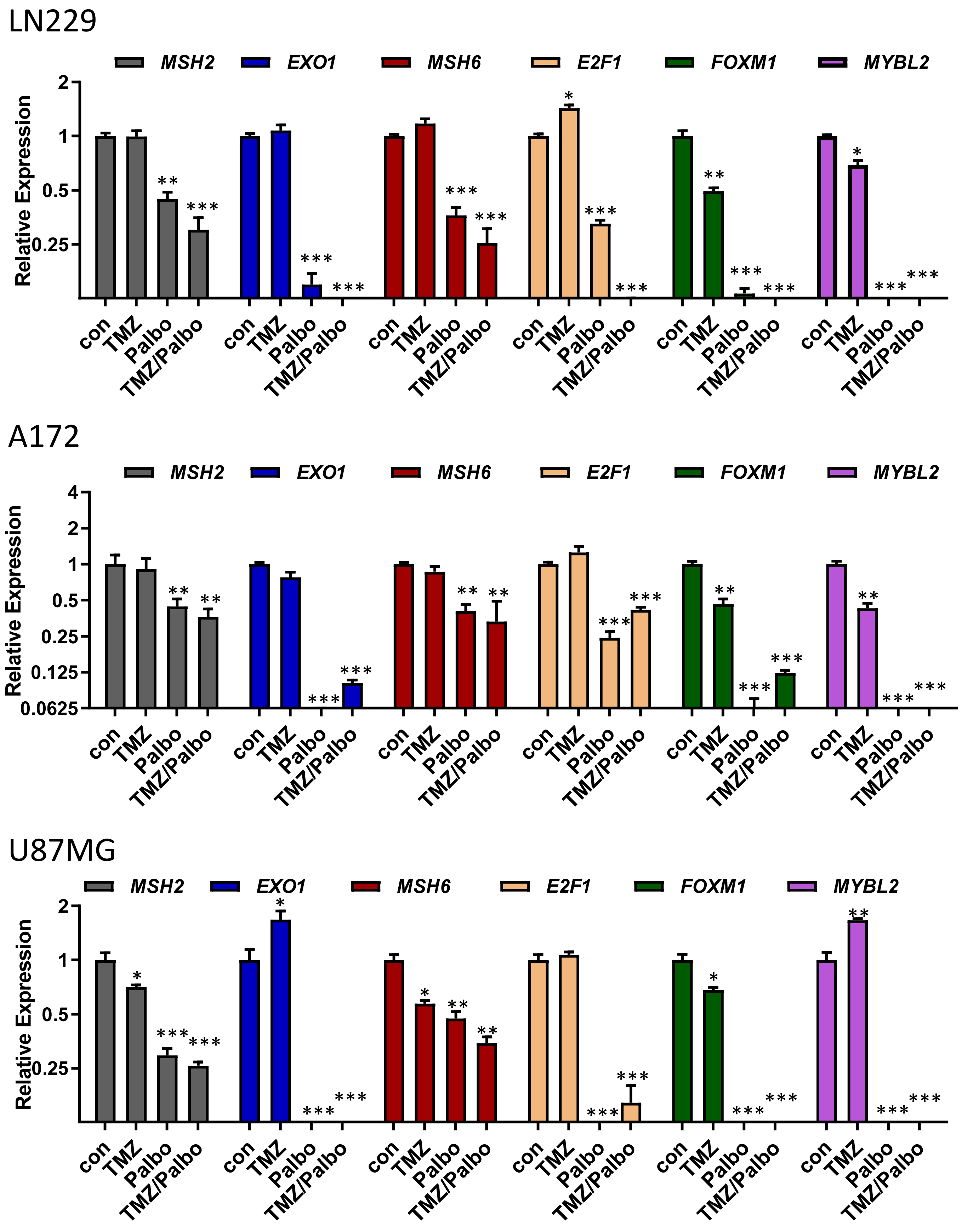
LN229
MSH2
EXO1
MSH6
E2F1
FOXM1
MYBL2
Relative Expression
2
1
0.5
0.25
con
TMZ
Palbo
TMZ/Palbo
A172
MSH2
EXO1
MSH6
E2F1
FOXM1
MYBL2
Relative Expression
4
2
1
0.5
0.25
0.125
0.0625
con
TMZ
Palbo
TMZ/Palbo
U87MG
MSH2
EXO1
MSH6
E2F1
FOXM1
MYBL2
Relative Expression
2
1
0.5
0.25
con
TMZ
Palbo
TMZ/Palbo

**Figure S12**

A

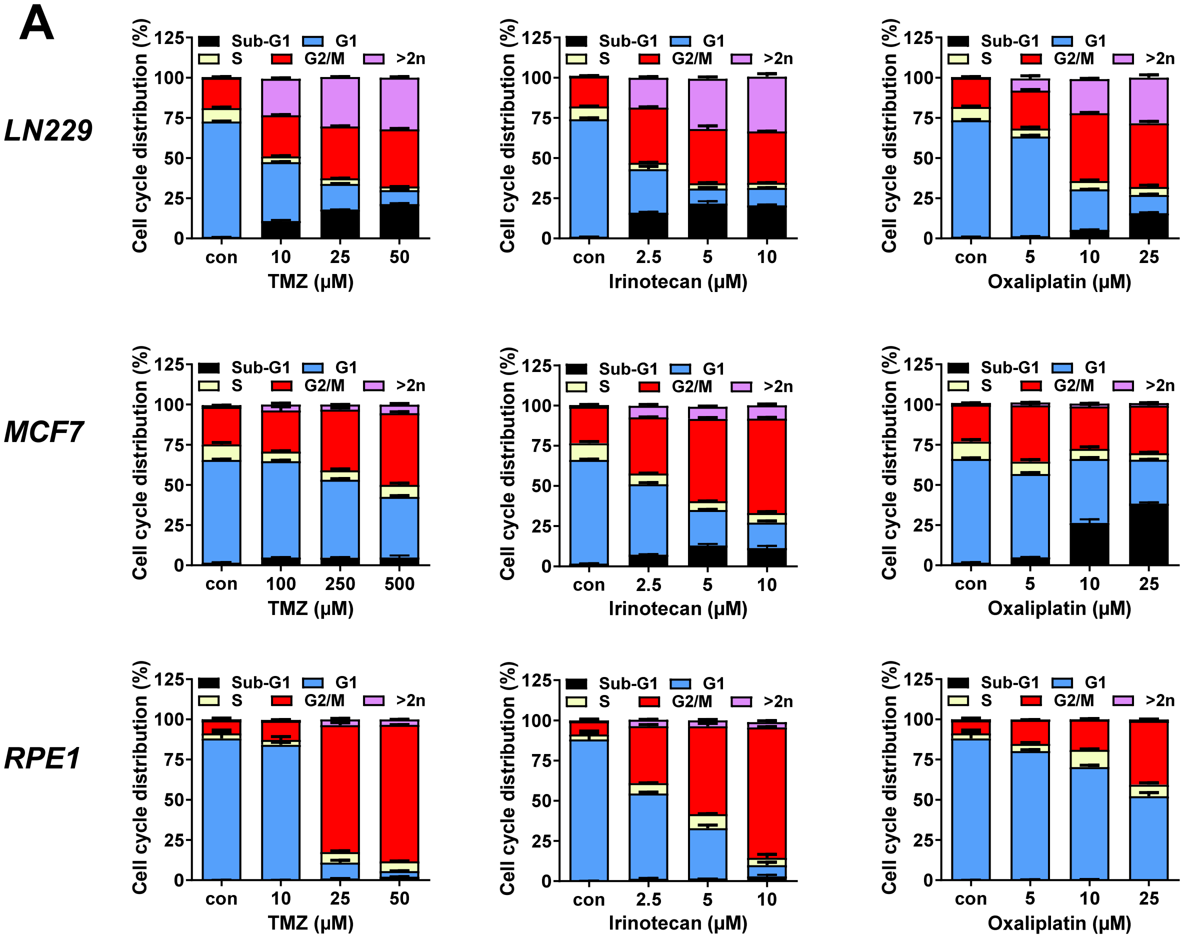


B

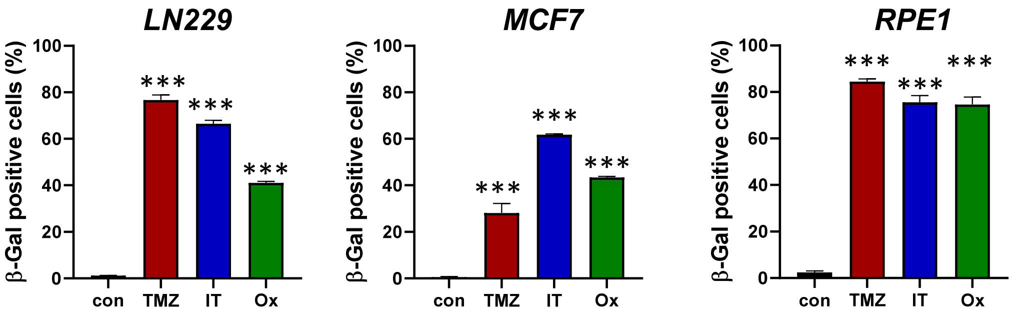


Figure S13

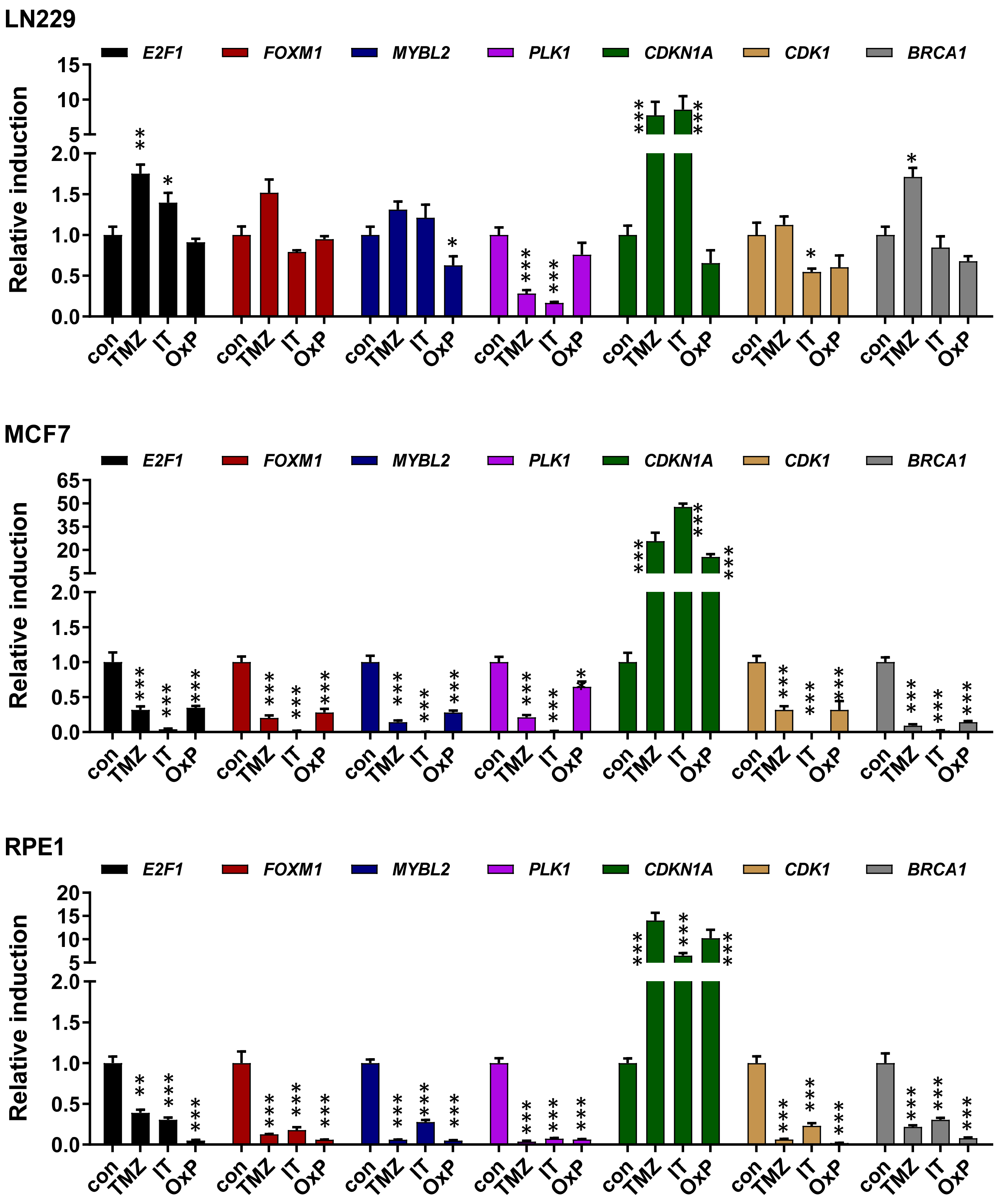
LN229
MCF7
RPE1
E2F1
FOXM1
MYBL2
PLK1
CDKN1A
CDK1
BRCA1
Relative induction
con
TMZ
IT
OxP

Figure S14

**A**

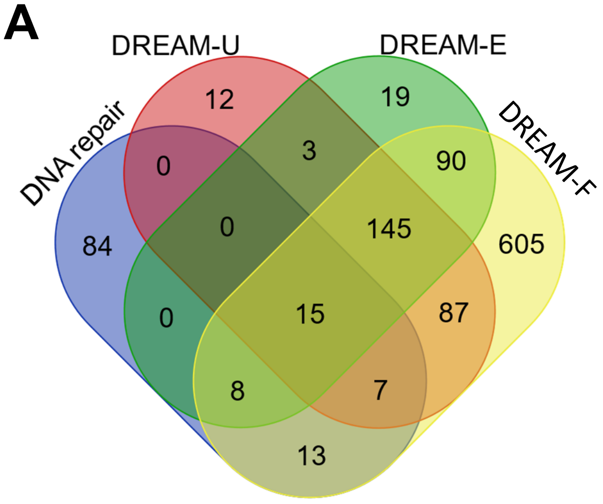


| DNA repair DREAM-F | DNA repair DREAM-E/F/U |
|---|---|
| DCLRE1C H2AX MSH3 PCNA PRKDC RAD1 RAD23B RAD9A TDP1 TOP1 TOP3A WRN XRCC4 | BLM BRCA1 CDC25A EXO1 FANCA FANCB FANCD2 FANCG FANCI FEN1 POLD1 POLE POLQ RAD51 RECQL4 |

| DNA repair DREAM-F/U | DNA repair DREAM-F/E |
|---|---|
| EME1 GEN1 RAD54B TOPBP1 XRCC1 XRCC2 XRCC3 | CDC25B BRCA2 FANCC FANCE FANCL FANCM MSH2 MSH6 |

**B**

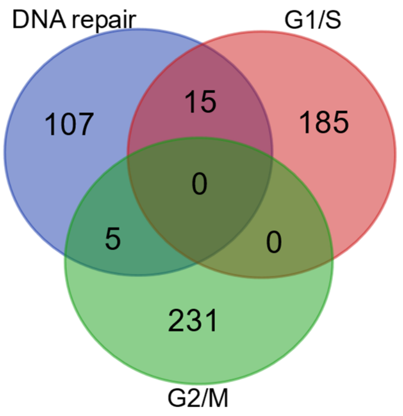


| DNA repair genes (G1/S) | DNA repair genes (G2/M) |
|---|---|
| BLM BRCA1 CDC25A EXO1 FANCE FANCG FEN1 MSH2 MSH6 PCNA POLE RAD51 RECQL4 TREX1 TOPBP1 | CDC25B H2AX POLQ DCLRE1C TDP1 |

**Figure S15**

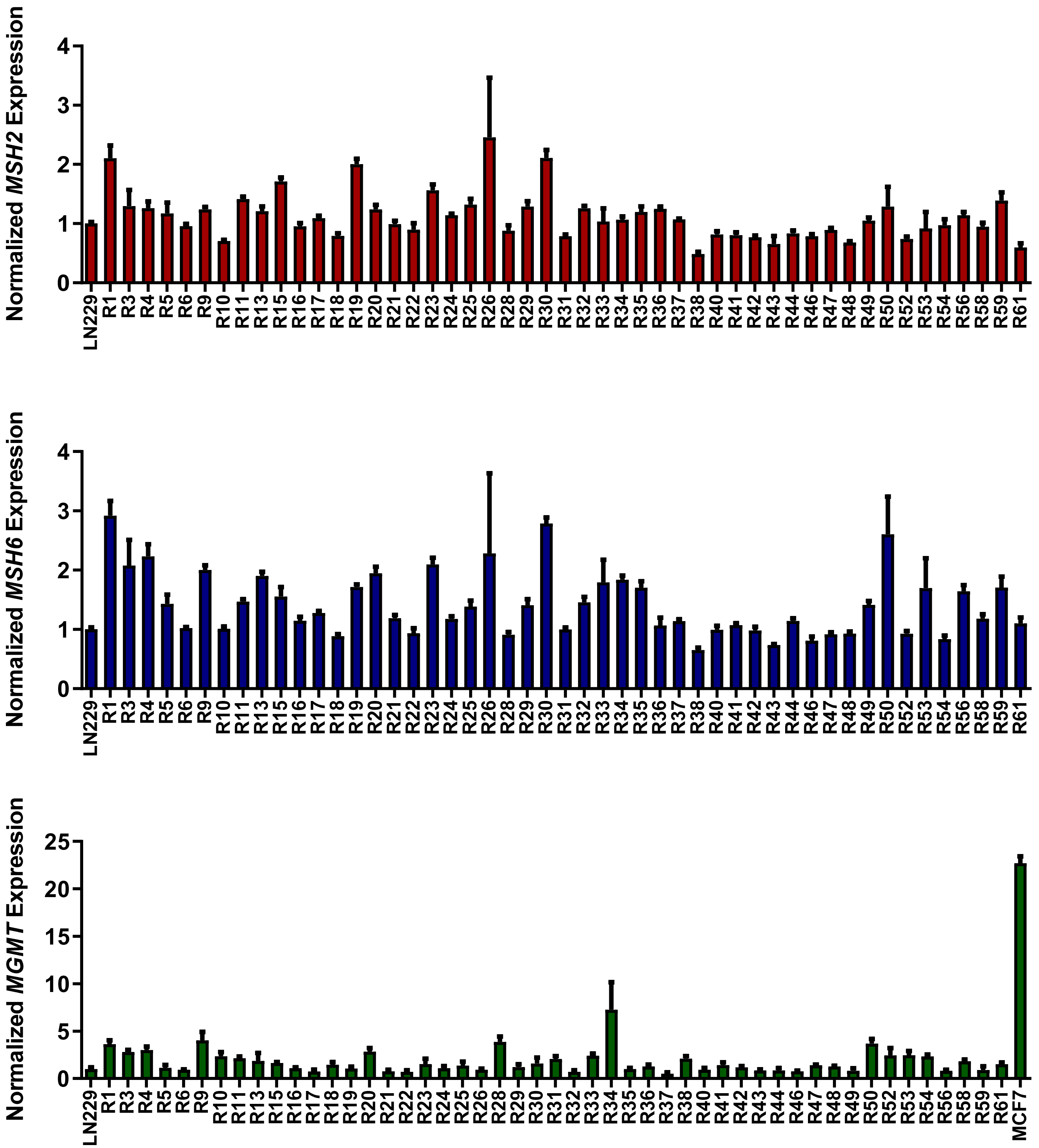
Normalized MSH2 Expression
0
1
2
3
4
LN229 R1 R3 R4 R5 R6 R9 R10 R11 R13 R15 R16 R17 R18 R19 R20 R21 R22 R23 R24 R25 R26 R28 R29 R30 R31 R32 R33 R34 R35 R36 R37 R38 R40 R41 R42 R43 R44 R46 R47 R48 R49 R50 R52 R53 R54 R56 R58 R59 R61
Normalized MSH6 Expression
0
1
2
3
4
LN229 R1 R3 R4 R5 R6 R9 R10 R11 R13 R15 R16 R17 R18 R19 R20 R21 R22 R23 R24 R25 R26 R28 R29 R30 R31 R32 R33 R34 R35 R36 R37 R38 R40 R41 R42 R43 R44 R46 R47 R48 R49 R50 R52 R53 R54 R56 R58 R59 R61
Normalized MGMT Expression
0
5
10
15
20
25
LN229 R1 R3 R4 R5 R6 R9 R10 R11 R13 R15 R16 R17 R18 R19 R20 R21 R22 R23 R24 R25 R26 R28 R29 R30 R31 R32 R33 R34 R35 R36 R37 R38 R40 R41 R42 R43 R44 R46 R47 R48 R49 R50 R52 R53 R54 R56 R58 R59 R61 MCF7

**Figure S16**
