# Supplementary material for: Therapy-induced senescence of glioblastoma cells is determined by the p21CIP1-CDK1/2 axis and does not require activation of DREAM
Source: Cell Death Dis. 2025 May 3;16(1):357. doi: 10.1038/s41419-025-07651-8 (PMC12049523; doi:10.1038/s41419-025-07651-8)
Supplement: Supplementary file 2 — Supplementary figure legends [file 41419_2025_7651_MOESM2_ESM.docx]

**Legends to supplemental figures**

***Fig. S1: A-C)*** LN229 cells were transfected with p21^CIP1^ specific siRNA or nonspecific siRNA and 24 h later exposed to 50 µM TMZ. **A)** Expression of p21^CIP1^ was measured by immunodetection 48 h after TMZ. Quantification of the immunoblot indicates x-fold induction compared to untreated cells. **B)** Proliferation was measured by cell counting 48, 96 and 144 h after TMZ exposure. **C)** Cell death was measured by flow cytometry using PI staining two weeks after TMZ exposure. Differences between the ns-si/TMZ and p21si/TMZ treatment were statistically analyzed using Student's *t* test (non-labelled = not significant; **p<0.01; ***p<0.001). **D)** LN229 cells were exposed to 50 µM TMZ for three weeks and stained with crystal violet. A representative photo shows giant senescent LN229 cells and outgrowing normally sized LN229 cells.

***Fig. S2:*** LN229 cells were exposed to 50 µM TMZ for 144 h. RNA was isolated and subjected to RNAseq. Enhanced and suppressed pathways were identified using the REACTOME and KEGG database via the WEB-based GEne SeT AnaLysis Toolkit (<http://www.webgestalt.org/>). For TMZ up and down-regulated genes with BH adjusted p-values < 0.001 and log2 fold change >2 are included.

***Fig. S3:*** LN229 cells were exposed to 50 µM TMZ for 144 h. RNA was isolated and subjected to RNAseq. The BH adjusted p-values and log2 fold changes mentioned in Fig. S2 were used. **A)** List of p53 targets up-regulated by TMZ. **B)** List of DREAM targets down-regulated by TMZ.

***Fig. S4:*** LN229 cells were exposed to 50 µM TMZ for 48 and 144 h. RNA was isolated and subjected to RNA-Seq. The BH adjusted p-values and log2 fold changes mentioned in Fig. S2 were used. **A)** List of G1/S and G2/M-specific genes down-regulated by TMZ. **B**) Overlap between G1/S and G2/M specific genes with different DREAM-target gene sets. **C**) Overlap between genes downregulated 144 h after TMZ exposure with G1/S, G2/M and DREAM-F target genes and their identity.

***Fig. S5:*** LN229 cells were exposed to 50 µM TMZ for 144 h. RNA was isolated and subjected to RNA-Seq. The BH adjusted p-values and log2 fold changes mentioned in Fig. S2 were used. **A**) Frequency and identity of G1/S- and G2/M-phase specific DREAM-U targets, repressed by TMZ is shown. **B**) Frequency and identity of G1/S- and G2/M-phase specific DREAM-E targets, repressed by TMZ is shown.

***Fig. S6:*** LN229 cells were exposed to 50 µM TMZ for 48 and 144 h. RNA was isolated and subjected to RNA-Seq. List of factors of “microtuble cytoskeleton organization” (MCO) and “mitotic nuclear division” (MND) down-regulated by TMZ. The BH adjusted p-values and log2 fold changes mentioned in Fig. S2 were used.

***Fig. S7:* A**) Overlap between known E2F-RB, FOXM1, MYBL2 and DREAM target genes. **B**) LN229 cells were exposed to 50 µM TMZ for 144 h. RNA was isolated and subjected to RNAseq. Overlap between genes repressed upon TMZ exposure with DREAM, FOXM1, E2F-RB and B-Myb target genes are shown. The BH adjusted p-values and log2 fold changes mentioned in Fig. S2 were used.

***Fig. S8:* A**) Expression of *CDKN2A* (*p14^ARF^)*, *CDKN2A* (*p16^INK4^)* and *CDKN1A* (*p21^CIP1^)* was analyzed by RT-PCR in LN229, U87, A172 and LN308 cells. **B**) LN229, U87MG and A172 cells were exposed to 50 µM TMZ for 72 or 144 h. The expression of *p21^CIP1^* was analyzed by RT-PCR. **A/B)** *ACTB* and *GAPDH* were used as internal standard and expression of untreated LN229 cells was set to 1. Differences between the cell lines (A) and between control and TMZ treatment (B) were statistically analyzed using Student's *t* test (non-labelled = not significant; **p<0.01; ***p<0.001). **C**) LN229, U87MG and A172 cells were exposed to 50 µM TMZ for 48 h. The expression and phosphorylation of p53 was analyzed by immunodetection. HSP90 was used as internal standard. **D**) LN229, U87MG, A172 and LN308 cells were exposed to 50 µM TMZ for 144 h. The expression of p14^ARF^, p16^INK4A^ and p21^CIP1^ was analyzed by immunodetection. GAPDH was used as internal standard. **E**) Potential deletions and CpG-methylation of *CDKN2A* (*p14^ARF^)*, *CDKN2A* (*p16^INK4^)* were analyzed using methylation specific PCR (MSP) in LN229, A172, U87MG and LN308 cells. U=unmethylated, M=methylated, A=ACTB. Combined absence of a signal for the methylated and unmethylated sequence and presence of a signal for the Actin specific sequence indicates a gene deletion.

***Fig. S9:*** LN229, U87MG and A172 cells were exposed to 50 µM TMZ for 144 h. Expression of various SASP factors was measured by qPCR. *ACTB* and *GAPDH* were used as internal standard and expression of the untreated control was set to 1. Differences between the control and TMZ treatment were statistically analyzed using Student's *t* test (non-labelled = not significant; **p<0.01; ***p<0.001).

***Fig. S10:*** A172 and U87MG cells were exposed for 144 h to 50 µM TMZ. The expression of *CCNA1, CCNA2, CCNB1, CCNB2, CCND1, CCND2, CCNE1 and CCNE2* was analyzed by qPCR. *ACTB* and *GAPDH* were used as internal standard and expression of the untreated control was set to 1. Differences between the control and TMZ treatment were statistically analyzed using Student's *t* test (non-labelled = not significant; *p<0.1; **p<0.01; ***p<0.001).

***Fig. S11:*** U87MG cells (2.5 x10^6^) were injected in the left and the right flank of four female immunodeficient mice (BALB/cAnNRj-*Foxn1^nu/nu^*, Janvier Labs) to induce subcutaneous xenografts. At a suitable size (22 mm^3^), two randomly selected female animals (M1 and M2) were injected with the solvent (DMSO) only and two animals (M3 and M4) with TMZ (200 mg/kg body weight in DMSO/NaCl i.p.). After 96 h, the mice were sacrificed, the tumours were isolated and immediately frozen in liquid nitrogen. For expression analysis, the left and right tumour were combined and the tissue was disintegrated using a tissue lyser (Retsch). Whole cell protein extract and RNA was isolated. The investigator received the tumor samples encoded by numbers and was not aware of its origin. **A)** Expression and phosphorylation of p130 and Rb1, as well as expression of p21^CIP1^ was mesured by immunodetection. **B/C)** Transcriptional expression of various DREAM targets and cell cycle regulators was measured by qPCR. *ACTB* and *GAPDH* were used as internal standard and expression of the untreated control was set to 1. Differences between the control and TMZ treatment were statistically analyzed using Student's *t* test (non-labelled = not significant; *p<0.1; **p<0.01; ***p<0.001).

***Fig. S12:*** LN229, A172 and U87MG cells were exposed for 144 h to either 50 µM TMZ, 1 µM Palbociclib or a combination of both. The expression of *MSH2, EXO1, MSH6, E2F1, FOXM1 and MYBL2* was analysed by RT-PCR. *ACTB* and *GAPDH* were used as internal standard and expression of the untreated control was set to 1. Differences between the control and treatment were statistically analyzed using Student's *t* test (non-labelled = not significant; *p<0.1; **p<0.01; ***p<0.001).

***Fig S13:*** **A)** LN229, MCF7 and RPE1 cells were exposed to different concentrations of TMZ, Irinotecan or Oxaliplatin for 144 h. Cell death and cell cycle distribution were measured by flow cytometry using PI staining. **B)** LN229 and MCF7 cells were exposed to TMZ (LN229/RPE1: 50 µM, MCF7: 500 µM), Irinotecan (5 µM) or Oxaliplatin (10 µM) for 144 h. Frequency of senescent cells was detected microscopically by SA-β-Gal staining. Differences between treatment and control were statistically analyzed using Student's *t* test (***p<0.001). **A/B)**. Experiments were performed in triplicates. We should note that in MCF7 cells the MGMT inhibitor O^6^-BG (10 µM) and high TMZ concentrations had to be used in order to efficiently circumvent the high MGMT expression in these cells.

***Fig S14:*** LN229, MCF7 and RPE1 cells were exposed to TMZ (LN229/RPE1: 50 µM, MCF7: 500 µM), irinotecan (5 µM) or oxaliplatin (10 µM) for 144 h. Expression of various DREAM factors was measured by qPCR. *ACTB* and *GAPDH* were used as internal standard and expression of the untreated control was set to 1. Differences between the control and TMZ treatment were statistically analyzed using Student's *t* test (non-labelled = not significant; *p<0.1; **p<0.01; ***p<0.001).

***Fig. S15:* A)** Overlap between DNA repair genes and DREAM targets is shown by Venn diagrams; the identity of the DNA repair genes identified as DREAM targets is listed. **B)** Overlap between DNA repair genes and G1/S and G2/M specific genes is shown by Venn diagrams; the identity of the cell-cycle regulated genes is listed. DREAM targets are depicted in red.

***Fig. S16:*** LN229 cells were exposed to 50 µM TMZ. Surviving and proliferating cell clones were picked and further cultivated to establish senescence-escaping/evading cell clones. Expression of *MSH2, MSH6* and *MGMT* were measured by qPCR in all clones and the parental LN229 cell line. For MGMT as positive control, MCF7 cells were included. *ACTB* and *GAPDH* were used as internal standard and expression of the untreated control was set to 1.
