# Supplementary material for: Therapy-induced senescence of glioblastoma cells is determined by the p21CIP1-CDK1/2 axis and does not require activation of DREAM
Source: Cell Death Dis. 2025 May 3;16(1):357. doi: 10.1038/s41419-025-07651-8 (PMC12049523; doi:10.1038/s41419-025-07651-8)

Immunodetection was performed
using the iBright CL1000 (Invitrogen) system,
directly scanning the area of interest.

Dotted lines show the size of the scanned area.

If not stated differentially, detection of the protein of interest and loading controls (HSP90/β-Actin) were performed on the same membrane after stripping the first antibody.

In several cases, the membrane was cut, to perform detection of different proteins at the same time.

Fig. 2A

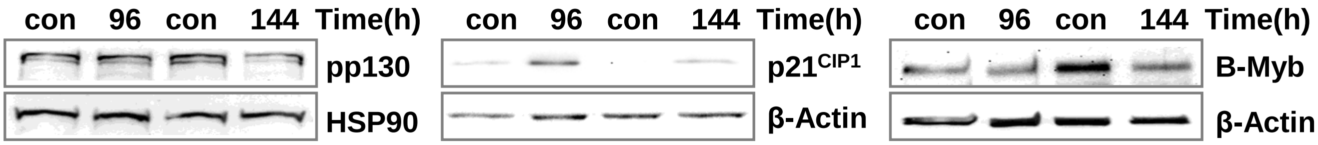

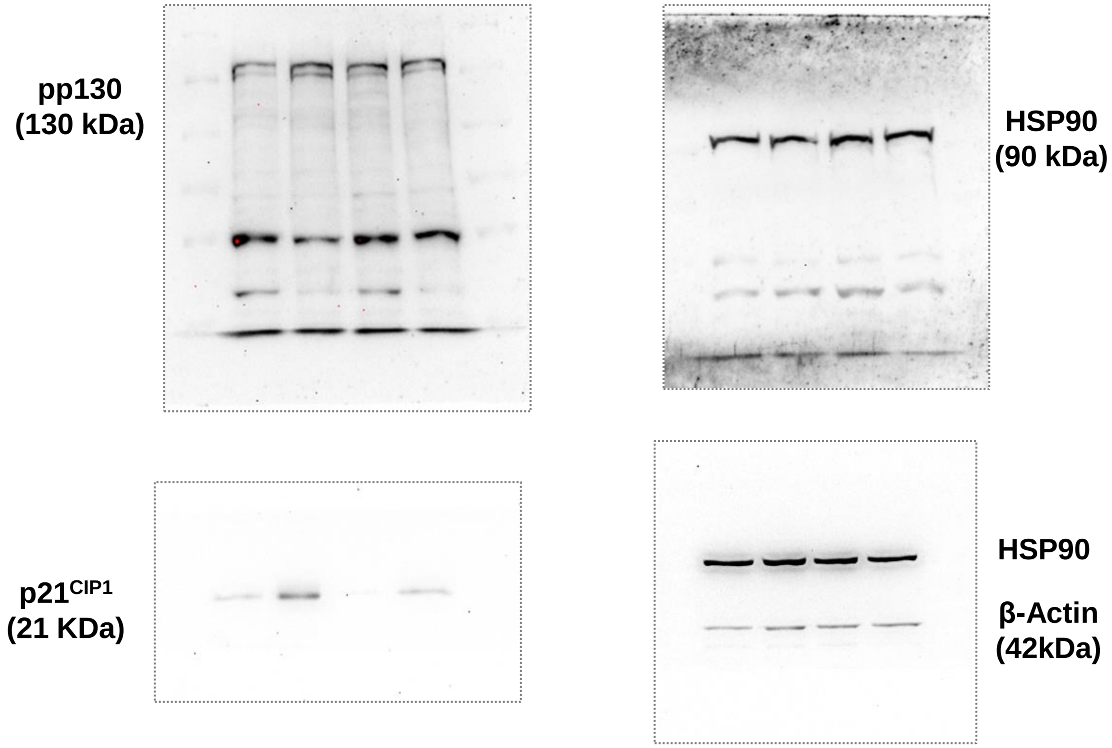

Same loading control as for E2F5
Blot was cut after detection of the loading control at 35 kDa

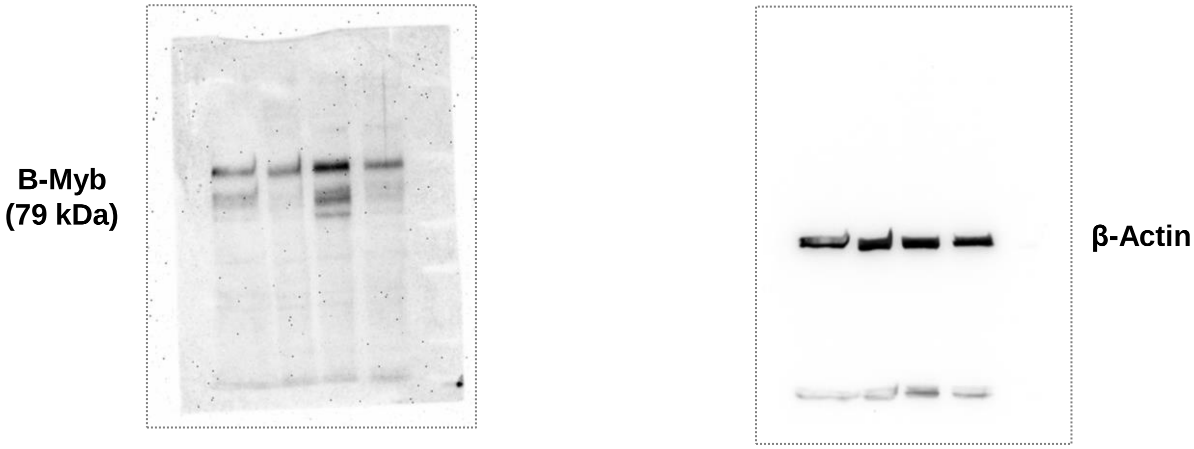

Fig. 2A

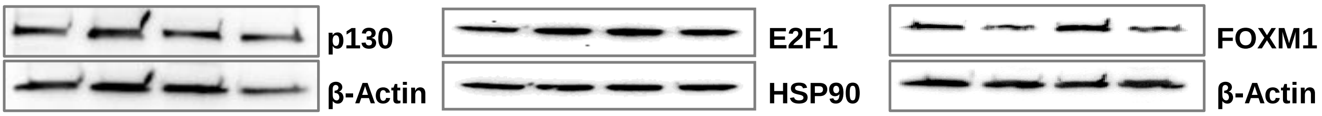

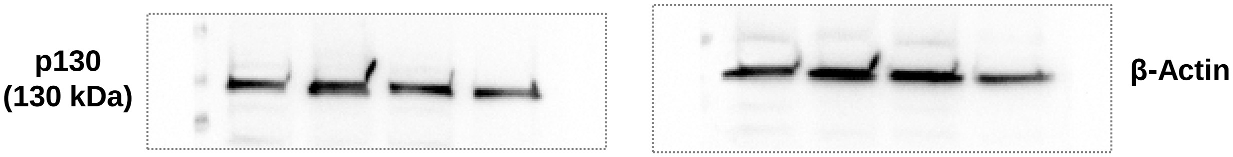

Blot was cut before seperate antibody incubation

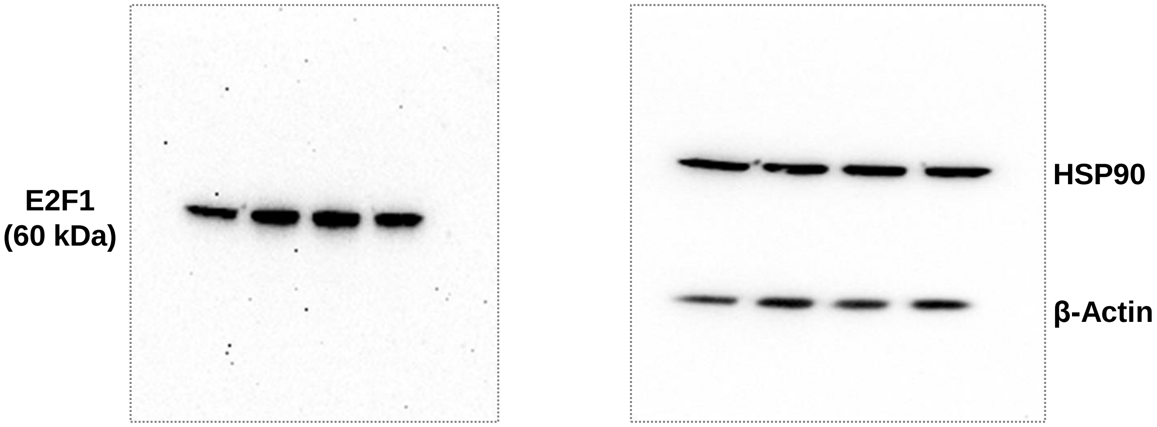

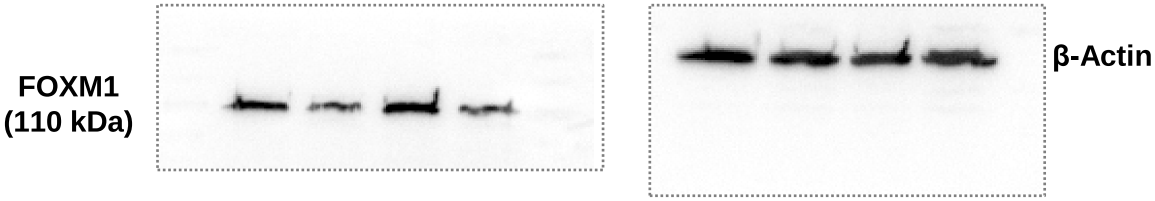

Blot was cut before seperate antibody incubation

Fig. 2A

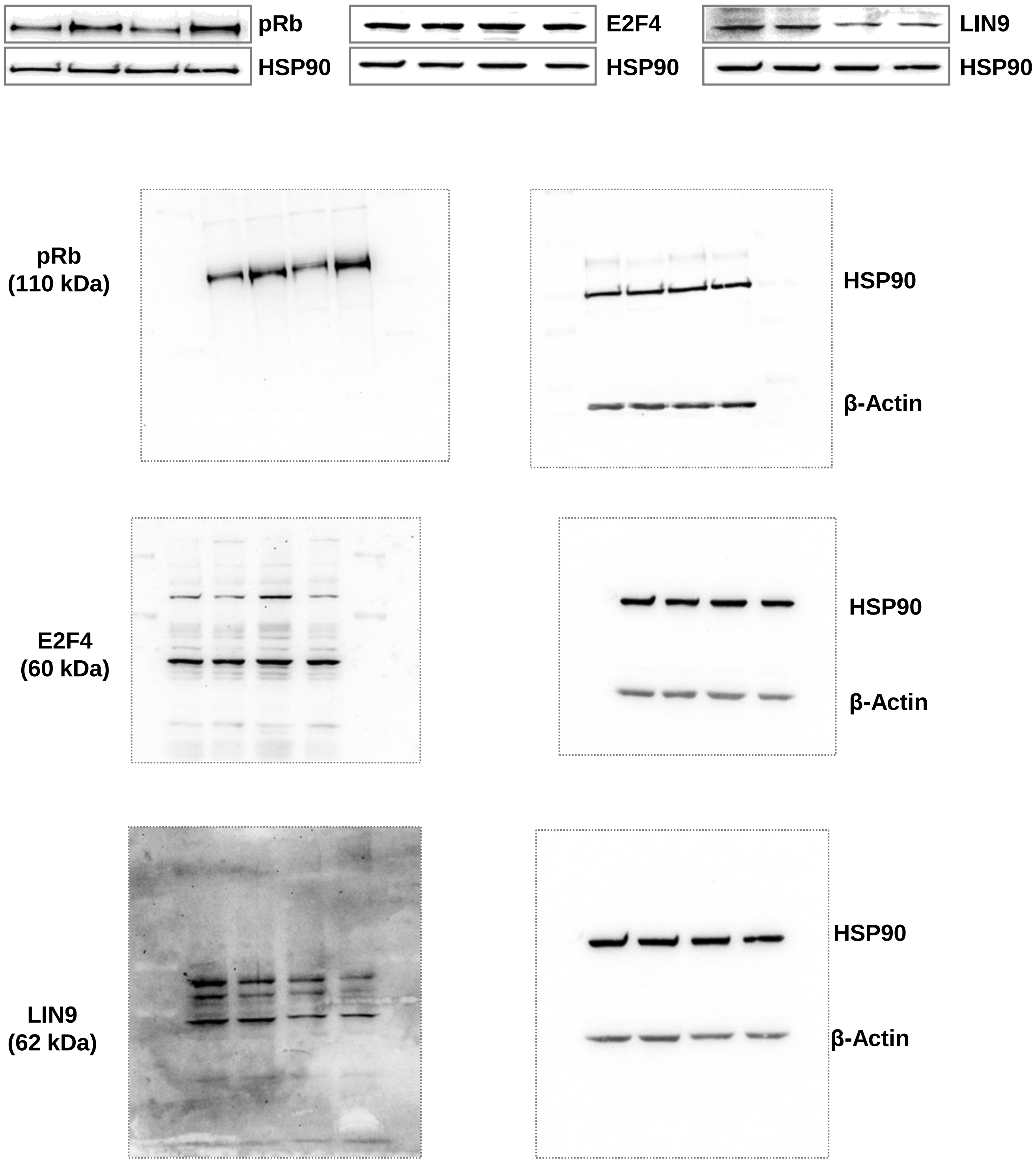
pRb
HSP90
E2F4
HSP90
LIN9
HSP90
pRb
(110 kDa)
HSP90
β-Actin
E2F4
(60 kDa)
HSP90
β-Actin
LIN9
(62 kDa)
HSP90
β-Actin

Fig. 2A

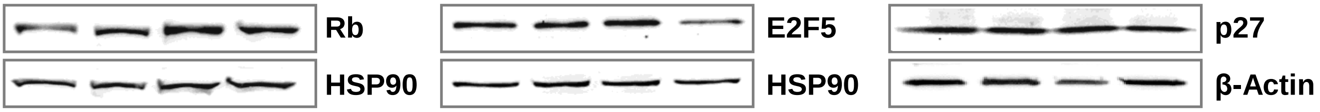

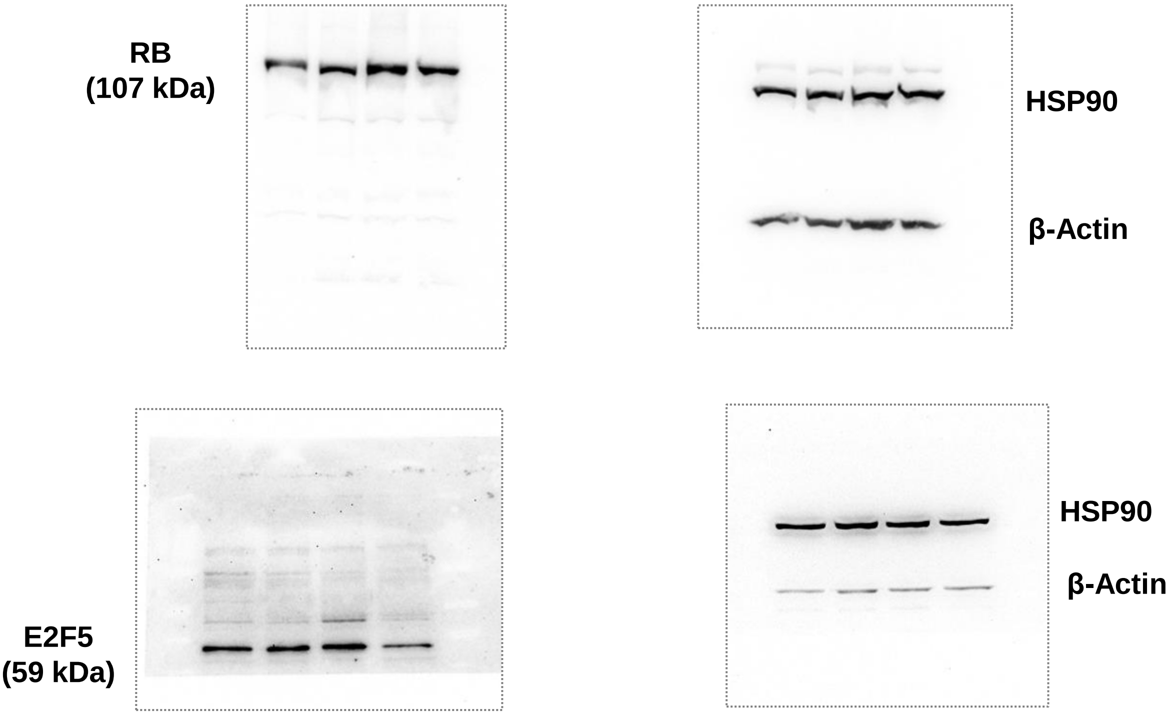

Same loading control as for p21
Blot was cut after detection of the loading control at 30 kDa

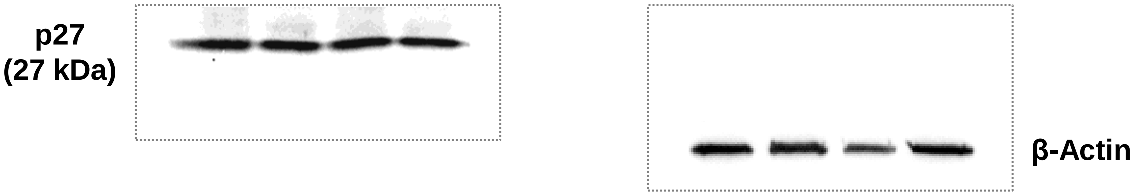

Blot was cut before seperate antibody incubation

Fig. 4A

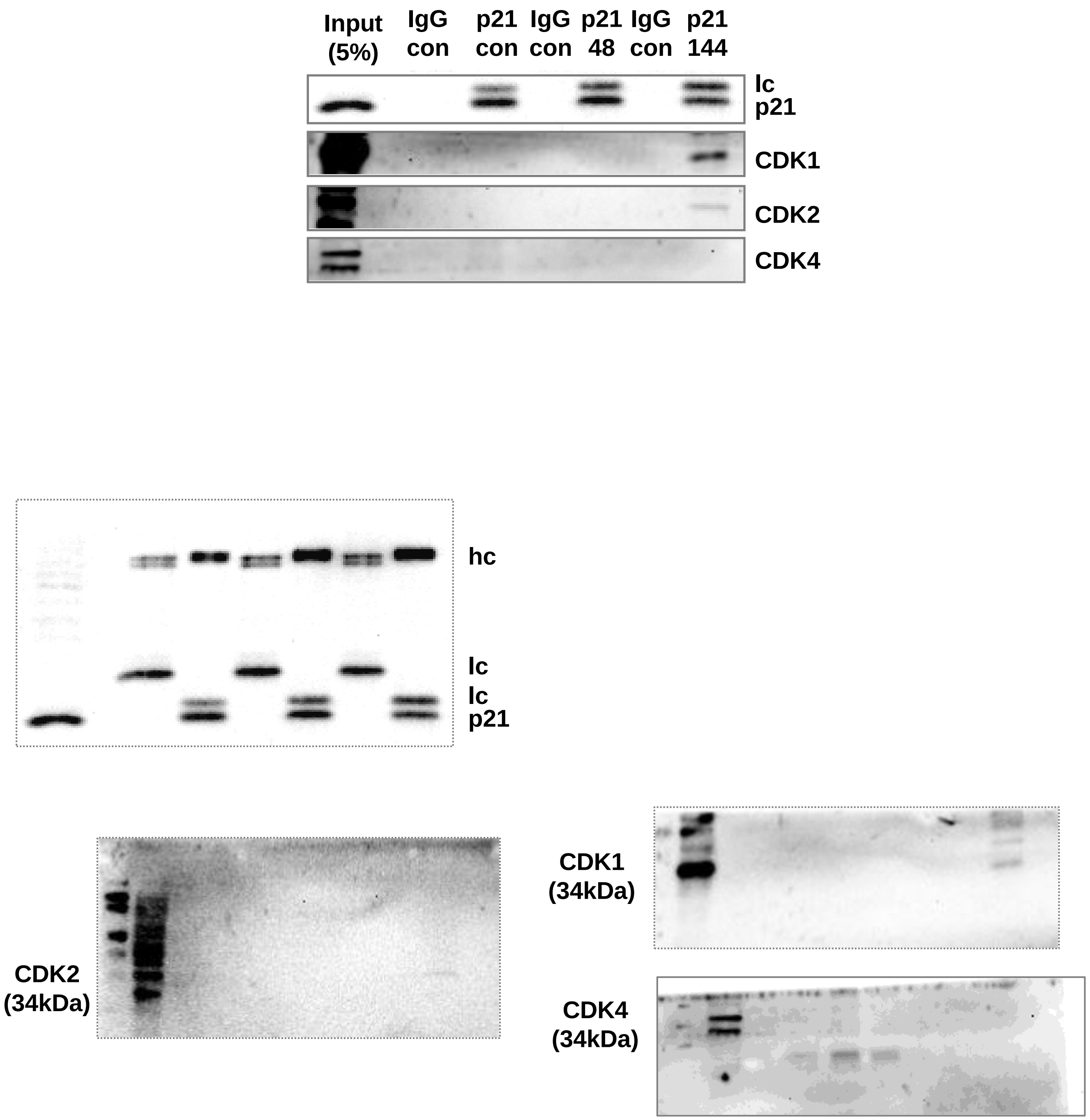
Input (5%)
IgG con
p21 con
IgG con
p21 48
IgG con
p21 144
lc
p21
CDK1
CDK2
CDK4
hc
lc
lc
p21
CDK2 (34kDa)
CDK1 (34kDa)
CDK4 (34kDa)

Fig. 4B

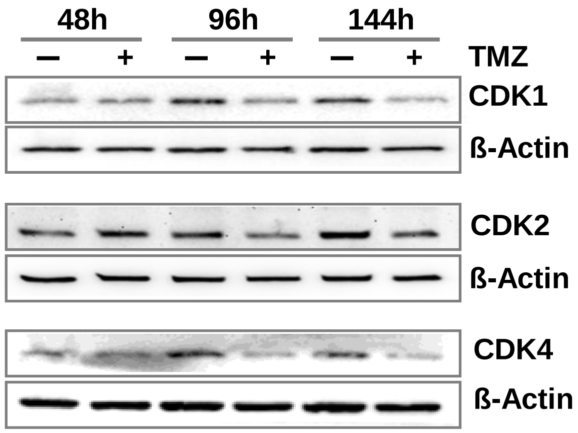

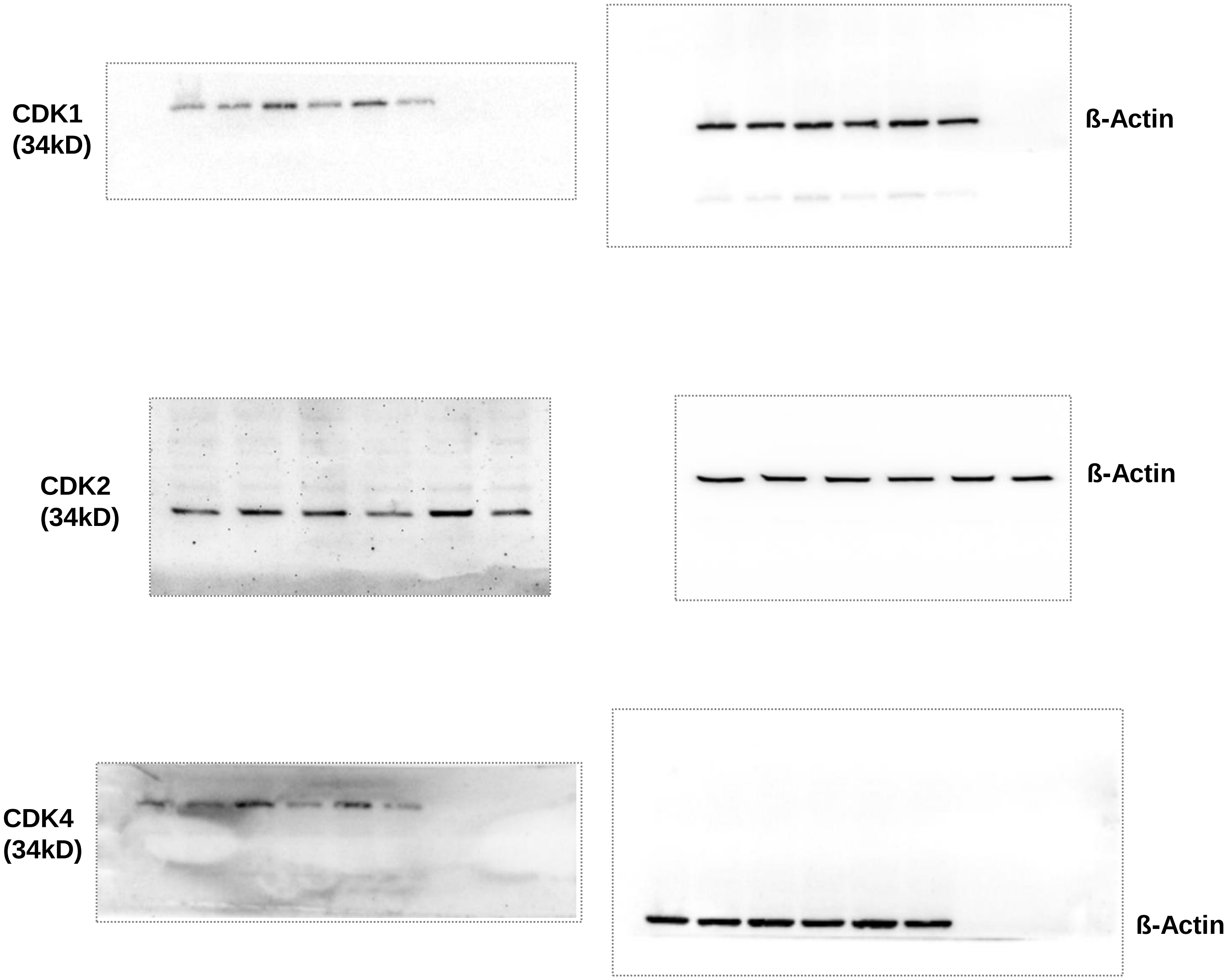

Blot was cut before seperate antibody incubation

Fig. 4C

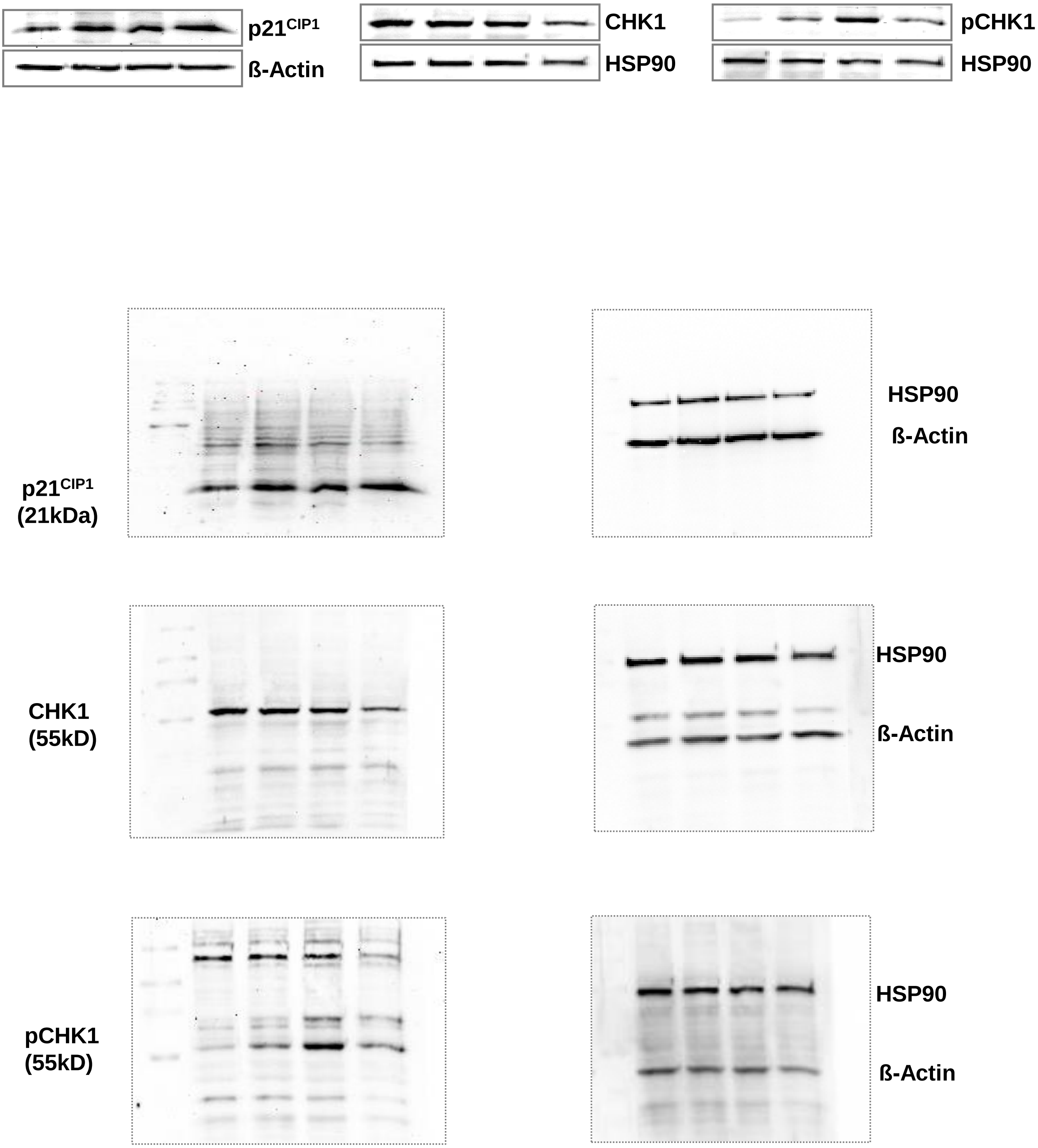

p21CIP1
ß-Actin
CHK1
HSP90
pCHK1
HSP90
HSP90
ß-Actin
p21CIP1
(21kDa)
CHK1
(55kD)
HSP90
ß-Actin
pCHK1
(55kD)
HSP90
ß-Actin

Fig. 4C

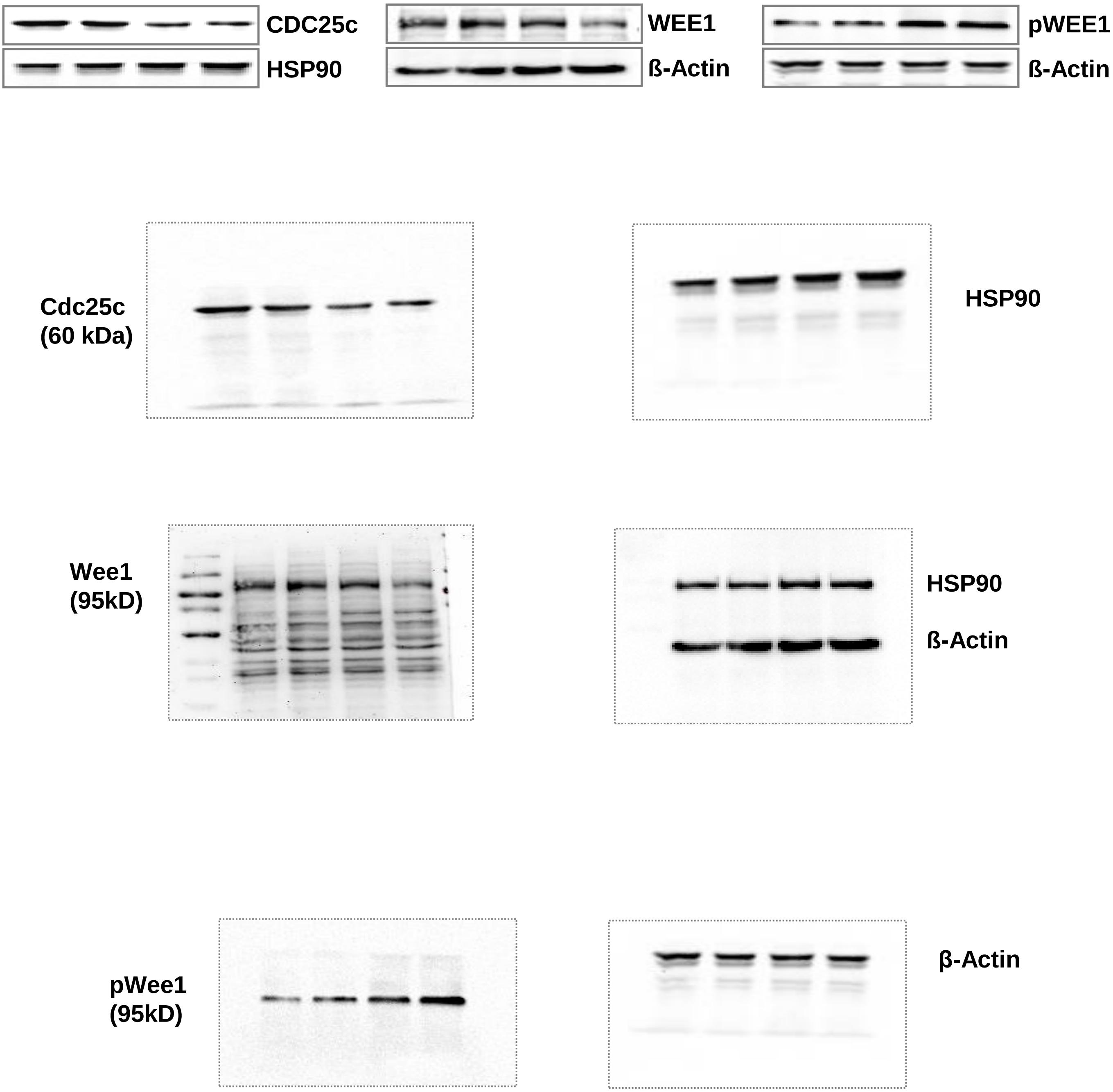

Blot was cut before seperate antibody incubation

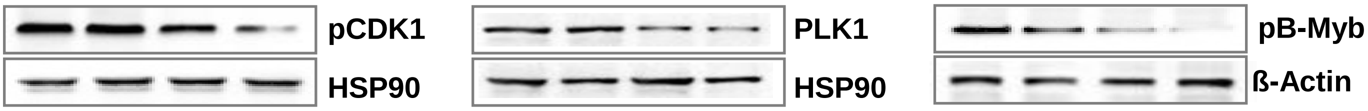

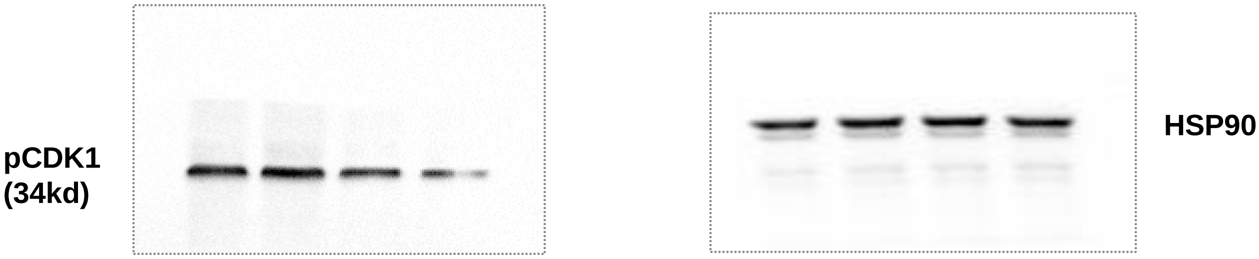

Blot was cut before seperate antibody incubation

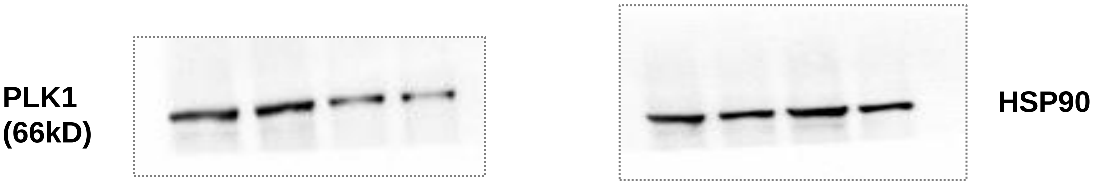

Blot was cut before seperate antibody incubation

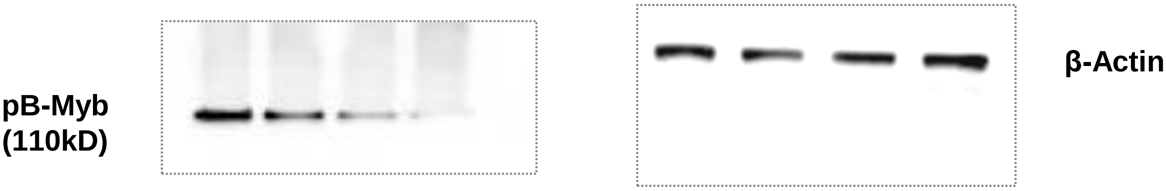

Blot was cut before seperate antibody incubation

Fig. 4D

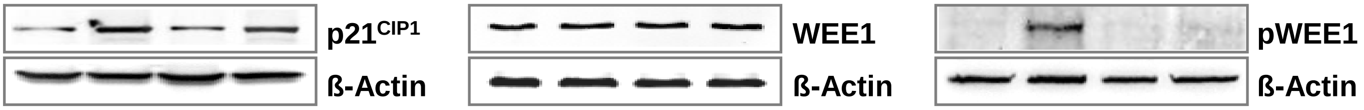

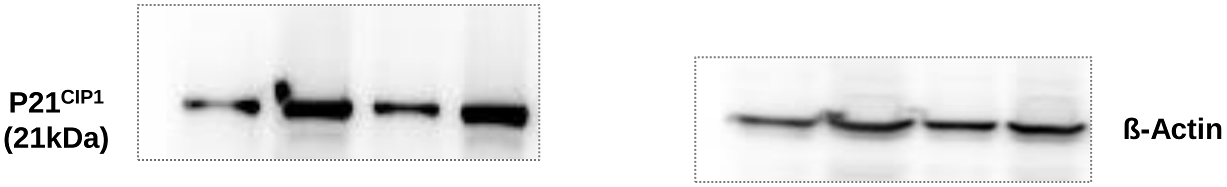

Blot was cut before seperate antibody incubation

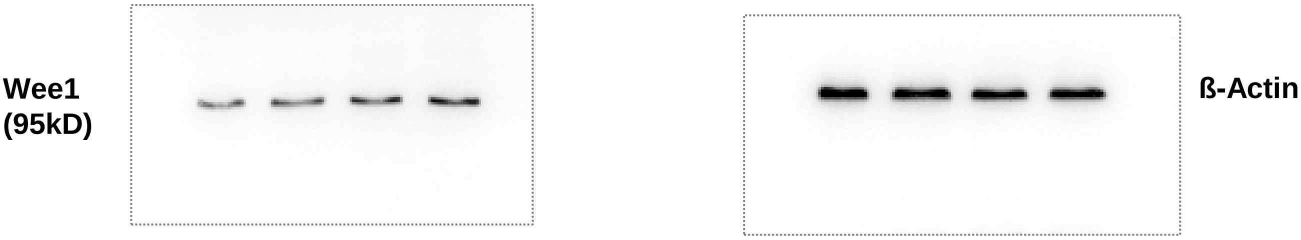

Blot was cut before seperate antibody incubation

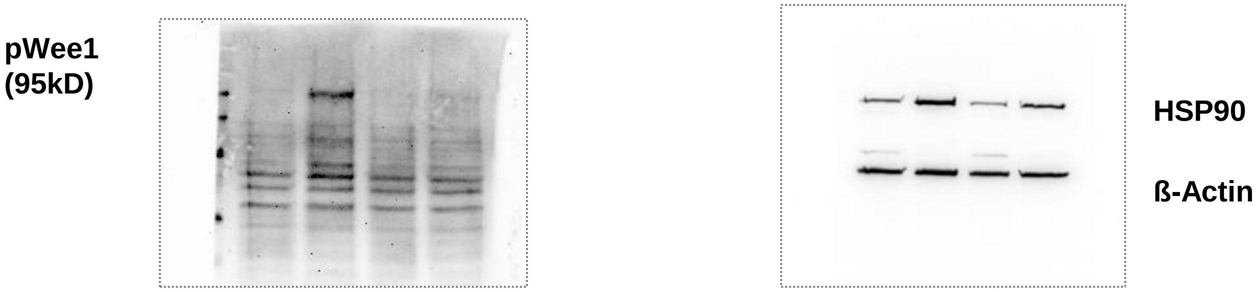

Fig. 4D

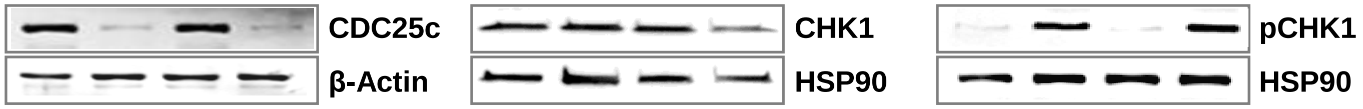

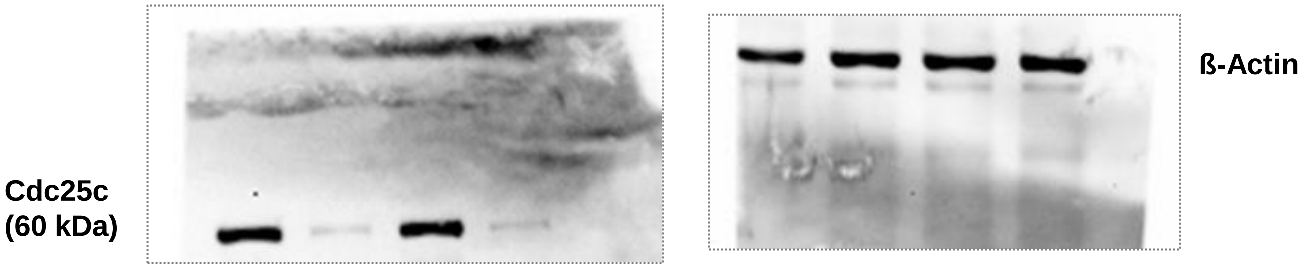

Blot was cut before seperate antibody incubation

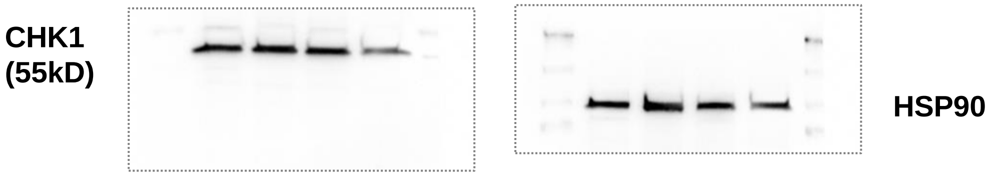

Blot was cut before seperate antibody incubation

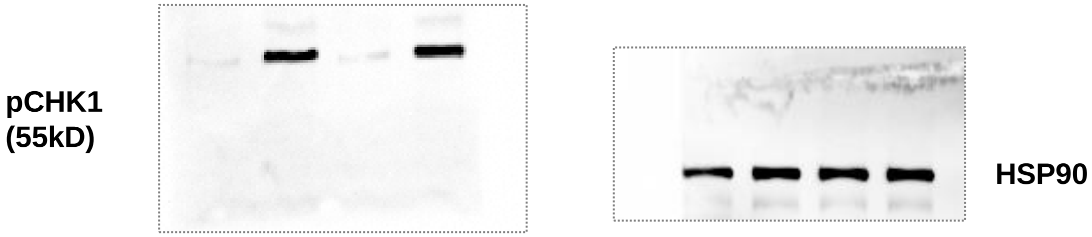

Blot was cut before seperate antibody incubation

Fig. 4D

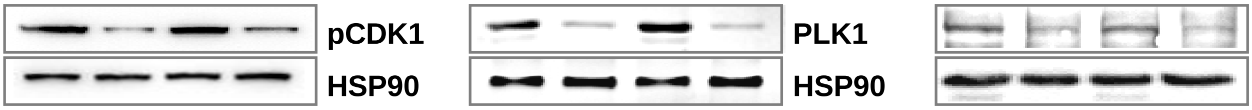

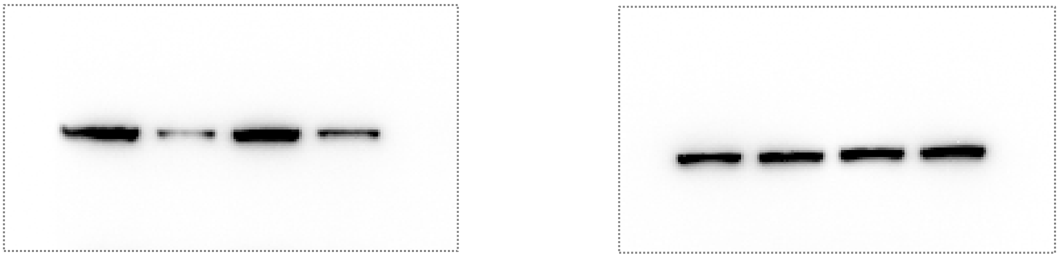

Blot was cut before seperate antibody incubation

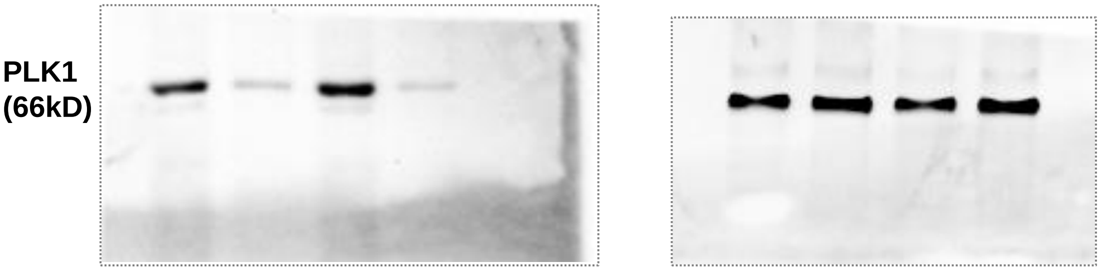

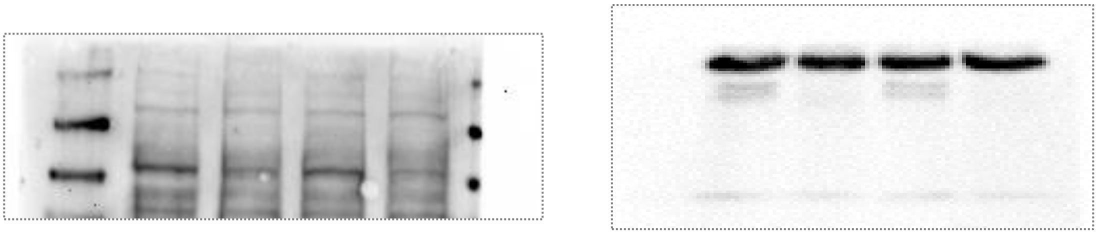

Blot was cut before seperate antibody incubation

Fig. 6A - A172

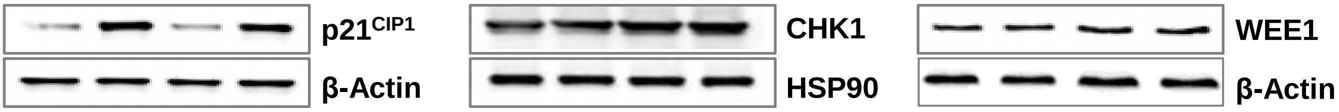

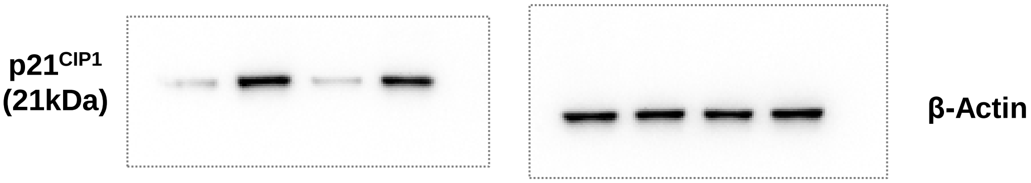

Blot was cut before seperate antibody incubation

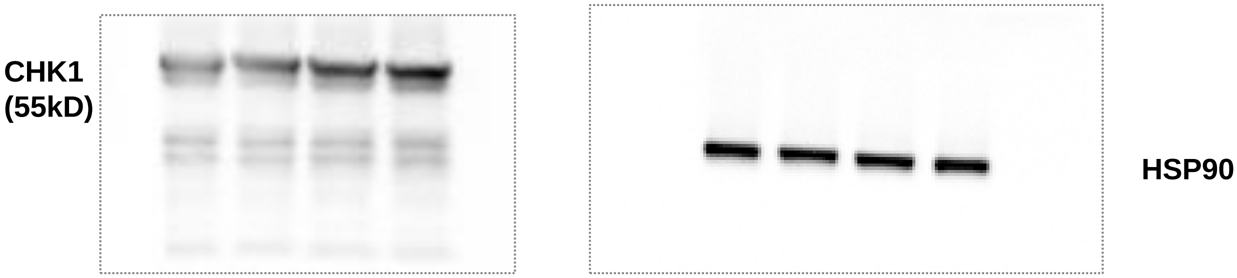

Blot was cut before seperate antibody incubation

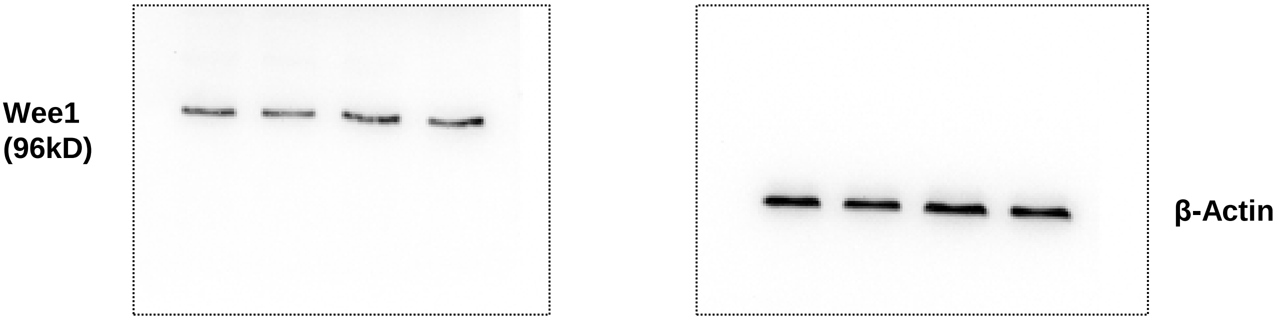

Fig. 6A - A172

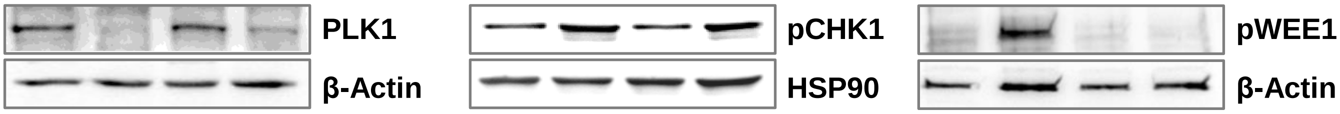

PLK1 β-Actin pCHK1 HSP90 pWEE1 β-Actin

PLK1 (66kD)

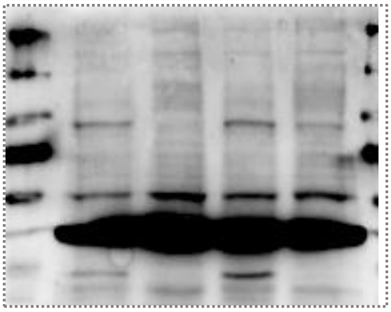

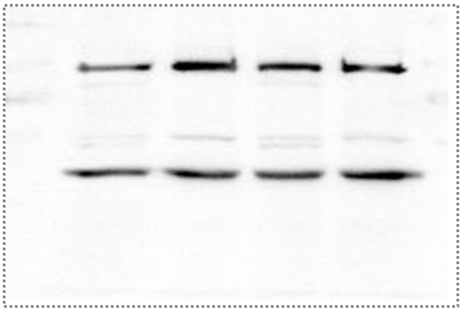

HSP90

ß-Actin

pCHK1 (55kD)

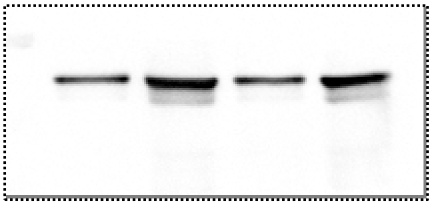

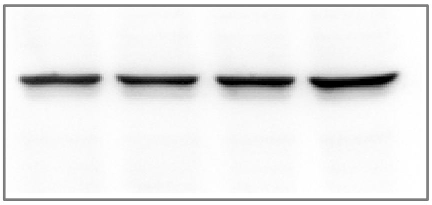

HSP90

Blot was cut before seperate antibody incubation

pWee1 (96kD)

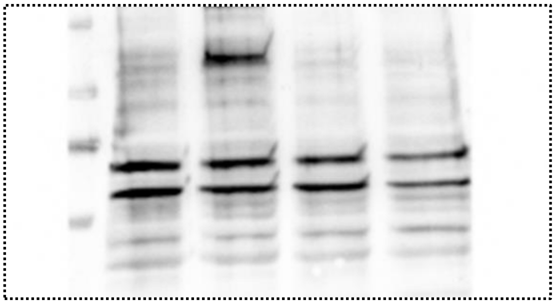

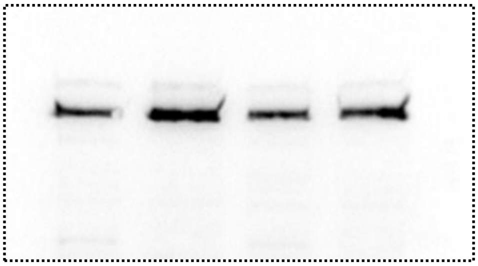

ß-Actin

Fig. 6A - A172

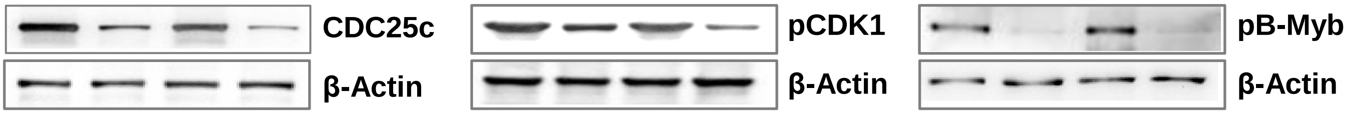

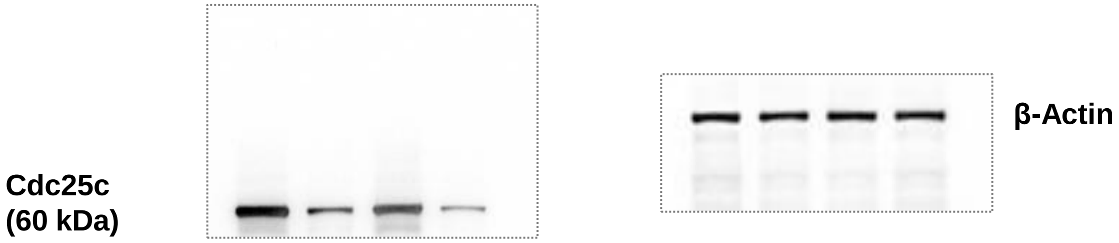

Blot was cut before seperate antibody incubation

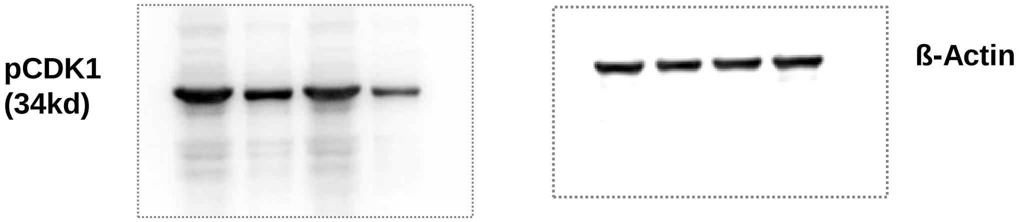

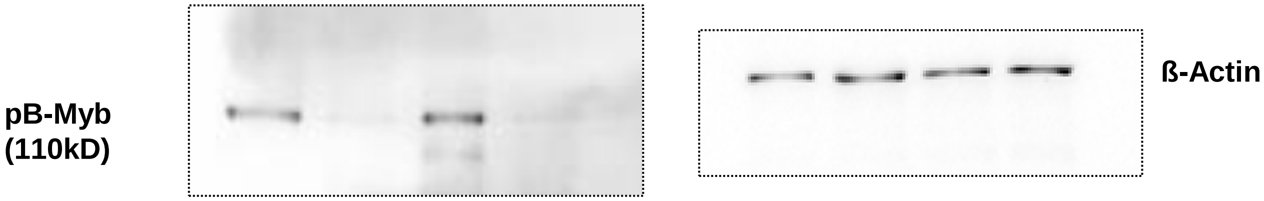

Blot was cut before seperate antibody incubation

Fig. 6A - U87

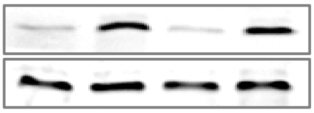

p21CIP1
β-Actin

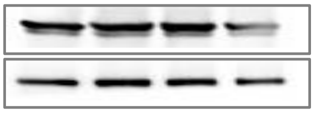

CHK1
HSP90

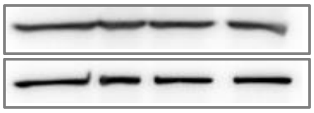

WEE1
β-Actin

p21CIP1
(21kDa)

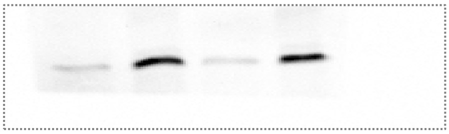

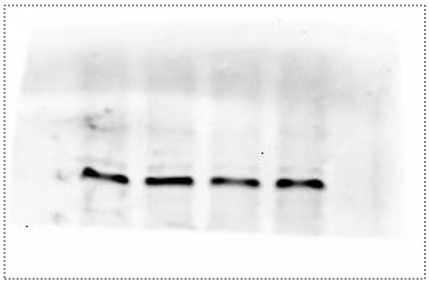

β-Actin

Blot was cut before seperate antibody incubation

CHK1
(55kD)

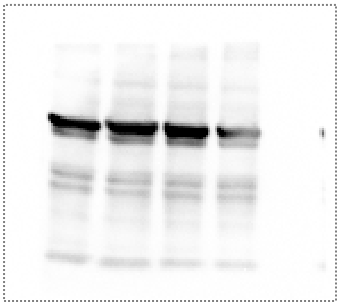

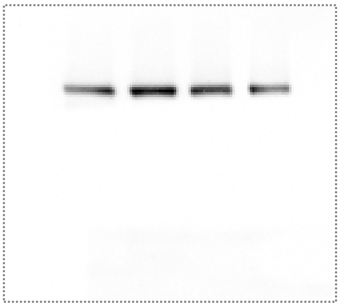

HSP90

Wee1
(95kD)

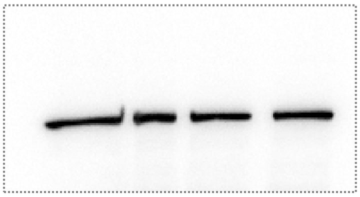

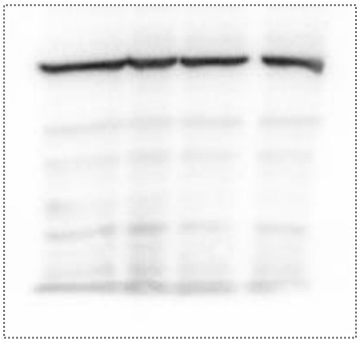

β-Actin

Blot was cut before seperate antibody incubation

Fig. 6A - U87

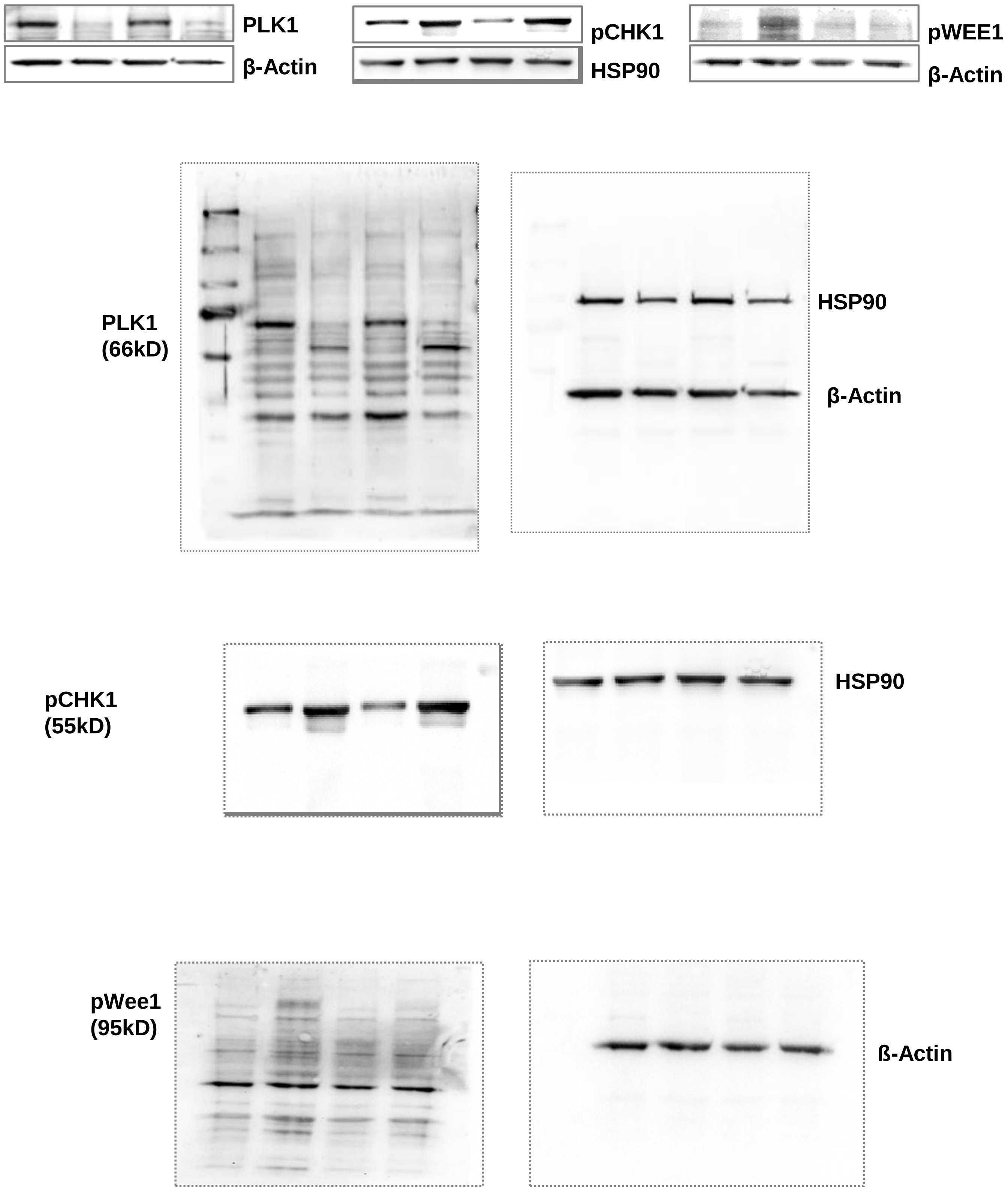
PLK1
β-Actin
pCHK1
HSP90
pWEE1
β-Actin
PLK1
(66kD)
HSP90
β-Actin
pCHK1
(55kD)
HSP90
pWee1
(95kD)
ß-Actin

Fig. 6A - U87

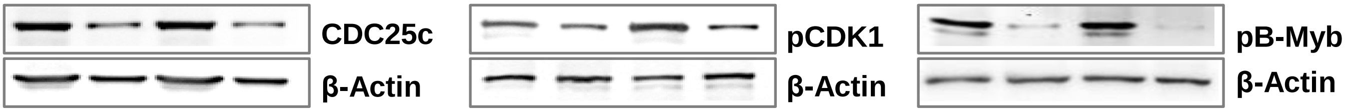

Cdc25c
(60 kDa)

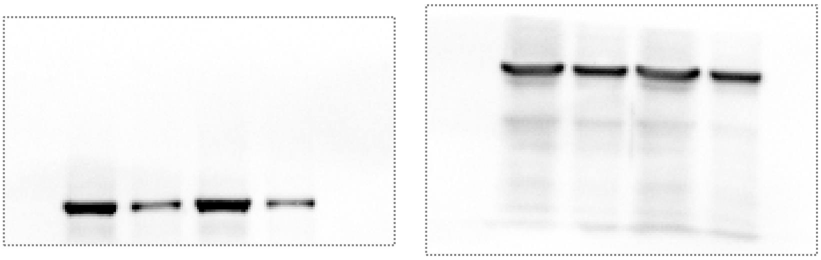

β-Actin

Blot was cut before seperate antibody incubation

pCDK1
(34kd)

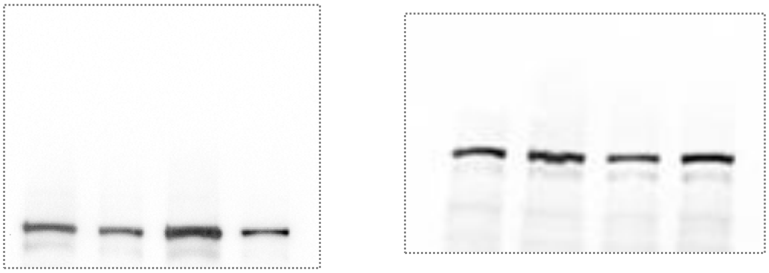

β-Actin

pB-Myb
(110kD)

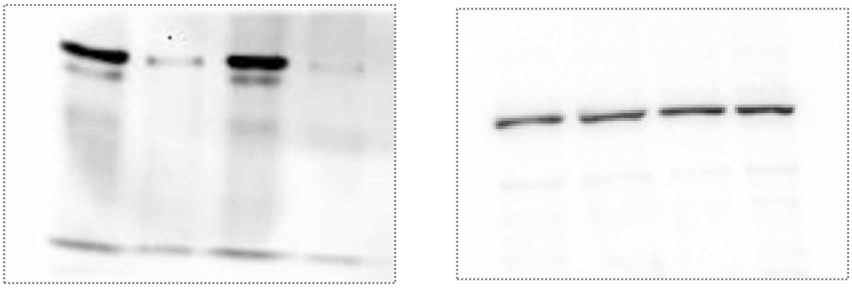

β-Actin

Figure 6B

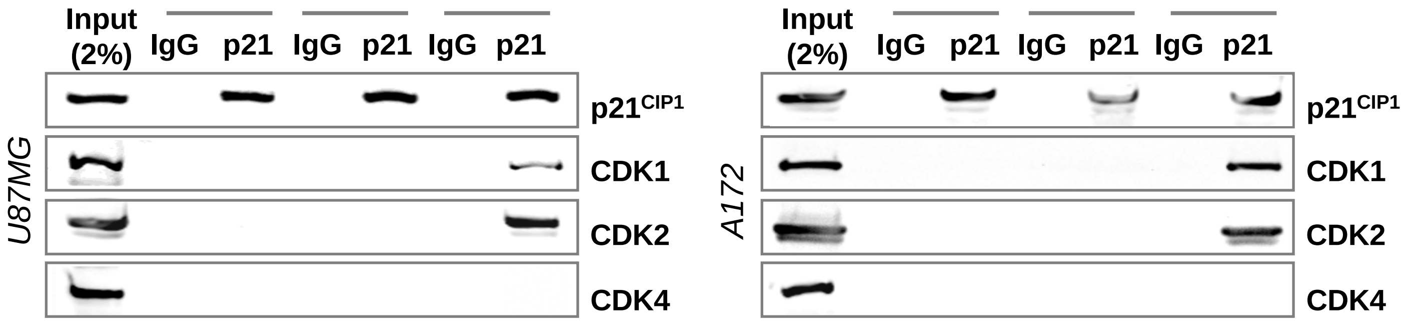

Input (2%)
IgG
p21
IgG
p21
IgG
p21
p21CIP1
CDK1
CDK2
CDK4
U87MG
Input (2%)
IgG
p21
IgG
p21
IgG
p21
p21CIP1
CDK1
CDK2
CDK4
A172

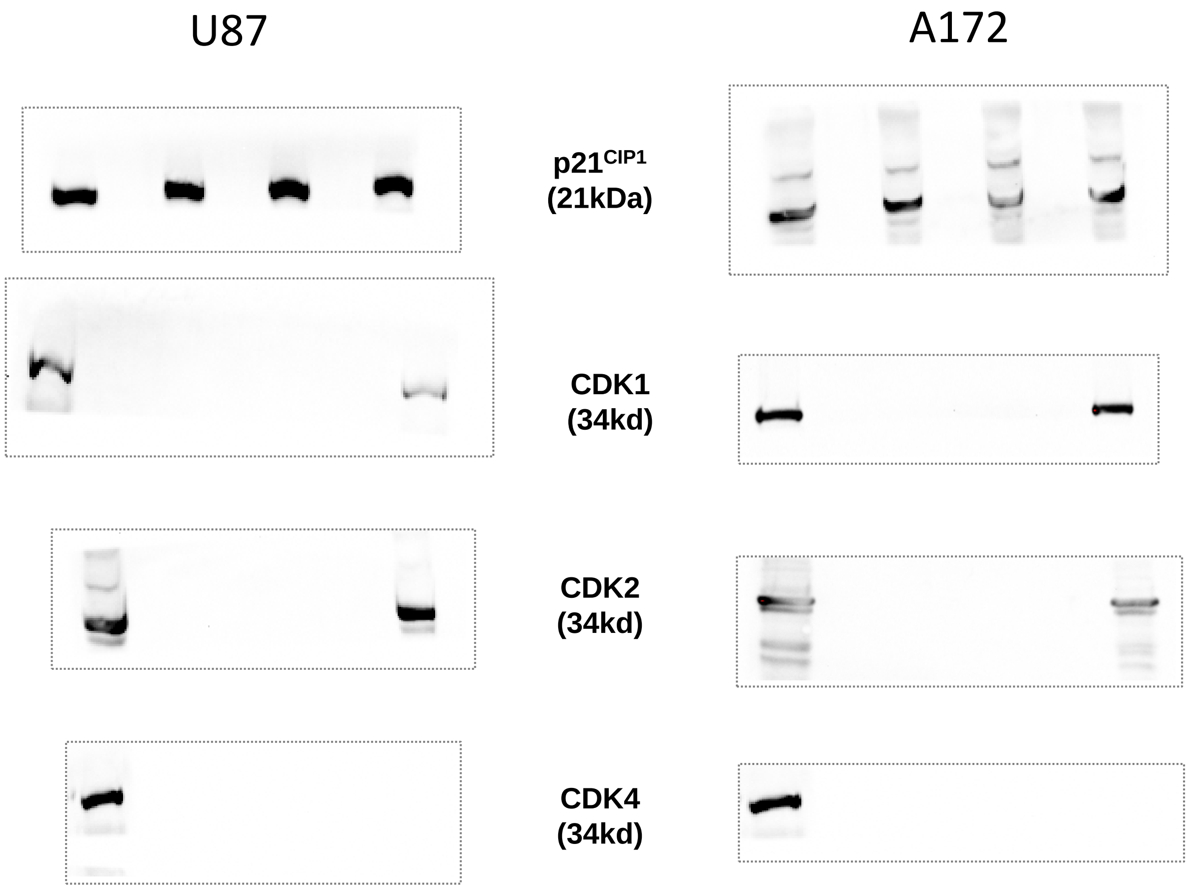

U87
A172
p21CIP1 (21kDa)
CDK1 (34kd)
CDK2 (34kd)
CDK4 (34kd)

Figure 7E

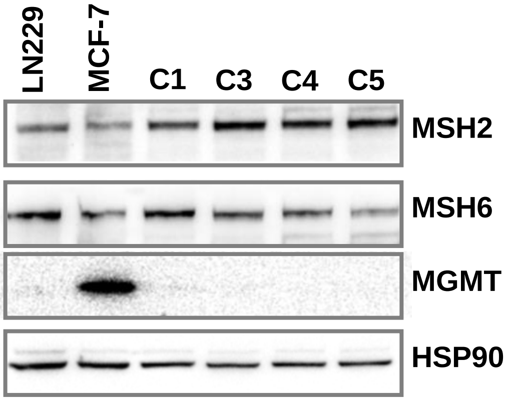

Blot was cut at 50 kD before seperate antibody incubation

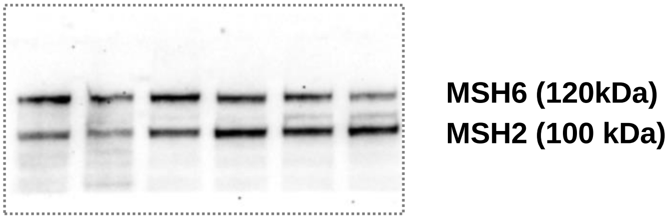

Simoultanous incubation with antibodies against MSH2 and MSH6

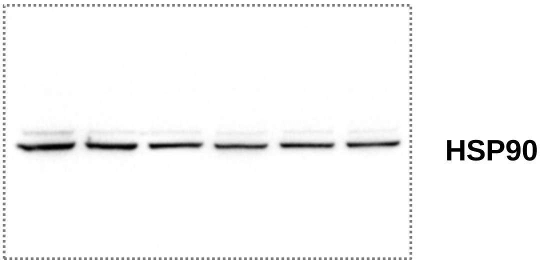

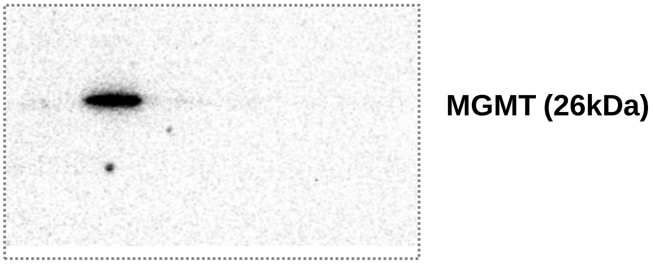

Fig. S8C

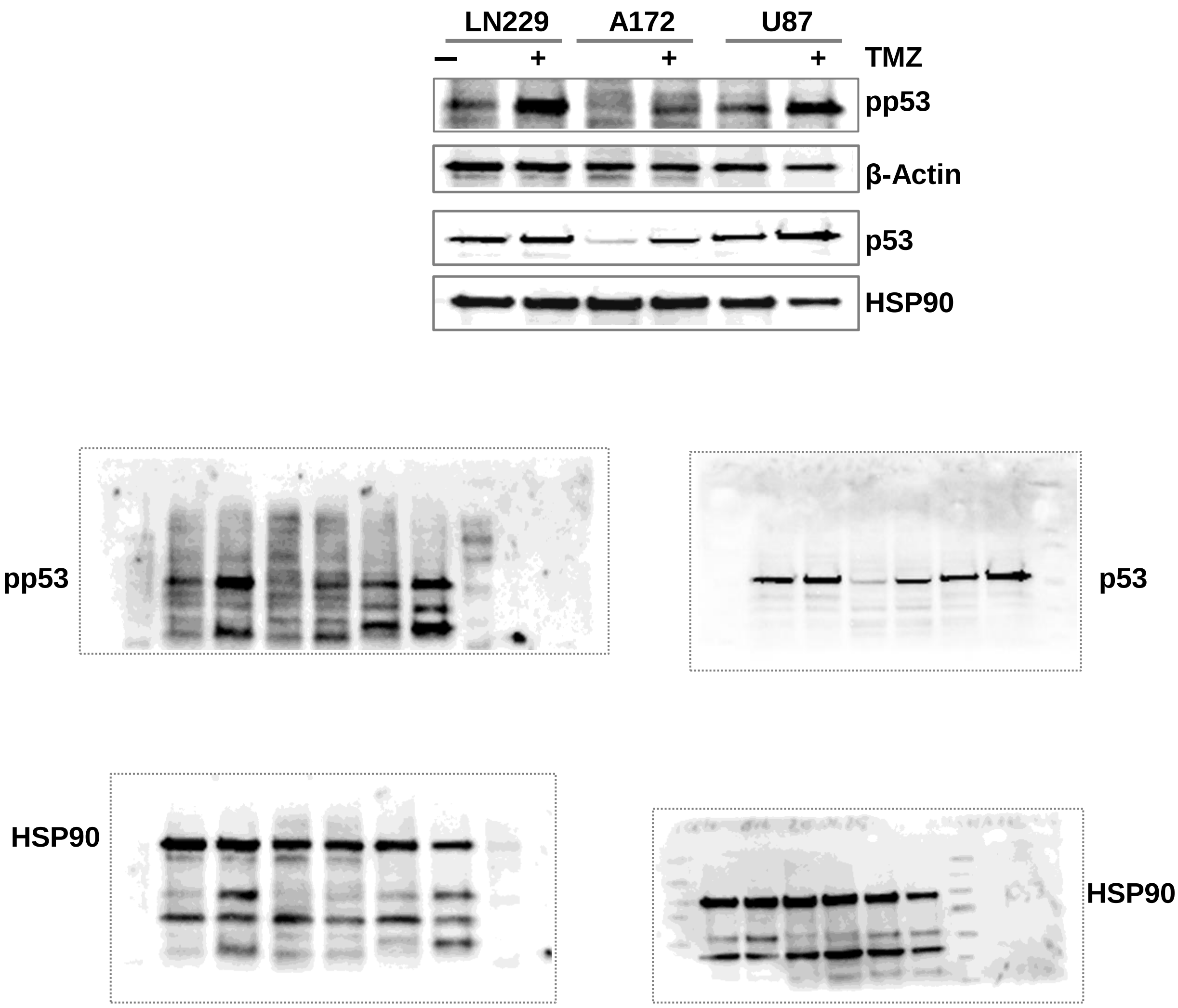
LN229
A172
U87
–
+
+
+
TMZ
pp53
β-Actin
p53
HSP90
pp53
p53
HSP90
HSP90

Fig. S1A

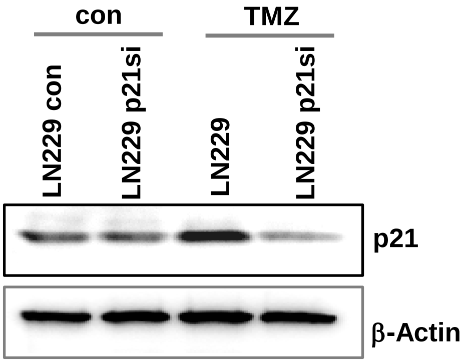

Blots were cut before seperate antibody incubation

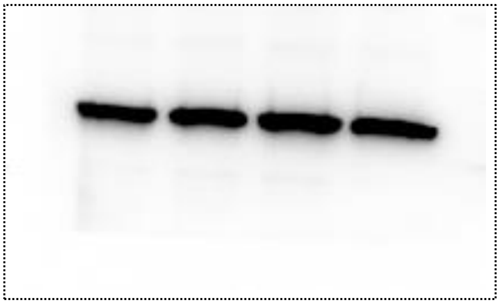

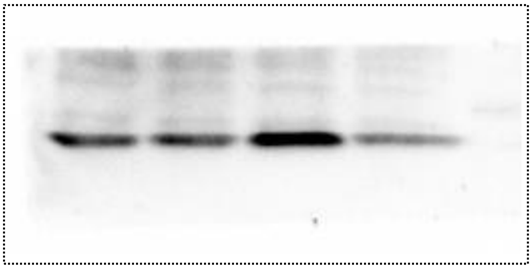

Fig. S8D

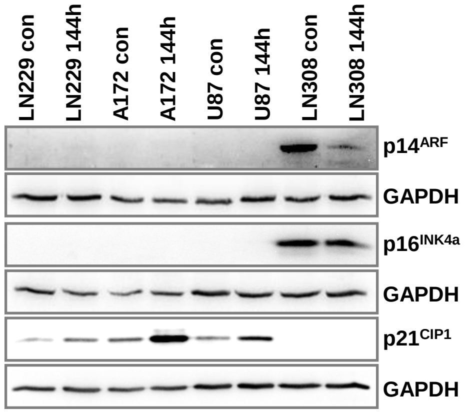

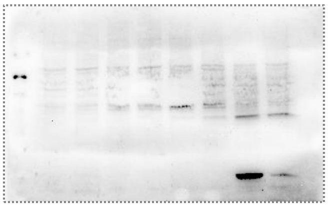

$p14^{ARF}$

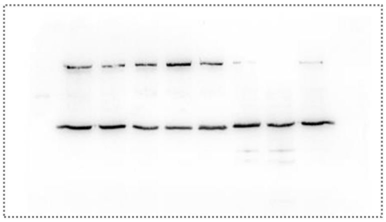

GAPDH

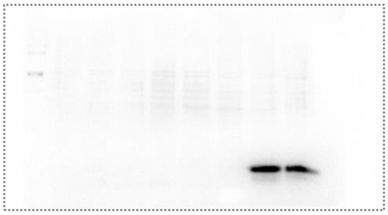

$p16^{INK4a}$

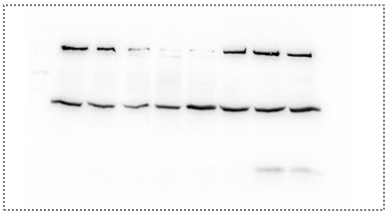

GAPDH

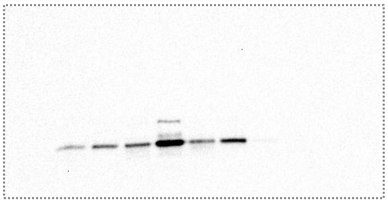

$p21^{CIP1}$

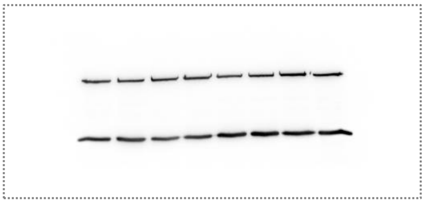

GAPDH

Fig. S11

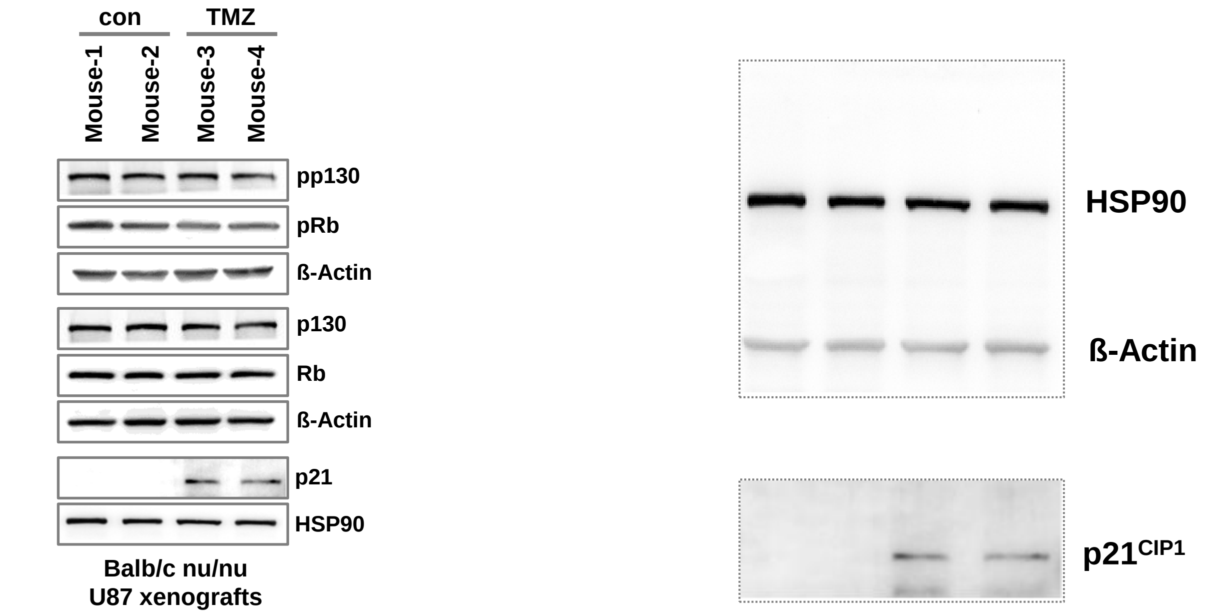

Blots were cut before seperate antibody incubation

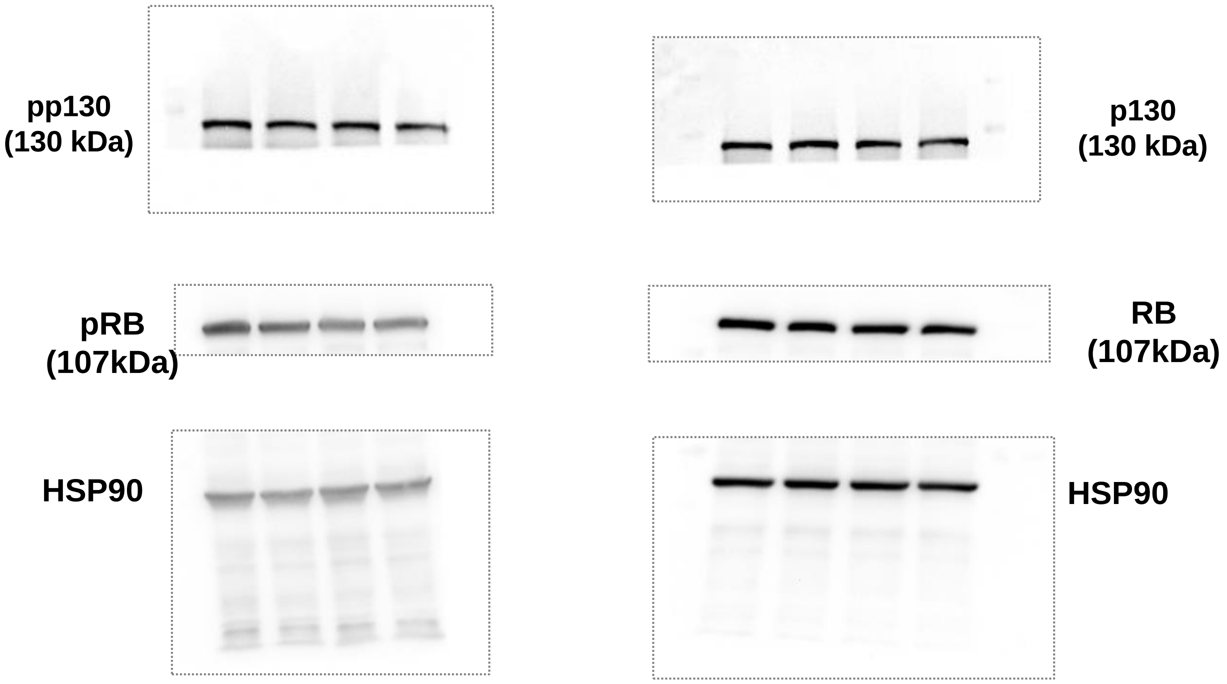

Supplement: Supplementary file 3 — Uncropped western blots [file 41419_2025_7651_MOESM3_ESM.pdf]
